# Safety and immunogenicity of a single-dose adenovirus-vectored rabies vaccine over 1 year in adults and children in Tanzania: interim data from an ongoing, partly randomised, controlled, phase 1b/2 trial

**DOI:** 10.1016/S1473-3099(26)00071-X

**Published:** 2026-08

**Authors:** Adam J Ritchie, Omary Hassan, Nsiande Urasa, Paschal A Apanga, Rinter Kimathi, Wilmina F Kalinga, Ivanny M Mtaka, Reward G Lyimo, Moshi Moshi Shabani, Francisco Orliacq, Adam Berg, ZhiQuan Xiang, Saumu Ahmed, Thabit Athuman, Hildegund C J Ertl, Ally Olotu, Alexander D Douglas, RAB002 study team, Rose Paul Mkumbange, Ibrahim Sasamalo, Aina-Ekisha Kahatano, Safiness Daudi Mchome, Bakari Mwalimu Bakari, Neema Balige, Hania Msami, Mohamed Mohamed, Egbert Stanslaus Kenya

**Affiliations:** a Jenner Institute, University of Oxford, Oxford, UK; b Interventions and Clinical Trials Department, Ifakara Health Institute, Bagamoyo, Tanzania; c Wistar Institute of Anatomy & Biology, Philadelphia, PA, USA

## Abstract

**Background:**

Rabies kills approximately 59 000 people each year. ChAdOx2 RabG, a simian adenovirus-vectored rabies vaccine candidate, has the potential to provide low-cost, single-dose pre-exposure rabies prophylaxis. We aimed to assess the safety and immunogenicity of ChAdOx2 RabG, including in comparison to a currently licensed vaccine.

**Methods:**

We performed a single-centre, age de-escalation, dose-escalation, partly randomised, open-label, phase 1b/2 trial. Healthy adults (18–45 years) and children (2–6 years) who were rabies vaccine-naive and from the semi-urban Bagamoyo area in Tanzania were recruited through a series of community meetings. Adults were first enrolled into a group receiving 2·5 × 10^10^ virus particles of ChAdOx2 RabG (middle dose); after safety review, adults were randomly assigned (3:1) to receive ChAdOx2 RabG (5 × 10^10^ virus particles; full dose) on day 0 or an inactivated rabies virus (IRV) vaccine on day 0 (single-visit IRV). Children were first enrolled into a group receiving 1 × 10^10^ virus particles of ChAdOx2 RabG (low dose), and then into a group receiving the middle dose. After safety review, children were randomly assigned (3:2:2) to receive a full dose of ChAdOx2 RabG on day 0, single-visit IRV, or IRV vaccine on day 0 and day 7 (two-visit IRV). ChAdOx2 RabG was administered intramuscularly at a single anatomical site and IRV intradermally at two anatomical sites. Randomisation was done in blocks by clinical staff using lists generated by an independent statistician. The primary outcome was safety, assessed in the intention-to-treat population. The secondary outcome was rabies virus neutralising antibody (VNA), assessed with a validated assay in participants in the single-visit, full-dose ChAdOx2 RabG groups and the single-visit IRV vaccine groups who attended the nominal 1-year visit. Follow-up is planned for up to 5·5 years after vaccination; here we present data up to 1 year. The trial is registered at ClinicalTrials.gov (NCT04270838).

**Findings:**

Between March 3, 2022, and March 9, 2023, 63 adults (three in the middle-dose ChAdOx2 RabG group, 45 in the full-dose ChAdOx2 RabG group, and 15 in the single-visit IRV group) and 111 children (three each in the low-dose and middle-dose ChAdOx2 RabG groups, 45 in the full-dose ChAdOx2 RabG group, 30 in the single-visit IRV group, and 30 in the two-visit IRV group) were enrolled. Participants reported predominantly mild-to-moderate reactogenicity, most commonly injection-site pain or feverishness, and no serious adverse reactions. In adults, geometric mean VNA titres on day 365 were 2·0 (95% CI 1·4–2·9) after single-visit, full-dose ChAdOx2 RabG vaccination and 0·4 (0·2–0·7) after single-visit IRV vaccination (geometric mean ratio 5·1 [95% CI 2·5–10·4]; p<0·0001). In children, geometric mean VNA titres on day 365 were 6·1 (4·5–8·2) after single-visit, full-dose ChAdOx2 RabG vaccination and 0·7 (0·5–1·1) after single-visit IRV vaccination (geometric mean ratio 8·6 [5·4–13·9]; p<0·0001). In a post-hoc analysis, day-365 VNA titres in children who received ChAdOx2 RabG exceeded titres in those who received two-visit IRV (geometric mean 3·0 [95% CI 2·2–4·1]; geometric mean ratio 2·0 [1·3–3·1]; p=0·0028).

**Interpretation:**

The ChAdOx2 RabG vaccine was safe and well tolerated in Tanzanian adults and children. It achieved robust attainment and maintenance of VNA responses, with titres at day 365 exceeding the correlate of protection (VNA ≥0·5 IU/mL) and titres in licensed vaccine comparator groups. ChAdOx2 RabG might be an option for lower-cost, easier-to-deliver pre-exposure prophylaxis for people in rabies-endemic areas, extending the range of contexts in which pre-exposure prophylaxis is considered a cost-effective option for rabies prevention.

**Funding:**

UK Medical Research Council.

**Translation:**

For the Swahili translation of the abstract see Supplementary Materials section.

**Research in context**

**Evidence before this study**

The shortest WHO-recommended pre-exposure rabies prophylaxis (PrEP) regimen consists of two vaccination visits. We previously showed the immunogenicity and safety of the ChAdOx2 RabG vaccine, a single-visit adenovirus-vectored rabies vaccine, in a phase 1 trial in healthy adults in the UK. We searched PubMed for publications between Jan 1, 1995, and Nov 1, 2025, with the following search terms: (rabies vaccine) AND (pre-exposure) AND ((single-dose) OR (single-visit) OR (abbreviated)) AND (clinical trial), with no language restrictions. Six articles were identified, of which two were related to the development of the ChAdOx2 RabG vaccine reported here. The four other articles described four clinical trials; in each, at least one study group received single-visit PrEP using currently licensed, inactivated rabies virus (IRV) vaccines. Seroconversion measured 14–63 days after single-visit PrEP ranged from 46% to 90%. Three of four trials included boosting via simulated post-exposure prophylaxis (sPEP) 6–28 months after single-visit PrEP. Seroconversion 7 days after sPEP ranged from 95% to 100%. Seroconversion after single-visit vaccination with licensed vaccines was inconsistent, but there were strong and rapid responses to sPEP.

**Added value of this study**

This study is the first clinical trial of ChAdOx2 RabG vaccine in a population from a rabies-endemic area, and the first to include children, a probable target group for future vaccination campaigns. ChAdOx2 RabG vaccine was safe and well tolerated in both adults and children. The immunogenicity of a single dose of ChAdOx2 RabG vaccine robustly exceeded the correlate of protection (measured using a standardised virus neutralisation assay) and exceeded that of single-visit or two-visit intradermal IRV vaccination schedules using currently licensed products. Our study also provides what we believe to be the first characterisation of the immunogenicity in African children of the recently WHO-recommended two-visit intradermal IRV PrEP regimen using a licensed vaccine. Exploratory analysis showed unexpectedly rapid seroconversion after ChAdOx2 RabG vaccination.

**Implications of all the available evidence**

Although highly efficacious, currently licensed rabies vaccines have limitations, including the need for dosing at two or more visits and relatively high manufacturing costs. These factors have contributed to the use of human PrEP being limited in most countries with high burdens of rabies disease, and the associated loss of 59 000 lives per year. Lower-cost, single-visit rabies vaccines might be able to overcome these limitations. This interim report of a phase 1b/2 study demonstrates that the immunogenicity of a single dose of ChAdOx2 RabG vaccine robustly exceeds the correlate of protection and compares favourably with that of a licensed vaccine. Future data from this ongoing trial will provide further information regarding the durability of immunogenicity, including recall responses. ChAdOx2 RabG might also have potential as a component of a future single-visit post-exposure prophylaxis regimen.

## Introduction

Rabies is vaccine-preventable yet causes approximately 59 000 deaths each year, with the highest burdens in Africa and south Asia.^1^, ^2^ Rabies risk varies greatly, both between and within countries, with children living in rural settings tending to have the highest risk.^1^, ^2^, ^3^ Several inactivated rabies virus (IRV) vaccines are licensed for human use, with multiple doses recommended for protection, whether through pre-exposure (PrEP) or post-exposure prophylaxis (PEP), or both. Rabies immunoglobulin is also recommended as part of PEP for vaccine-naive individuals with severe exposure.^3^, ^4^

Most rabies exposures are through dog bites, and decreasing exposure risk by vaccinating dogs is a major focus of the Zero by 30 programme, which aims to eliminate dog-transmitted human rabies by 2030.^5^, ^6^ Sustained dog vaccination coverage of at least 70% is needed to achieve elimination.^3^, ^5^ In many contexts, this coverage is not being achieved.^5^, ^6^ Gavi, the Vaccine Alliance is now supporting improved access to PEP, which is highly effective in preventing rabies if initiated shortly after exposure.^5^, ^6^, ^7^ Delivery of what is effectively an emergency treatment requiring three visits to a health-care facility is programmatically challenging, and a significant burden for bite recipients.

PrEP is not a focus of the Zero by 30 programme, largely because previous economic modelling studies have suggested it is not cost-effective.^3^, ^8^ Population-wide PrEP has, however, been highly effective in campaigns in parts of Peru and the Philippines.^9^ More recent studies have suggested low-cost PrEP regimens could be cost-effective in areas with a high rabies burden, if the possibility of some degree of life-saving benefit of PrEP is considered (previous studies disregarded possible PrEP efficacy and focused purely on PEP cost savings).^9^, ^10^, ^11^ WHO has identified studies of single-visit PrEP and responses to simulated PEP as research priorities.^3^, ^4^

New single-dose rabies vaccines inducing robust protection would be highly desirable, both for PrEP and PEP use. Development is substantially assisted by the acceptance of an immunological correlate of protection (CoP)—namely, a threshold level of virus neutralising antibody (VNA; ≥0·5 IU/mL) in an internationally standardised assay.^12^, ^13^ For PEP in vaccine-naive recipients, the speed of seroconversion is key, with current regimens achieving a VNA of 0·5 IU/mL or higher in more than 95% of participants 14 days after initiation of vaccination.^14^ For PrEP, the duration of efficacy is more important than speed. Changes in PrEP regimen recommendations from the past decade, have been based on the demonstration of robust anamnestic responses to a simulated booster PEP (sPEP), with VNA titres of 0·5 IU/mL or higher within 7 days of a single dose of a licensed vaccine.^3^, ^15^, ^16^, ^17^

ChAdOx2 RabG, a single-dose rabies vaccine candidate, is a replication-incompetent simian-adenovirus-vectored product encoding the rabies virus glycoprotein.^18^ We have previously reported the results of a first-in-human study of this vaccine, showing it was safe, well tolerated, and immunogenic in adults in the UK.^19^ The cost of manufacturing vaccines based on this vaccine platform is low.^20^

Here, we report results from an ongoing phase 1b/2 study of the safety (primary objective) and immunogenicity (secondary and exploratory objectives) of ChAdOx2 RabG in adults and children in Tanzania. Adult and child comparator groups received IRV vaccination intradermally at two anatomical sites in a single visit (day 0). Although not a WHO-recommended regimen, this regimen is believed to confer some protection against rabies virus infection and might be the most cost-effective existing PrEP option,^3^, ^9^, ^16^ making it the most relevant comparator to ChAdOx2 RabG in rabies-endemic contexts where rabies PrEP is currently unused on the basis of cost. Moreover, we considered this regimen a minimum bar that a new candidate should outperform to merit further evaluation. An additional paediatric comparator group received intradermal IRV vaccination at two anatomical sites on both day 0 and day 7, which is the lowest-cost PrEP regimen recommended by WHO.^3^

This report includes immunogenicity data up to and including the nominal 1-year visit, plus all available safety data at the time of manuscript preparation. These data include the key prespecified immunological outcome measures, which were designed to assess suitability for use as PrEP by inferring probable efficacy, both with and without booster PEP. In addition, we report the speed of seroconversion in a subset of participants, to assess the possible suitability for use of ChAdOx2 RabG as single-visit PEP for vaccine-naive recipients.

## Methods

### Study design

The RAB002 study is an ongoing, age de-escalation, dose-escalation, partly randomised, open-label, phase 1b/2 trial conducted in a single centre (Ifakara Health Institute Clinical Trial Facility) in Bagamoyo, Tanzania, and sponsored by the University of Oxford, Oxford, UK. Details of the design and manufacture of the ChAdOx2 RabG vaccine and phase 1 clinical trial results have been published.^18^, ^19^ Verorab (Sanofi Pasteur, Lyon, France) was used as the IRV vaccine comparator and for sPEP.

Over the full course of the study, participants will be followed up for up to 5·5 years. Here, we report the results of all visits during and including the nominal year 1 visit (day 365, within –92 or +181 days) and all sPEP visits that took place in the first 1·5 years of the study. The study originally included 1 year of follow-up, but was later amended (after the availability of encouraging data from the first-in-human study)^19^ to include follow-up for up to 5·5 years. All but one participant consented to the extended follow-up: that participant completed the first year of follow-up. During community meetings, recommendations were solicited from participants and members of the community on practical aspects of trial implementation. In addition, end-of-trial meetings will be held with participants to develop community-centred materials for disseminating research findings.

The study was approved by the Tanzania Medicines and Medical Devices Authority (reference number TMDA0020/CTR/0004/02) and the Tanzanian National Institute for Medical Research (reference number MIMR/HQ/R.8a/Vol.IX/3552). It was also approved by the Ifakara Health Institute institutional review board (reference number 12–2021) and Oxford Tropical Research Ethics Committee (reference number 8–20). The study is registered at ClinicalTrials.gov (NCT04270838) since Feb 17, 2020. The trial is fully recruited and follow-up is ongoing. The trial is being conducted in accordance with the principles of the Declaration of Helsinki and Good Clinical Practice.

### Participants

Healthy adults and children with no history of rabies vaccination living in the semi-rural Bagamoyo district in Tanzania were recruited after community-based sensitisation meetings that provided information about the study. Written informed consent was obtained from adult participants and the parents or guardians of child participants. Key inclusion criteria were age (18–45 years for adults, 2–6 years for children), and planned long-term local residency, whereas key exclusion criteria included specified comorbidities, receipt of a rabies vaccine at any time, or receipt of any adenovirus-vectored vaccine in the past 6 months. Full details of the inclusion and exclusion criteria can be found in the protocol in appendix 2 (pp 66–68). Sex (male or female) and ethnicity data were self-reported.

### Randomisation and masking

Randomisation processes, groups, and regimens are described in detail in the protocol (appendix 2 pp 38, 75–76). Participant enrolment into non-randomised adult and child dose-escalation groups (adult middle-dose ChAdOx2 RabG group and child low-dose and middle-dose ChAdOx2 RabG groups) was conducted by clinical staff. Random assignment to other groups was performed by a pharmacist independent of the clinical team, using master lists generated by an independent statistician (who was not otherwise involved in the study). Randomisation was done in balanced blocks of four (adult) or seven (child) participants. Adults were randomly assigned (3:1) to receive a full dose of ChAdOx2 RabG on day 0 or IRV vaccination on day 0 (single-visit IRV). Children were randomly assigned (3:2:2) to receive a full dose of ChAdOx2 RabG on day 0, single-visit IRV vaccine, or IRV vaccine on day 0 and day 7 (two-visit IRV).

To assess recall responses to sPEP, participants with a most recently available VNA of less than 0·5 IU/mL before an annual visit (ie, here, the year 1 visit) were randomly assigned 1:1 to receive sPEP either at that visit or at the end of the study. Details of randomisation are provided in appendix 2 (p 76). Clinical staff and participants were not masked to treatment group allocation or to the allocation of timing of sPEP, whereas laboratory staff performing and analysing VNA assays for secondary outcomes were masked. No other masking took place.

### Procedures

All participants received a vaccine on day 0. ChAdOx2 RabG was administered by 0·31 mL intramuscular injection at a single anatomical site in the deltoid area of the non-dominant arm. Dose-escalation groups received a low dose (1 × 10^10^ virus particles) or a middle dose (2·5 × 10^10^ virus particles) of ChAdOx2 RabG; all other ChAdOx2 RabG vaccinees received a full dose of 5 × 10^10^ virus particles. For single-visit IRV vaccine comparator groups, Verorab was administered by intradermal injection of 0·1 mL at each of two anatomical sites on day 0. Participants allocated to receive two-visit IRV vaccine also received the same on day 7. Two batches of Verorab were used in the study, with potency of 4·7 IU and 8·2 IU per 0·5 mL vial, such that the adult comparator group received a total dose of 1·9 IU whereas children in the comparator groups received a total dose of 1·9 IU or 3·3 IU per visit.

Where applicable, sPEP was delivered at the day 365 visit (then designated day sPEP+0). sPEP consisted of a WHO-recommended regimen for booster PEP in previous PrEP recipients (ie, single-visit, intradermal administration of 0·1 mL of IRV vaccine at each of four anatomical sites). The Verorab IRV vaccine batch used for sPEP had a potency of 8·2 IU per 0·5 mL vial, such that participants received a total dose of 6·6 IU. Participants who maintained a VNA of 0·5 IU/mL or more throughout the 5-year study period will not undergo sPEP until the end of the study, allowing estimation of the duration of probable PrEP efficacy independent of booster-PEP.

After vaccination, participants attended the study centre or were visited at home by study staff at the following nominal timepoints: days 1–7 (daily), then days 14, 28, 56, 182, and 365. Blood samples for immunological assays were taken on nominal days 0, 7, 14, 28, 56, 182, and 365 from all groups. The first 15 participants enrolled in each of the full-dose ChAdOx2 RabG and single-visit IRV vaccine groups were designated as the immunology subset and underwent additional blood processing at specific visits. Samples were also collected at sPEP+7 and sPEP+14 for participants undergoing sPEP at 1 year (for whom the nominal day 365 visit constituted sPEP+0). Prespecified immunogenicity analyses used VNA measured with the rapid fluorescent focus inhibition test as previously described,^19^ with a limit of detection of 0·22 IU/mL, at the Wistar Institute (Philadelphia, PA, USA), in accordance with the principles of Good Clinical Laboratory Practice. Methods for exploratory immunology assays are provided in appendix 2 (pp 3–6). These included higher sensitivity VNA (with limit of detection of 0·03 IU/mL, at days 7 and 14), ELISAs (for total anti-rabies glycoprotein IgG, subclasses, and avidity), enzyme-linked immunospot assays (ELISPOTs; for rabies glycoprotein-specific interferon-γ-producing T cells and memory B cells), and anti-adenovirus-vector neutralising antibody assays. Some assays (including ELISPOTs and higher-sensitivity VNA at days 7 and 14) were carried out for the immunology subset only. The list of protocol deviations, including one dosing error, one sampling error, 28 missed study visits, 14 visits outside the visit window, two missing samples, and one participant who underwent no sampling after the day 28 visit, is in appendix 2 (p 16).

### Outcomes

The primary outcome was safety. Solicited adverse events were assessed from the day of vaccination (day 0) to 7 days after vaccination (day 6), and unsolicited adverse events were assessed from day 0 to day 28. Serious adverse events were assessed throughout the trial. Vital signs were taken at days 0, 1, 7, and all subsequent visits. Full blood count and renal function tests were conducted at days 0, 7, 14, 28, 56, and 182. Severity of adverse events was assessed by the investigators using protocol-specified grading systems (appendix 2 pp 91–94). The principal investigator was responsible for determining relatedness.

The secondary objective was to assess the immunogenicity of ChAdOx2 RabG compared with that of a licensed IRV vaccine, with VNA as the outcome measure, assessed at days 28, 56, 182, and 365, and sPEP+7 visits. Exploratory outcome measures were total IgG ELISA (measured at days 0, 14, 28, 182, and 365), IgG subclass ELISA (at days 0 and 28), avidity ELISA (at day 28), memory B-cell ELISPOT (at days 0 and 365), interferon-γ T-cell ELISPOT (at days 0 and 14), and anti-vector neutralising antibody titres (at days 0, 28, and 365). Of these, total IgG ELISA, interferon-γ T-cell ELISPOT, and anti-vector neutralising antibody were prespecified exploratory outcomes. Other exploratory outcomes were post-hoc. Timepoints were not prespecified for any of the exploratory outcome measures.

### Statistical analysis

OpenClinica version 3.1 was used for electronic data capture and clinical data management. Prespecified statistical analysis was conducted according to a statistical analysis plan (appendix 2 pp 121–130). Descriptive analyses of baseline characteristics and safety data were carried out using Stata version 14.2 and presented using tables and graphs produced in Microsoft Excel 2021. Immunology data were analysed using Stata, tables prepared using Microsoft Excel, and figures prepared using GraphPad Prism version 10.2.1.

In keeping with the primary objective of assessing safety, the sample size was chosen based on the investigators' opinion of an adequate number of participants to facilitate further clinical evaluation in subsequent trials. Under the so-called rule of three,^21^ the chosen sample size resulted in a 95% probability of detecting an adverse event with a true event rate of one in 15 (ie, three of 45) after full-dose ChAdOx2 in either adults or children.

Safety was assessed in the intention-to-treat (ITT) population. Within the overall secondary trial objective of characterising immunogenicity based on VNA, a hierarchy of analyses was prespecified in the statistical analysis plan (appendix 2 p 121–130) and is summarised in appendix 2 (pp 2–3). The primary immunological hypothesis test for each age group was a two-tailed comparison of VNA responses among all participants attending the nominal 1-year visit in single-visit, full-dose ChAdOx2 RabG versus single-visit IRV groups (ie, the ITT population, modified only by the exclusion of participants lost to follow-up [hereafter referred to as the mITT population]). After log_10_ transformation and d'Agostino–Pearson normality testing, comparisons were performed by a Mann–Whitney test. Between-group differences in geometric mean titres of VNA were calculated as geometric mean ratios (with 95% CIs). We also calculated non-parametric estimates of between-group differences—namely, ratios of medians and exponentiated bounds of the Wilcoxon and Mann–Whitney 95% CIs for the Hodges–Lehmann estimate of the difference between group medians of the log_10_-transformed data (appendix 2 pp 27).

Exploratory and post-hoc analyses included comparisons (for each age group) of VNA at each additional timepoint it was measured (comparing ChAdOx2 with single-visit and, for children, two-visit IRV vaccine groups in the mITT population, by Mann–Whitney, Welch's, or Student's *t* tests, as appropriate, after d'Agostino–Pearson normality testing and F-tests for homogeneity of variance), and of interferon-γ T-cell ELISPOT at day 14 (full-dose ChAdOx2 RabG *vs* single-visit IRV vaccine, by Mann–Whitney test and Hodges–Lehmann estimate of difference between group medians, in the immunology subset population). Exploratory analyses also assessed the duration of maintenance of a VNA of more than 0·5 IU/mL (Kaplan–Meier survival in the mITT population), the relationship between VNA and total IgG ELISA (at days 28 and 365, by linear regression, in the mITT population), and the relationship between baseline and day 28 anti-vector neutralising antibody titres (among the full-dose ChAdOx2 RabG-recipient mITT population, using Spearman's rank correlation).

Oversight was done by an independent safety monitoring committee composed of three senior clinicians, including an independent Tanzanian paediatrician. Interim analyses of the safety data by the safety monitoring committee took place before the first paediatric participant was vaccinated, and before escalation to the full dose in children. Holding rules based on the occurrence of serious or severe adverse events were defined in the study protocol, as were additional interim analyses of safety data performed by the chief and principal investigators (appendix 2 pp 61–63).

### Role of the funding source

The funder reviewed the study design but had no role in data collection, data analysis, data interpretation, or writing of the report.

## Results

**Figure 1:**
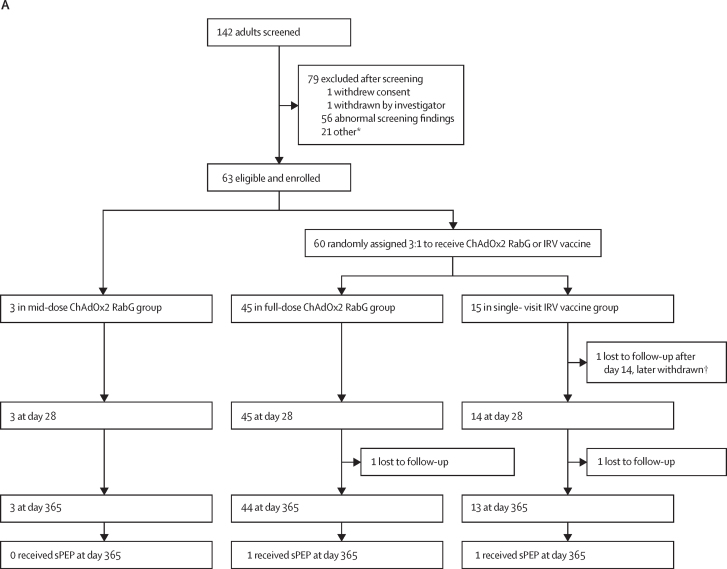

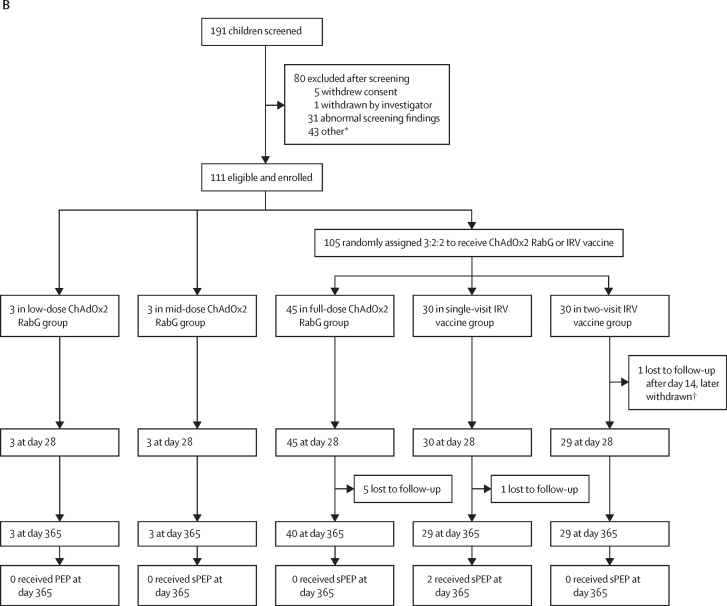
Participant flow Simplified CONSORT diagrams for (A) adults (aged 18–45 years) and (B) children (aged 2–6 years), showing the study design and participant flow at key timepoints. More information is provided in appendix 2 (pp 7–8), including additional timepoints and simulated booster PEP allocation details. Numbers shown include 12 adults (three in the middle-dose ChAdOx2 RabG group, seven in full-dose ChAdOx2 RabG group, and two in the single-visit IRV group) who attended the day 365 visit 1–4 days outside the permitted window for day 365, without having lost contact with the study team, and one child in the single-visit IRV vaccine group who was temporarily lost to follow-up and attended the day 365 visit 70 days outside the permitted window. The investigators agreed prospectively (before data analysis) that data from visits 1–4 days outside the permitted window should be considered as part of the immunogenicity analyses for the day 365 visit, whereas data from the child should not be (appendix 2 p 16). Day 365 visits were skewed towards the end of the permissible window (days 273–546), with a median visit day of 523 for adults and 432 for children. IRV=inactivated rabies virus. sPEP=simulated post-exposure prophylaxis. *Other refers to participants who did not return after screening or those who were screened but the protocol-defined window between screening and enrolment expired before they could be enrolled; for children it also includes cases in which Essential Programme on Immunization vaccination status could not be verified and if a child presented a difficult venopuncture. †This participant's last visit was at day 14 and contact was lost before the day 28 visit; therefore, they were withdrawn after an attempt to contact them.

Adult participants were enrolled between March 3 and June 3, 2022 (figure 1A). Of 142 adults assessed for eligibility, 63 were enrolled (including three in the middle-dose ChAdOx2 RabG group and 60 who were randomly assigned to the full-dose ChAdOx2 RabG [n=45] or single-visit IRV vaccine [n=15] groups). After interim safety data analysis by the safety monitoring committee, child participants were enrolled between Sept 6, 2022, and March 9, 2023 (figure 1B). Of 191 children assessed for eligibility, 111 were enrolled (including three each in the low-dose and middle-dose ChAdOx2 RabG groups and 105 who were randomly assigned to the full-dose ChAdOx2 RabG group [n=45], single-visit IRV group [n=30], or two-visit IRV group [n=30]). The baseline characteristics of adult and child participants are shown in Table 1, Table 2.

**Table 1:** Baseline characteristics of adult study participants

		**Middle-dose ChAdOx2 RabG (n=3)**	**Full-dose ChAdOx2 RabG (n=45)**	**Single-visit IRV (n=15)**	**All adults (n=63)**
Sex*					
	Female	0	6 (13%)	1 (7%)	7 (11%)
	Male	3 (100%)	39 (87%)	14 (93%)	56 (89%)
Age, years		24 (20–29)	24 (22–28)	23 (21–31)	24 (21–29)
Education					
	College	0	3 (7%)	1 (7%)	4 (6%)
	Secondary	3 (100%)	19 (42%)	6 (40%)	28 (44%)
	Primary	0	18 (40%)	7 (47%)	25 (40%)
	Non-formal education	0	5 (11%)	1 (7%)	6 (10%)

Data are n (%) or median (IQR). Middle-dose ChAdOx2 RabG consisted of 2·5 × 10^10^ virus particles; full-dose ChAdOx2 RabG consisted of 5 × 10^10^ virus particles. IRV=inactivated rabies virus.

* Sex was self-reported by participants.

**Table 2:** Baseline characteristics of child study participants

		**Low-dose ChAdOx2 RabG (n=3)**	**Middle-dose ChAdOx2 RabG (n=3)**	**Full-dose ChAdOx2 RabG (n=45)**	**Single-visit IRV (n=30)**	**Two-visit IRV (n=30)**	**All children (n=111)**
Sex*							
	Female	1 (33%)	2 (67%)	19 (42%)	12 (40%)	13 (43%)	47 (42%)
	Male	2 (67%)	1 (33%)	26 (58%)	18 (60%)	17 (57%)	64 (58%)
Age, years		4 (3–4)	5 (3–5)	3 (2–4)	3 (2–4)	3 (2–4)	3 (2–4)
Age group, years							
	2	0 (0%)	0 (0%)	16 (36%)	8 (27%)	13 (43%)	37 (33%)
	3	1 (33%)	1 (33%)	9 (20%)	10 (33%)	8 (27%)	29 (26%)
	4	2 (67%)	0 (0%)	11 (24%)	6 (20%)	5 (17%)	24 (22%)
	5	0 (0%)	2 (67%)	9 (20%)	6 (20%)	4 (13%)	21 (19%)
Education							
	Primary	0 (0%)	0 (0%)	2 (4%)	2 (7%)	0 (0%)	4 (4%)
	Not applicable	3 (100%)	3 (100%)	43 (96%)	28 (93%)	30 (100%)	107 (96%)

Data are n (%) or median (IQR). Low-dose ChAdOx2 RabG consisted of 1 × 10^10^ virus particles; middle-dose ChAdOx2 RabG consisted of 2·5 × 10^10^ virus particles; full-dose ChAdOx2 RabG consisted of 5 × 10^10^ virus particles. IRV=inactivated rabies virus.

* Sex was self-reported by participants or their parents or guardians.

Acceptable time windows for each study visit are defined in the protocol in appendix 2 (pp 76–80). Of note, nominal day 365 visits were skewed towards the later end of the permissible window (days 273–546), to allow reporting of previous VNA results and hence sPEP randomisation. The median day after vaccination that this visit actually took place was 532 days for adults and 432 days for children. Details of missed visits, out-of-window visits, and other deviations from the protocol are in appendix 2 (pp 7–8, 16).

**Figure 2:**
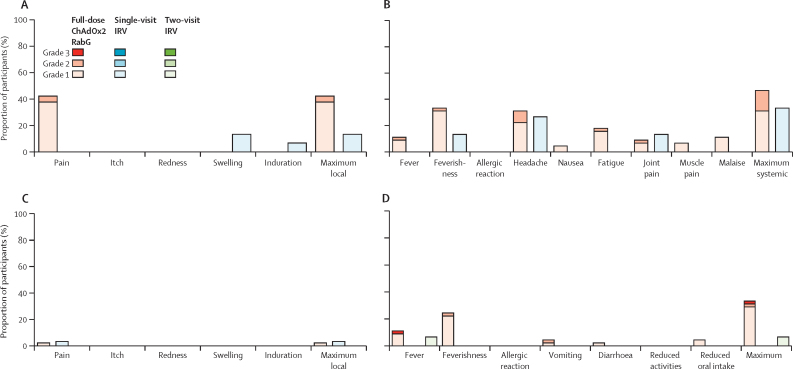
Solicited adverse events Solicited (A) local and (B) systemic adverse events in adult participants, and solicited (C) local and (D) systemic adverse events in children, in groups receiving full-dose ChAdOx2 RabG (45 adults and 45 children), single-visit IRV vaccine (15 adults and 30 children), and two-visit IRV vaccine (30 children only). Data for lower-dose groups for ChAdOx2 RabG are provided in appendix 2 (p 9). Values reflect the maximum severity of each reaction reported for each participant over 7 days after vaccination. To provide a global view of reactogenicity, the grade of the most severe local and systemic reaction reported by each individual (if any) is also shown (as maximum local and maximum systemic). IRV=inactivated rabies virus.

Among adult recipients of the full-dose ChAdOx2 RabG vaccine, reactogenicity was mild-to-moderate and transient, with 94% (81 of 86) of solicited adverse events occurring and resolving within 48 h of vaccination. The most frequent local and systemic adverse events in the full-dose ChAdOx2 RabG group were injection-site pain (42%; 19 of 45) and feverishness (33%; 15 of 45), respectively (table 3; figure 2). In this group, unsolicited adverse events were reported by a minority of participants, with two (4%) of 45 participants who reported an event potentially related to vaccination, while 14 (31%) of 45 reported an event assessed as having no relationship or being unlikely to be related to vaccination (appendix 2 p 20). Three serious adverse events (miscarriage) were reported among adults in the full-dose ChAdOx2 RabG group between the start of the study in March 2, 2022, and Sept 30, 2025. Each occurred 105 days or more after vaccination and were considered unlikely to be related to the vaccination (appendix 2 p 17).

**Table 3:** Solicited adverse events

	**Adults**						**Children**									
	Middle-dose ChAdOx2 RabG (n=3)		Full-dose ChAdOx2 RabG (n=45)		Single-visit IRV (n=15)		Low-dose ChAdOx2 RabG (n=3)		Middle-dose ChAdOx2 RabG (n=3)		Full-dose ChAdOx2 RabG (n=45)		Single-visit IRV (n=30)		Two-visit IRV (n=30)	
	Number of participants reporting events	Number of events	Number of participants reporting events	Number of events	Number of participants reporting events	Number of events	Number of participants reporting events	Number of events	Number of participants reporting events	Number of events	Number of participants reporting events	Number of events	Number of participants reporting events	Number of events	Number of participants reporting events	Number of events
**Local solicited adverse events**																
Any local solicited adverse event	1 (33%)	1	19 (42%; 28–57)	30	2 (13%; 3–45)	9	1 (33%)	3	0	0	1 (2%; 0–15)	1	1 (3%; 0–22)	1	0	0
Pain	1 (33%)	1	19 (42%; 28–57)	30	0	0	1 (33%)	3	0	0	1 (2%; 0–15)	1	1 (3%; 0–22)	1	0	0
Swelling	0	0	0	0	2 (13%; 3–45)	6	0	0	0	0	0	0	0	0	0	0
Induration	0	0	0	0	1 (7%; 1–42)	3	0	0	0	0	0	0	0	0	0	0
**Systemic solicited adverse events**																
Any systemic solicited adverse event	1 (33%)	1	21 (47%; 32–62)	56	5 (33%; 13–63)	9	2 (67%)	3	0	0	15 (33%; 21–49)	21	0	0	2 (7%; 2–25)	2
Fever	0	0	5 (11%; 5–25)	5	0	0	1 (33%)	1	0	0	5 (11%; 5–25)	5	0	0	2 (7%; 2–25)	2
Feverishness	1 (33%)	1	15 (33%; 21–49)	15	2 (13%; 3–45)	2	0	0	0	0	11 (24%; 14–40)	11	0	0	0	0
Headache	0	0	14 (31%; 19–47)	14	4 (27%; 9–57)	5	NA	NA	NA	NA	NA	NA	NA	NA	NA	NA
Nausea	0	0	2 (4%; 1–17)	2	0	0	NA	NA	NA	NA	NA	NA	NA	NA	NA	NA
Fatigue	0	0	8 (18%; 9–32)	8	0	0	NA	NA	NA	NA	NA	NA	NA	NA	NA	NA
Joint pain	0	0	4 (9%; 3–22)	4	2 (13%; 3–45)	2	NA	NA	NA	NA	NA	NA	NA	NA	NA	NA
Muscle pain	0	0	3 (7%; 2–19)	3	0	0	NA	NA	NA	NA	NA	NA	NA	NA	NA	NA
Malaise	0	0	5 (11%; 3–22)	5	0	0	NA	NA	NA	NA	NA	NA	NA	NA	NA	NA
Vomiting	NA	NA	NA	NA	NA	NA	0	0	0	0	2 (4%; 1–17)	2	0	0	0	0
Diarrhoea	NA	NA	NA	NA	NA	NA	0	0	0	0	1 (2%; 0–15)	1	0	0	0	0
Reduced activities	NA	NA	NA	NA	NA	NA	1 (33%)	1	0	0	0	0	0	0	0	0
Reduced oral intake	NA	NA	NA	NA	NA	NA	1 (33%)	1	0	0	2 (4%; 1–17)	2	0	0	0	0

Solicited adverse events by group for 7 days after vaccination (days 0–6). Data are number of participants reporting event (%; 95% CI), and number of events reported. 95% CIs are not reported where no events occurred, or for dose-escalation groups for which n=3. Data separated based on sex are available in appendix 2 (pp 18–19). Solicited adverse event data are available for all enrolled participants (including the two individuals who were lost to follow-up between days 14 and 28, because this was after the period of collection of solicited adverse events). Low-dose ChAdOx2 RabG consisted of 1 × 10^10^ virus particles; middle-dose ChAdOx2 RabG consisted of 2·5 × 10^10^ virus particles; full-dose ChAdOx2 RabG consisted of 5 × 10^10^ virus particles. IRV=inactivated rabies virus. NA=not applicable as data not gathered for this age group.

Among child recipients of the full-dose ChAdOx2 RabG vaccine, reactogenicity was mostly mild-to-moderate and transient, with 91% (20 of 22) of adverse events occurring and resolving within 48 h of vaccination. Local reactogenicity was minimal (only one [2%] of 45 recipients reported a local event, which was injection-site pain). The most frequent systemic adverse event was feverishness (24%; 11 of 45; table 3; figure 2). In this group, unsolicited adverse events were reported by a minority of participants, with nine (20%) of 45 who reported an event potentially related to vaccination, while four (9%) of 45 reported an event assessed as having no relationship or being unlikely to be related to vaccination (appendix 2 p 21). One (2%) participant in the full-dose group had a grade 3 (severe, but not serious) fever, which resolved within 1 day with antipyretic medication. No serious adverse events were reported among children.

No serious adverse events have been reported in any other groups in the study. In the low-dose and middle-dose ChAdOx2 RabG groups, adverse events were limited (appendix 2 pp 9, 18–23), and considered acceptable for dose escalation to the full dose in both adults and children. Adverse events in the groups receiving single-visit or two-visit vaccination with licensed IRV were limited (figure 2; appendix 2 pp 9, 18–23).

**Figure 3:**
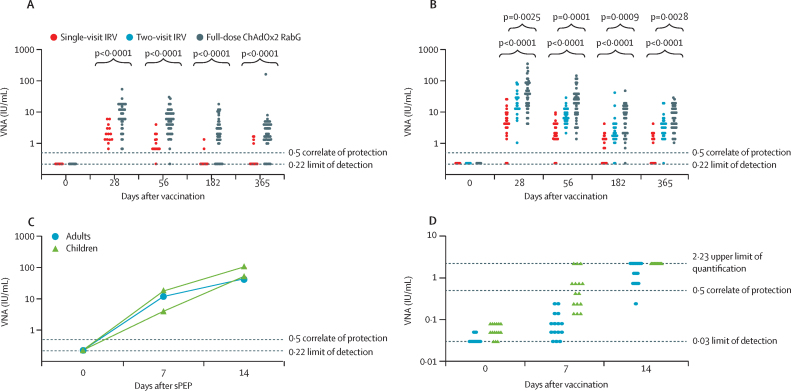
VNA responses VNA levels for (A) adult and (B) paediatric participants. Nominal day 365 visits were skewed towards the end of the permissible window (days 273–546), with a median actual visit day of 523 for adults and 432 for children. p values are from Mann–Whitney tests between groups receiving ChAdOx2 RabG and IRV vaccine at each visit. (C) VNA responses to sPEP given at the nominal day 365 visit to one adult and two children, all of whom had received single-visit IRV primary vaccination. (D) Early VNA responses (days 0–14 after primary vaccination) for adults and children in the immunology subset, which included the first 15 participants enrolled in each of the full-dose and single-visit IRV vaccine groups, as measured using a higher sensitivity assay. appendix 2 (p 10) shows VNA data separated by sex. appendix 2 (p 11) shows Kaplan–Meier plots of duration of maintenance of VNA ≥0·5 IU/mL. appendix 2 (pp 27–28) provides further details of inter-group VNA comparisons. IRV=inactivated rabies virus. sPEP=simulated booster post-exposure prophylaxis. VNA=virus neutralising antibody.

Rabies VNA levels of 0·5 IU/mL or more indicated a satisfactory response to vaccination and likely protection from rabies,^9^, ^16^ and such levels are hereafter described as seropositive. All participants were seronegative at baseline. All adult participants were seropositive by day 28 after a single middle dose (n=3) or full dose (n=45) of ChAdOx2 RabG or single-visit IRV (n=30; figure 3; appendix 2 p 25). All child participants who received a single low dose (n=3), middle dose (n=3), or full dose (n=45) of ChAdOx2 RabG vaccine or two-visit IRV vaccine, and 27 (90%) of 30 children who received single-visit IRV vaccine, were seropositive by day 28 (figure 3; appendix 2 p 26). Of the three single-visit IRV child recipients who were seronegative at day 28, two were seropositive by day 56 (both female, aged 3 and 4 years). The other participant (male, aged 5 years) was seronegative until sPEP.

At the nominal day 365 visit, VNA titres were significantly higher in full-dose ChAdOx2 RabG recipients than in single-visit IRV recipients, both in adults and children (prespecified primary hypothesis test). In adults, geometric mean VNA titres on day 365 were 2·0 (95% CI 1·4–2·9) after full-dose ChAdOx2 RabG vaccination and 0·4 (0·2–0·7) after single-visit IRV vaccination (geometric mean ratio 5·1 [95% CI 2·5–10·4]; p<0·0001 by two-tailed Mann–Whitney test). In children, geometric mean VNA titres on day 365 were 6·1 (4·5–8·2) after full-dose ChAdOx2 RabG vaccination and 0·7 (0·5–1·1) after single-visit IRV vaccination (geometric mean ratio 8·6 [5·4–13·9]; p<0·0001). Post-hoc analysis demonstrated that, at the nominal day 365 visit, VNA responses among child ChAdOx2 RabG recipients were also higher than those in the two-visit IRV group (geometric mean 3·0 [95% CI 2·2–4·1]; geometric mean ratio 2·0 [95% CI 1·3–3·1]; p=0·0028 by two-tailed Mann–Whitney test). Additional post-hoc analyses demonstrated significantly higher VNA responses to ChAdOx2 RabG than to comparator groups at other timepoints (figure 3; appendix 2 pp 27–28).

Among adults, 38 (86% [95% CI 73–95]) of 44 full-dose ChAdOx2 recipients were seropositive at the nominal day 365 visit, as compared with four (31% [9–61]) of 13 single-visit IRV recipients (appendix 2 p 25). Among children, all 38 (100% [91–100]) full-dose ChAdOx2 recipients, 16 (57% [37–76]) of 28 single-visit IRV recipients, and 28 (97% [82–100]) of 29 two-visit IRV recipients were seropositive at the nominal day 365 visit (appendix 2 p 26).

All participants assigned to receive sPEP at the nominal day 365 visit mounted satisfactory responses (VNA of ≥0·5 IU/mL; one adult and two child participants, all from the single-visit IRV groups; figure 3). Calculation of probable boosted protection was limited by the small number of sPEP recipients (appendix 2 pp 29–30). Participants not known to have a VNA of less than 0·5 IU/mL at the nominal day 365 visit will receive sPEP later in the trial.

To explore the potential of the ChAdOx2 RabG vaccine for PEP, we performed additional VNA analysis on days 0, 7, and 14 on samples from the immunology subset of ChAdOx2 RabG recipients, using a higher sensitivity VNA assay. At day 7, 80% (12 of 15) of adults and 100% (15 of 15) of children had detectable responses (ie, increases from day 0). At day 14, 93% (14 of 15) of adults and all (15 of 15) children were seropositive, with a VNA of 0·5 IU/mL or more (figure 3).

**Figure 4:**
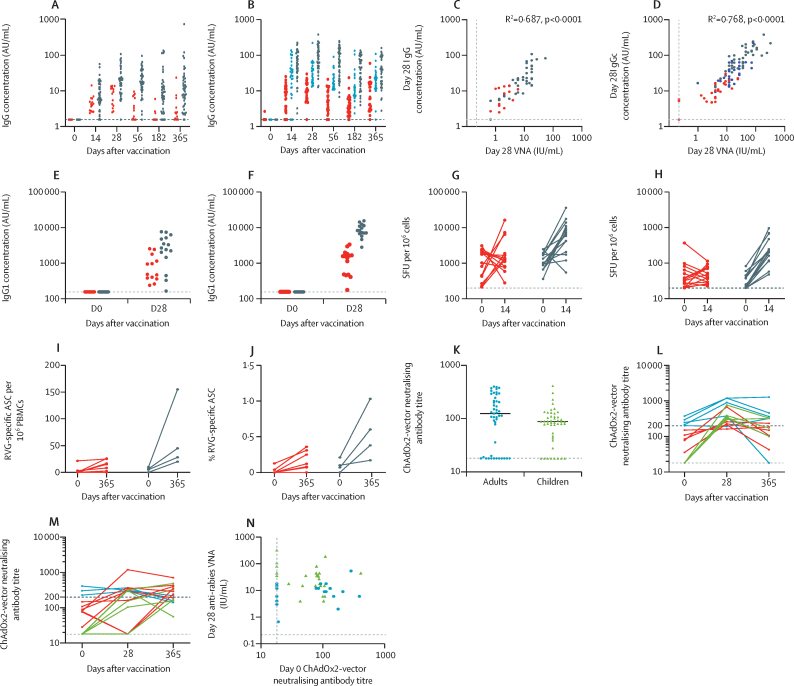
Exploratory immunology outcomes (A–J) Rabies-specific exploratory immunology results for participants who received either a single-visit IRV (red), two-visit IRV (blue), or single-visit, full-dose ChAdOx2 RabG (black) primary vaccination. Dotted lines represent the limit of detection. Anti-rabies virus glycoprotein total IgG responses at each tested nominal timepoint for (A) adults and (B) children. All participants were negative at day 0, except for one child in the single-dose IRV group who had a low but detectable baseline ELISA response; however, this individual had undetectable VNA at day 0 and was an average responder after vaccination by both VNA (peak response 4 IU/mL) and ELISA (peak response 8 AU/mL). Association, estimated with linear regression, of the day 28 anti-rabies virus glycoprotein total IgG concentration and the day 28 anti-rabies VNA concentration for (C) adults and (D) children (day 365 data shown in appendix 2 p 12). Anti-rabies virus glycoprotein IgG1 responses at days 0 and 28 are shown for (E) adults and (F) children in the immunology subset, which included the first 15 participants enrolled in each of the full-dose ChAdOx2 RabG and single-visit IRV vaccine groups; no anti-rabies glycoprotein IgG2 responses were detected in the same samples. Interferon-γ producing T-cell responses measured by ex-vivo ELISPOT in peptide-stimulated PBMCs are shown for (G) adults and (H) children in the immunology subset. ELISPOT responses, shown as SFU per million PBMCs, are the sum of five peptide pools after subtraction of background (media only). IgG ASC were measured by ELISPOT after polyclonal stimulation of frozen PBMCs to stimulate memory B cells and deplete pre-existing plasma cells. Data from day 0 (before vaccination) and nominal day 365 visits from children in the immunology subset are presented as the number of (I) RVG-specific ASC per million cultured PBMCs and (J) RVG-specific ASC as a percentage of the total IgG-secreting ASC. (K–N) ChAdOx2-vector neutralisation exploratory immunology results. Dotted lines represent the limit of detection for the neutralisation assay or VNA assay. (K) Baseline (day 0) anti-vector neutralising antibody titres for adults and children who received a full dose of the ChAdOx2 RabG vaccine. Horizontal lines represent the median for each group, and the dotted line indicates the assay detection limit (ie, negative samples). Anti-vector neutralising antibody kinetics in (L) adults and (M) children. To investigate anti-vector antibody kinetics in participants with a range of pre-vaccination titres, day 28 and nominal day 365 anti-vector antibody titres were measured for five adult participants randomly chosen from each of three strata defined by baseline titre (negative [≤18, green]; moderate [>18 to <200, orange]; and high [≥200, purple]). For children, there were only three participants with a high baseline titre, all of whom were included. Seven children with a moderate baseline titre and five children with a negative baseline titre were randomly chosen. Data on anti-ChAdOx1 vector antibodies are provided in appendix 2 (p 15). (N) Correlation between baseline anti-vector antibody response and day 28 anti-rabies VNA for adults (open circles) and children (closed triangles) for whom both day 0 anti-vector antibody and day 28 VNA data were available (n=52; ie, 24 adults and 28 children). Spearman's rank correlation for pooled data was *r=*–1·663; p=0·2388. Nominal day 365 visits were skewed towards the end of the permissible window (days 273–546), with a median actual visit day of 523 for adults and 432 for children. ASC=antibody-secreting cells. ELISPOT=enzyme-linked immunospot assay. IRV=inactivated rabies virus. PBMC=peripheral blood mononuclear cell. RVG=rabies virus glycoprotein. SFU=spot-forming units. VNA=virus neutralising antibody.

Total rabies glycoprotein-binding IgG ELISA results correlated with, and followed similar temporal and inter-group patterns to, the VNA data; adults and children receiving the single-visit, full-dose ChAdOx2 RabG vaccination showed stronger and better maintained anti-rabies virus glycoprotein antibody responses compared with those who received single-visit or two-visit IRV vaccination (figure 4A–D). Immunoglobulin subclass ELISA demonstrated that responses to both ChAdOx2 RabG and IRV vaccines were skewed towards IgG1, with no antigen-specific IgG2 detected (figure 4E, F). The avidity of anti-rabies virus glycoprotein responses 28 days after vaccination were similar between adults receiving ChAdOx2 RabG and IRV vaccines, whereas children who received single-visit IRV had higher avidity IgG responses than those who received single-visit ChAdOx2 RabG (appendix 2 p 14).

Again, mirroring VNA data, rapid rises in anti-rabies virus glycoprotein IgG were apparent in the small number of participants receiving sPEP (appendix 2 p 13). For most participants within the immunology subset for whom adequate peripheral blood mononuclear cell (PBMC) samples from the day 365 visit were available, cultured memory B-cell ELISPOT responses were detectable (six of seven children who received single-visit IRV and all four children who received full-dose ChAdOx2 RabG; figure 4I, J).

Pre-existing anti-ChAdOx2 RabG vector immunity was detected in both adult and child participants (figure 4K–M). Baseline titres and kinetics over time of anti-vector neutralising antibody in children and adults who received the full dose of ChAdOx2 RabG vaccines are shown in figure 4. Notably, we found no evidence that pre-existing anti-vector neutralising antibody titres affected the immunogenicity of ChAdOx2 RabG: baseline anti-vector neutralising antibody titres did not correlate with day 28 VNA (figure 4N).

Ex-vivo PBMC interferon-γ ELISPOT responses (higher than the individual and population median baseline values) were evident at day 14 in most participants in the immunology subset who received the full-dose ChAdOx2 RabG vaccine (13 of 15 adults and all 14 children), but only a minority of those who received single-visit IRV vaccine (six of 14 adults and six of 15 children; figure 4G, H). Day 14 responses were significantly higher among full-dose ChAdOx2 RabG recipients than IRV recipients (in post-hoc analyses for adults, Hodges–Lehmann estimate of median difference: 431 spot-forming units per million PBMCs [95% CI 63–792]; p=0·0079 by two-tailed Mann–Whitney test; for children, median difference: 161 spot-forming units per million PBMCs [95% CI 91–217]; p<0·0001).

## Discussion

This study has shown an encouraging safety, tolerability, and immunogenicity profile of the ChAdOx2 RabG vaccine, while also providing additional data on the immunogenicity of single-visit and two-visit intradermal regimens for the administration of IRVs in African adults and children. A single intramuscular dose of ChAdOx2 RabG vaccine was more immunogenic than single-visit intradermal vaccination with IRV in both adults and children and the WHO-recommended two-visit IRV PrEP regimen in children.

Duration of protection is a key consideration for PrEP. PrEP recipients are recommended to receive booster PEP after suspected exposure. Such individuals are likely (but not guaranteed) to be protected if their VNA exceeds the CoP either at the time of exposure (ie, independent of the booster) or—if they are able to access the recommended booster—within 7 days. Unfortunately, recommendations are not always followed, particularly in low-resource settings, and therefore we considered both the unboosted maintenance of a VNA of at least 0·5 IU/mL and the recall responses to sPEP after vaccination to be relevant to potential vaccine efficacy. We viewed the attainment of the CoP within 7 days of booster PEP at 1 year as a minimum goal, and long-term booster-independent maintenance of the CoP as a more stringent goal. The long-term maintenance would be most desirable in areas with poor access to PEP. It is therefore particularly encouraging that high proportions of single-visit, full-dose ChAdOx2 RabG recipients (86% of adults and 100% of children) were seropositive at the nominal day 365 visit, with the median time of the 1-year visit having been about 18 months after vaccination in adults and 14 months in children. In considering the possible public health impact in contexts with poor PEP access, it is important to note also that there might have been appreciable booster-independent protection, even after VNA titres waned beneath the CoP (as has been discussed elsewhere).^9^ Ongoing follow-up in the study will provide extended data on antibody kinetics, permitting the modelling of expected durations of maintenance of seropositivity, and allowing a more robust estimation of the proportion of participants with satisfactory recall responses after sPEP.

Larger studies would provide greater precision in estimates of ChAdOx2 RabG immunogenicity, but the data provided by this trial and a previous UK phase 1 study^19^ suggest that the product's immunogenicity is highly likely to suffice for use as single-visit PrEP. In addition to the favourable comparisons to intradermal IRV regimes included in the current trial, the immunogenicity we observed here also compared favourably to historical studies of IRV-based PrEP regimens. In a randomised trial in the Netherlands,^17^ less than 50% of adult travellers had seroconverted 56–63 days after vaccination with single-visit IRV PrEP, and only 25–27% were seropositive 6 months after vaccination. In the same study, a group receiving two-visit intramuscular IRV PrEP had an 86% seroconversion rate 63–70 days after vaccination and 68% were seropositive 6 months later.^17^ Among Belgian adult military personnel who received single-visit IRV PrEP, 82·5% were seropositive 14 days after vaccination, but the majority were seronegative before sPEP 7–28 months after vaccination.^16^ Those who received either two-visit or three-visit IRV PrEP were all seropositive 35 days after vaccination, and the majority were still seropositive 1–3 years later (before sPEP).^15^ Across all three studies, 95–100% per group were seropositive 7 days after receiving sPEP,^15^, ^16^, ^17^ demonstrating the speed of the recall response that could be achieved after the loss of seropositivity, even with regimens achieving less robust initial immunogenicity than observed in the current study.

We are not aware of any other investigational rabies vaccine to have achieved such robust and durable responses with a single-visit regimen. A nucleoside-unmodified mRNA vaccine against rabies failed to induce seroconversion 28 days after single-visit vaccination in a previous trial, but seroconversion was observed after two or more visits.^22^ A self-replicating RNA vaccine has shown more promise, with seroconversion occurring 29 days after single-visit vaccination for most participants receiving higher doses, although most participants were seronegative again by day 85 unless a booster dose was administered at 57 days after vaccination.^23^ Another chimpanzee adenovirus-vectored rabies vaccine candidate (ChAd155-RG), tested in a phase 1 trial in adults in the USA, induced high seroconversion rates only when administered using a two-visit regimen and with a high dose (1 × 10^11^ virus particles) that was double the full dose used in our study (5 × 10^10^ virus particles), with seropositivity falling to less than 30% after 1 year.^24^ Although each study is unique in terms of design and study population, these results contrast with the immunogenicity of ChAdOx2 RabG seen here for Tanzanian adults and children and previously in UK adults.^19^

Some previous studies of adenovirus-vectored vaccines have observed an association between high titres of pre-existing anti-vector neutralising antibody, due to previous exposure to adenovirus infection, or a previous adenovirus-vectored vaccination and weaker antigen-specific immunogenicity.^25^ Here, baseline and post-vaccination anti-ChAdOx2 RabG titres among study participants were similar to those in previous studies of ChAdOx2 RabG (or AdC68, the parental wild-type virus;^26^, ^27^ figure 4K–M).

Adenovirus-vectored vaccines against COVID-19 demonstrated good efficacy, with some evidence of enhanced durability compared with mRNA vaccines,^28^ an effect that could be particularly valuable for rabies PrEP. Use of adenovirus vectors for COVID-19 vaccines also led to the identification of vaccine-induced thrombosis with thrombocytopenia (VITT) as an extremely rare but serious associated adverse reaction.^29^ In populations of European origin, incidence estimates ranging from one case per 26 500 to one case per 261 000 vaccinations have been reported.^29^ VITT is a type of thrombosis with thrombocytopenia syndrome. Although other thrombocytopenia syndromes might be more common among non-European populations, VITT appears less common in such populations.^30^ Research into potential causal mechanisms continues, with most mechanisms independent of the antigen insert (thus applicable to non-COVID-19 vaccines) and some more common in European populations.^30^ Although our understanding of thrombocytopenia syndrome and VITT are incomplete, it is possible that VITT is higher for some adenovirus serotypes than others, and although the ChAdOx2 RabG vaccine uses a different serotype to any of the COVID-19 vaccines, it is not possible for any phase 1 or 2 study to rule out rare side-effects.

Wider use of PrEP in rabies-endemic areas is currently limited by doubt regarding cost-effectiveness and programmatic feasibility.^3^, ^9^ Both issues could be addressed by lower cost, abbreviated regimens and by integrating these within existing childhood vaccination visits.^9^ Although intradermal IRV immunogenicity in our study was lower than that of ChAdOx2 RabG, the responses obtained were nonetheless similar to those seen in IRV studies in predominantly European and Asian populations.^15^, ^16^, ^17^ Our data thus encourage the wider use of IRV PrEP in African populations. There is a need for co-administration studies to show non-interference with existing vaccines that form the core of routine vaccination schedules.

Our study also raises the possibility of abbreviating rabies PEP for those who have not received PrEP. Because our design focused primarily on PrEP, it did not include comparator groups receiving a WHO-recommended PEP regimen (eg, a day 0, 3, and 7 three-visit regimen). The immunogenicity of ChAdOx2 RabG at early timepoints (days 7–14 after vaccination), and its improvement compared with a two-visit (day 0 and 7) IRV regimen in children, nonetheless encourage the further exploration of ChAdOx2 RabG for PEP. We speculate that the co-administration of ChAdOx2 RabG with an IRV vaccine at a single visit might provide even quicker and more consistent seroconversion than we observed with ChAdOx2 RabG alone (with the IRV vaccine providing immediate antigen availability to complement the sustained by delayed antigen expression seen after adenovirus-vectored vaccination).^28^ We are now planning a further trial comparing such single-visit co-administration with a three-visit IRV PEP regimen (NCT07168018). A reliable single-visit PEP regimen would transform current bite management, lower direct and societal costs of PEP, and enhance access and equity for bite recipients.

A limitation of the current report is that few participants, and only those who received single-visit IRV as their primary vaccination, have been randomly assigned to receive sPEP to date. This largely reflects the good early immunogenicity of all regimens, resulting in few participants having a VNA of less than 0·5 IU/mL and hence being eligible for allocation to sPEP at day 365. The rapid recall responses, measured by VNA and anti-rabies virus glycoprotein IgG ELISA, seen in the few sPEP recipients, plus memory B-cell responses seen in the immunology subset, strengthen confidence that both ChAdOx2 RabG and single-visit IRV vaccination induce long-lived immunological memory, and hence will achieve prompt recall upon later booster PEP or exposure. At the end of follow-up, by which time all participants will receive sPEP, secondary immunological analyses of long-term VNA maintenance and sPEP responses will be reported. Another limitation is that sex was self-reported.

With tens of thousands of rabies deaths annually,^1^ there is a clear need for new approaches to rabies vaccination.^3^, ^9^ Tailored approaches might be needed for different contexts, with acceptance that PrEP might have a role in contexts where human rabies elimination by PEP and dog vaccination is highly uncertain in the near term.^9^ Our results show that ChAdOx2 RabG is well tolerated and highly immunogenic. Given the low cost of manufacturing adenovirus-vectored vaccines (we previously estimated less than US$1 per dose for bulk vaccine),^20^ the suitability of the platform for thermostabilisation,^31^ and ChAdOx2 RabG's robust induction of VNA exceeding the correlate of protection, ChAdOx2 RabG could offer a new route to control the burden of human rabies in low-resource settings.

### Contributors

### Equitable Partnership Declaration

### Data sharing

The study protocol, statistical analysis plan, supplementary data, figures, and tables are provided in appendix 2. After publication, de-identified participant data will be made available on direct request to the corresponding author. Proposals will be reviewed by the sponsor, corresponding author, and collaborators on the basis of scientific merit. Data will be shared after approval of the proposal and signed data-access agreements.

## Declaration of interests

AJR reports royalties arising from the University of Oxford-AstraZeneca COVID-19 vaccine, which also uses the chimpanzee adenovirus technology platform, and potential income arising from the licensing of intellectual property related to the ChAdOx2 RabG vaccine. AB reports royalties arising from the University of Oxford-AstraZeneca COVID-19 vaccine. ADD reports grant funding from the Medical Research Council (funding number MR/P017339) and the Engineering and Physical Sciences Research Council (funding numbers EP/X038181 and EP/Y530542); royalties arising from the University of Oxford-AstraZeneca COVID-19 vaccine; potential income arising from the licensing of intellectual property related to the ChAdOx2 RabG vaccine or other adenovirus-vectored vaccines; receipt of consultancy fees from AstraZeneca related to another adenovirus-vectored vaccine; and being a named inventor on patent applications relating to chimpanzee adenovirus platform technology (application numbers WO/2017/221031, WO/2022/123030, and WO/2022/123033). All other authors declare no competing interests.

## Supplementary Materials

Swahili translation of the abstract

Supplementary appendix 2

Equitable Partnership Declaration
