# Supplementary material for: Safety and immunogenicity of a single-dose adenovirus-vectored rabies vaccine over 1 year in adults and children in Tanzania: interim data from an ongoing, partly randomised, controlled, phase 1b/2 trial
Source: Lancet Infect Dis. 2026 Aug;26(8):843–57. doi: 10.1016/S1473-3099(26)00071-X (PMC13391811; doi:10.1016/S1473-3099(26)00071-X)
Supplement: Swahili translation of the abstract [file mmc1.pdf]

THE LANCET
Infectious Diseases

Supplementary appendix 1

This translation in Swahili was submitted by the authors and we reproduce it as supplied. It has not been peer reviewed. *The Lancet*'s editorial processes have only been applied to the original in English, which should serve as reference for this manuscript.

Tafsiri hii katika Swahili iliwasilishwa na waandishi na tunatengeneza tena kama hutolewa. Haijapitiwa. Mchakato wa hariri wa Lancet Global Health umetumika tu kwa asili kwa Kiingereza, ambayo inapaswa kutumika kama kumbukumbu kwa muswada hii.

Supplement to: Ritchie AJ, Hassan O, Urasa N, et al. Safety and immunogenicity of a single-dose adenovirus-vectored rabies vaccine over 1 year in adults and children in Tanzania: interim data from an ongoing, partly randomised, controlled, phase 1b/2 trial. *Lancet Infect Dis* 2026; published online April 28. https://doi.org/10.1016/S1473-3099(26)00071-X.

Usalama na uwezo wa kuchochea kinga mwili kwa kipindi cha zaidi ya mwaka mmoja ,wa chanjo ya kichaa cha mbwaa inayowasilishwa na kirusi kibebeshi aina ya adenovirus kwa watu wazima na watoto wa kitanzania: taarifa ya muda mfupi kutoka kwenye utafiti unaoendelea wa kinasibu kwa vipande, uliodhibitiwa wa awamu ya 1b/2.

UTANGULIZI: Kichaa cha mbwa kinaua takribani watu 59000 kila mwaka. ChAdOx2 RabG ni chanjo tarajiwa ya kichaa cha mbwa inayowasilishwa kupitia kirusi kibebeshi cha aina ya Adenovirus cha jamii ya nyani na tumbili.Chanjo hii ina uwezo kutoa kinga ya kabla ya kukutana na maambukizi ya kichaa cha mbwa kwa dozi moja na gharama ndogo.Tulilenga kutathmini usalama na uwezo wa kuchochoea kingi wa chanjo tarajiwa ya ChAdOx2 RabG, ikiwemo kuilinganisha na chanjo linganishi iliokua na kibali.

MBINU Tulifanya utafiti wa awamu ya 1b/2 uliokua na lebo ya wazi, katika kituo kimoja wa kushuka umri na kupandisha dozi na ulionasibiwa baadhi ya sehemu.Watu wazima wenye afya njema (Umri wa miaka 18-45) na Watoto wenye afya njema (Umri wa miaka 2-6) ambao hawakuwahi kupata chanjo ya kichaa cha mbwa na wanaoishi maeneo ya Bagamoyo mjini walijumuishwa katika utafiti kwa kupitia taratibu zilizohusiaha mlolongo wa mikutano na jamii. Watu wazima walitangulia kuanza utafiti kwa kupangwa (bila unasibu) katika Kundi lililopkea dozi ya chembe za virusi 2.5×10^{10} vya ChAdOx2 RabG (dozi ya Katikati), baada ya uhakiki wa usalama katika Kundi hilo, watu wazima wengine walipangwa kinasibu (kwa uwiano wa 3:1) kupokea ChAdOx2 RabG (chembe za virusi 5×10^{10}; dozi kamili) au chanjo ya kichaa cha mbwa (yenye kibali) ilio na virusi vya kichaa cha mbwa vilivyo uliwa/kuzimwa. Watoto walianza ushiriki kwa baadhi yao kupangwa (bila unasibu) katika Kundi lililopokea chembe za virusi 1×10^{10} vya ChAdOx2 RabG (dozi ndogo) na baadae, wengine kupangwa katika Kundi liliopokea dozi ya katikati (2.5×10^{10} vya ChAdOx2 RabG). Baada ya uhakiki wa usalama kwa washiriki wa makundi haya, Watoto waliofuata walipangwa kinasibu (kwa uwiano wa 3:2:2) kupokea aidha dozi kamili ya ChAdOx2 RabG siku ya kwanza, chanjo ya virusi vya kichaa cha mbwa vilivyo uliwa/kuzimwa katika udhurio moja(siku ya awali) au chanjo ya virusi vya kichaa cha mbwa vilivyo uliwa/kuzimwa katika maudhurio mawili (siku ya awali na ya 7). ChAdOx2 RabG ilitolewa kwa njia ya sindano ya msuli katika sehem moja ya mwili na chanjo ya virusi vya kichaa cha mbwa vilivyo uliwa/kuzimwa ilitolewa kwa sindano ya Katikati ya ngozi kwenye sehemu mbili tofauti za mwili.Unasibishwaji ulifanyika kwa hatua na timu ya utafiti kwa kutukia orodha ilioandaliwa na mwanatakwimu huru. Tokeo la msingi la uchunguzi lilikua usalama, uliotathminiwa katika kundi la washiriki wote waliopangwa kupokea bidhaa ya utafiti au kilinganishi. Tokeo la kisekondari lilikua ni kingamwili inayo dhibiti virusi vya kichaa cha mbwa, iliotathminiwa na kipimo kilichodhibitishwa katika washirika kwenye kundi la chanjo ya virusi vya kichaa cha mbwa vilivyo uliwa/kuzimwa iliotolewa kwenye udhurio moja, makundi yaliopokea dozi kamili ya ChAdOx2 RabG katika udhurio moja walioweza kukamilisha udhurio la mwaka moja toka kupokea chanjo. Ufuatiliaji wa washiriki umepangwa kwendelea mpaka kufikia mwiaka mitano na nusu baada ya chanj ya awali.

Tunawasilisha taarifa za mwaka wa kwanza wa kipindi hiko. Utafiti huu umesajiliwa ClinicalTrials.gov (NCT04270838)

MATOKEO : Baina ya Machi 3,2022 na Machi 9,2023, watu wazima 63 (watatu katika kundi la dozi ya Katikati ya ChAdOx2 RabG, 45 katika Kundi la dozi kamili ya ChAdOx2 RabG na 15 katika Kundi la chanjo ya virusi vya kichaa cha mbwa vilivyo uliwa/kuzimwa la udhurio moja) na watoto 111 (watatu katika makundi ya dozi ndogo na dozi ya Katikati ya ChAdOx2 RabG,45 katika Kundi la dozi kamili ya ChAdOx2 RabG,30 katika Kundi la chanjo ya virusi vya kichaa cha mbwa vilivyo uliwa/kuzimwa kwa udhurio moja na 30 katika Kundi la chanjo ya virusi vya kichaa cha mbwa vilivyo uliwa/kuzimwa kwa maudhurio mawili) waliingia katika ushiriki wa utafiti. Washiriki waliripoti dalili ya athari za chanjo ambazo kwa kiasi kikubwa zilikua ni za ukali modogo au wa wastani, dalili zilizoripotiwa kwa wingi zilikua ni maumivu eneo la kuchomwa sindano ya chanjo au kuhisi homa, na hakukua na athari za hatari. Katika washiriki watu wazima,kiwango cha wastani wa kijiometria wa kingamwili inayo dhibiti virusi vya kichaa cha mbwa siku ya 365 baada ya chanjo ya kwanza zilikua ni 2.0 (uhakika wa asilimia 95, 1.4-2.9)baada ya chanjo ya dozi kamili ya ChAdOx2 RabG na 0.4 (0.2-0.7) baada ya chanjo ya virusi vya kichaa cha mbwa vilivyo uliwa/kuzimwa kwa udhurio moja (wastani wa kijiometria wa uwiano wa 5.1[uhakika wa asilimia 95, 2.5-10.4};p<0.0001).Katika makundi ya Watoto ,wastani wa kijiometria wa kiwango cha kingamwili kilicho dhibiti virusi vya kichaa cha mbwa siku ya 365 baada ya chanjo ilikua 6.1 (4.5-8.2) baada ya chanjo ya udhurio moja ya dozi kamili ya ChAdOx2 RabG na 0.7 (0.5-1.1) baada ya chanjo ya virusi vya kichaa cha mbwa vilivyo uliwa/kuzimwa, kwa udhurio moja (uwiano wa kijiometria wa 8.6 [5.4-13.9];p<0.0001)Katika uchambuzi uliokubalika kufanyika baada ya matokeo , kiwango cha kingamwili kilicho dhibiti virusi vya kichaa cha mbwa katika Kundi la Watoto lililopokea dozi kamili ya ChAdOx2 RabG kilizidi kiwango kutoka kwenye Kundi la Watoto lililopokea chanjo ya virusi vya kichaa cha mbwa vilivyo uliwa/kuzimwa kwa maudhurio mawili. (wastani wa kijiometria 3.0 [uhakika wa asilimia 95, 2.2-4.1];uwiano wa wastani wa kijiometria wa 2.0[1.3-3.1];p=0.0028).

TAFSIRI : Chanjo tarajiwa ya ChAdOx2 RabG imeonekana kua salama na kuvumilika na watu wazima Pamoja na watoto wa kitanzania. Chanjo imeonekana kuchochea kiwango madhubuti na cha kudumu cha kingamwili kinacho dhibiti virusi vya kichaa cha mbwa, huku kiwango cha kinga siku 365 baada ya chanjo kikiwa kimezidi kiwango kinachowiana na ulinzi dhidi ya ugonjwa wa kichaa cha mbwa (kiwango kingamwili kinacho dhibiti virusi vya kichaa cha mbwa ≥0.5 IU/mL) na kiwango katika makundi yaliopokea chanjo linganishi ya kichaa cha mbwa. ChAdOx2 RabG inaweza kua chaguo la gharama ndogo na rahisi kwa ajili ya kutoa kinga ya kabla ya kukutana na maambukizi ya kichaa cha mbwa kwa watu wanaoishi maeneo ambayo ugonjwa wa kichaa cha mbwa umeenea na hivyo kuwezesha utumiaji wa chanjo kabla ya kukutana maambukizi ya ugonjwa huo kua makakati wenye tija kulingana na gharama yake katika kuzuia ugonjwa wa kichaa cha mbwa
