# Supplementary material for: Safety and immunogenicity of a single-dose adenovirus-vectored rabies vaccine over 1 year in adults and children in Tanzania: interim data from an ongoing, partly randomised, controlled, phase 1b/2 trial
Source: Lancet Infect Dis. 2026 Aug;26(8):843–57. doi: 10.1016/S1473-3099(26)00071-X (PMC13391811; doi:10.1016/S1473-3099(26)00071-X)
Supplement: Supplementary appendix 2 [file mmc2.pdf]

THE LANCET
Infectious Diseases

Supplementary appendix 2

This appendix formed part of the original submission and has been peer reviewed. We post it as supplied by the authors.

Supplement to: Ritchie AJ, Hassan O, Urasa N, et al. Safety and immunogenicity of a single-dose adenovirus-vectored rabies vaccine over 1 year in adults and children in Tanzania: interim data from an ongoing, partly randomised, controlled, phase 1b/2 trial. *Lancet Infect Dis* 2026; published online April 28. https://doi.org/10.1016/S1473-3099(26)00071-X.

Appendix Contents

Supplementary methods 2
- Randomisation processes 2
- Pre-specified analyses of virus neutralising antibody (VNA) titres 2
- Higher-sensitivity virus neutralising antibody assay 3
- Enzyme-linked immunosorbent assays (ELISAs) 3
- ELISpot Assay for detection of memory B cells 4
- T cell ELISpot 5
- Anti-vector neutralising antibody 5

Supplementary figures 7
- Supplementary figure 1: Detailed participant flow 7
- Supplementary figure 2. Solicited adverse events, including dose-escalation groups. 9
- Supplementary figure 3. Virus neutralising antibody (VNA) levels for females and males. 10
- Supplementary figure 4. Duration of maintenance of VNA ≥0.5 IU/mL. 11
- Supplementary figure 5. Relationship of VNA and total IgG ELISA results. 12
- Supplementary figure 6. Total anti-RVG IgG ELISA results following sPEP. 13
- Supplementary figure 7. Avidity of anti-RVG IgG responses 28 days post-vaccination. 14
- Supplementary figure 8. Anti-ChAdOx1 vector neutralising antibody titres. 15

Supplementary tables 16
- Supplementary table 1. Deviations from the protocol. 16
- Supplementary table 2. Serious adverse events 17
- Supplementary table 3. Local and systemic adverse events in adult groups, broken down by participant sex. 18
- Supplementary table 4. Local and systemic adverse events in paediatric groups, broken down by participant sex. 19
- Supplementary table 5. Unsolicited adverse events (AEs) in adult participants. 20
- Supplementary table 6. Unsolicited adverse events (AEs) in paediatric participants. 21
- Supplementary table 7: Laboratory abnormalities in adult participants. 22
- Supplementary table 8: Laboratory abnormalities in child participants. 23
- Supplementary table 9. Ranges used to grade laboratory abnormalities. 24
- Supplementary table 10. Summary of VNA data for adult groups from day 28. 25
- Supplementary table 11. Summary of VNA data for paediatric groups from day 28. 26
- Supplementary table 12. VNA comparisons between groups at multiple timepoints 27
- Supplementary table 13: Calculation of likely boosted protection 29

Supplementary author list - the RAB002 study team 31

Supplementary references 31

RAB002 trial protocol 32

RAB002 statistical analysis plan 121

Supplementary methods

Randomisation processes

Adults were randomised to full-dose ChAdOx2 RabG (groups AC2/3) or single-visit IRV (AV1) at a ratio of 3:1 and children to full-dose ChAdOx2 RabG, single-visit IRV or two-visit IRV (groups PC2/3, PV1 or PV2 respectively) at a ratio of 3:2:2. Adults were enrolled first, with paediatric groups enrolled following an interim safety review by the safety monitoring committee. Eligible participants were assigned a study identity code, which was linked to a random number code on the list provided to clinical staff. Treatment allocation was revealed to clinical staff and participants only immediately prior to vaccination: code numbers were linked to treatment allocation in the list provided to pharmacy staff (who had no contact with participants), who documented the allocation in the randomisation case report form and issued the vaccine.

For participants with most recent VNA <0.5 IU/mL prior to the day 365 visit, randomisation to timing of sPEP was stratified by group and membership of the immunology subset, in balanced blocks of two (if ≤2 participants in a stratum were to be randomised at the visit) or four, with allocations applied from the randomisation list to the list of eligible participants by the statistician.

Clinical staff and participants were not masked either to treatment group allocation or to allocation of timing of sPEP.

Pre-specified analyses of virus neutralising antibody (VNA) titres

Within the overall secondary trial objective of characterising immunogenicity, a hierarchy of immunological outcome measures and hypothesis tests were pre-specified in the statistical analysis plan (supplementary material).

Co-primary immunological outcome measures were chosen to reflect maintenance at the nominal day 365 visit of 'booster-independent protection' (unboosted VNA titre) and the proportion of participants expected to be protected if able to access PEP after an exposure ('boosted protection', i.e. VNA ≥0.5 either without sPEP or within 7 days after sPEP).

Unboosted VNA titre at the day 365 visit was subject to the primary pre-specified hypothesis test: a two-tailed unpaired test comparing recipients of a single, full dose of ChAdOx2 RabG vaccine and recipients of a single-visit Verorab, performed separately in adults and children, with the choice between a t-test and a Mann-Whitney test to be determined by testing of departure from normality (d'Agostino-Pearson test). Prior to t-tests, although not pre-specified, we also performed a test for homogeneity of variance (F-test), and selected Student's or Welch's t-test accordingly.

Treatment of negative VNA results was not pre-specified: for calculation of descriptive statistics and hypothesis tests, we assigned such values an arbitrary value equal to the lower limit of quantification of the assay (0.22 IU/mL). This will tend to reduce differences between more and less immunogenic regimes, and hence be conservative with respect to estimates of difference and tests for difference.

Although pre-specified to be reported as a co-primary immunological outcome measure, 'boosted protection' was not subject to a hypothesis test, partly because we expected it to be similarly high (close to 100%) in all groups. 'Boosted protection' was estimated as the sum of those with 'booster-independent protection' (i.e. VNA ≥0.5 at day 365) and those with 'booster-PEP-dependent' protection (VNA<0.5 at day 365, but ≥0.5 at sPEP+7). Data was imputed for three subsets of participants for whom the required data was not available:

1. Individuals lost to follow-up who were seropositive (VNA ≥0.5) at their latest visit were excluded from the calculation.
2. Individuals with VNA<0.5 at the timepoint used to determine eligibility for sPEP randomisation (day 56) but allocated to sPEP at the end of the study (rather than day 365) were imputed to

have the same probability of VNA≥0.5 at sPEP+7 as that observed in participants from the same group undergoing sPEP at day 365. Imputation for such participants if no individual in that group had been allocated to day 365 sPEP was not considered in the SAP, and is discussed in the results section.

3. Individuals with VNA≥0.5 at day 56 but <0.5 at day 365 (i.e. not known at the day 365 visit to be seronegative and hence not randomised for sPEP) were also imputed to have the same probability of VNA≥0.5 at sPEP+7 as those from the same group who did undergo sPEP.

Higher-sensitivity virus neutralising antibody assay

To measure the speed of seroconversion after vaccination with ChAdOx2 RabG, virus neutralising antibody (VNA) was measured with an assay using less dilute samples, and thus with greater sensitivity, than that used at other timepoints. The fluorescent antibody virus neutralisation (FAVN) test, following the method outlined in the World Organisation for Animal Health (WOAH) terrestrial manual,[1] was used. Day 0, 7 and 14 samples from the adult and paediatric participants who received a high-dose of ChAdOx2 RabG and were assigned to the ‘immunology subset' were analysed. The assay had a lower limit of detection of 0.03 IU/mL (as compared to the limit of 0.22 IU/mL with the assay used for other analyses).

Enzyme-linked immunosorbent assays (ELISAs)

Rabies virus glycoprotein (RVG) was expressed as previously described.[2] Quantification of total rabies glycoprotein-binding IgG was performed using the method and positive reference sample as described previously,[2] with the exception that plates were coated with 1µg/mL of RVG. Assays were carried out on samples from all participants at all available timepoints.

Previous studies of IRV responses have found VNA and ELISA responses to be closely related, and it has been suggested that ELISA might, in some circumstances, serve as a surrogate of VNA. We therefore explored the relationship of VNA and ELISA at day 28 in adults and in children by performing linear regression, separately for age group after log-transformation of both variables. For both age groups, residuals passed tests for normality (d'Agostino Pearson) and homoscedasticity (Spearman rank correlation of absolute values of residuals with predicted Y values).

IgG subclass ELISAs were performed to quantify anti-rabies glycoprotein IgG1 and IgG2 responses in the day 28 samples from adult and paediatric participants from the ‘immunology subset'. Nunc MaxiSorp flat-bottom ELISA plates (44-2404-21, Thermo) were coated overnight at 4 °C with 2 µg/mL of purified RVG in phosphate-buffered saline (PBS). To produce standard curves, some wells on each plate were coated with IgG1 (HCA049A, Bio-rad) or IgG2 (HCA108 Bio-rad) using doubling dilutions, with concentration ranging from 25 – 0.39 µg/mL, such that each plate was coated with both RVG and the IgG1 and IgG2 standards, allowing for both standard curves and detection of cross-reactivity. Following the overnight incubation, plates were washed 6 times with 0.05% PBS-Tween 20 (PBS-T, P1379, Sigma-Aldrich) and blocked with 100 µL/well of 10% (w/v) skimmed milk (70166, Merck Life Science UK Limited) in PBS-T for 1 hour at room temperature (RT). Test samples were then diluted in 1% skimmed milk in PBS-T, with dilutions ranging from 1:50 to 1:600. Plates were washed 6 times with PBS-T, and 50 µL/well of each diluted sample was added to the plates in duplicate. For IgG1 and IgG2 coated standard curve wells 50 µL/well of 1% skimmed milk in PBS-T added. Plates were then incubated for 2 hours at room temperature and then washed with PBS-T. Detection was carried out using isotype-specific secondary antibodies.

For IgG1 detection, 50 µL/well of mouse anti-human IgG1 Fc–alkaline phosphatase (AP) conjugate (cat. no. 9054-04, Southern Biotech), diluted 1:1000 in 1% skimmed milk in PBS-T, was added. For IgG2 detection, 50 µL/well of biotinylated anti-human IgG2 antibody (cat. no. 05-3540 Thermofischer) diluted 1:500, was added. Following a 1-hour incubation at room temperature, plates were washed 6 times with PBS-T and incubated with 50 µL/well of ExtrAvidin-AP (E236- Merck Life Science UK Limited)

(diluted 1:5000 in PBS-T) for 30 minutes at room temperature. Plates were then washed 6 times with PBS-T and developed using 100 µL/well p-nitrophenyl phosphate (pNPP) alkaline phosphatase substrate (N2765, Sigma-Aldrich) for 1-3 hours at room temperature. Absorbance was measured at 405 nm using an ELx808 absorbance reader (BioTek). Prism software (GraphPad, version 10.4.0) was used to interpolate sample optical density (OD405) values from the standard curve, which was fitted using a four-parameter logistic model to calculate antibody concentrations. Samples with OD_{405} values below the linear range of the standard curve at the lowest dilution tested were assigned a minimum concentration corresponding to the assay's lower limit of quantification.

To assess avidity of anti-rabies IgG antibodies from the day 28 samples from the 'immunology subset', Nunc MaxiSorp flat-bottom ELISA plates were coated with 2 µg/mL of RVG in PBS and incubated overnight at 4 °C. Plates were washed 6 times with PBS-T and blocked with 100 µL/well of 10% (w/v) skimmed milk in PBS-T for 1 hour at room temperature. To control for variation in antibody titre, day 28 serum samples were diluted in 1% skimmed milk in PBS-T using a dilution factor based on concentrations of anti-rabies total IgG measured previously by ELISA. Plates were then incubated with the diluted serum for 2 hours at RT, followed by 6 washes with PBS-T. A doubling dilution series of sodium thiocyanate (80518, Merck Life Science UK Limited) (NaSCN, 4-0.6M) was then prepared, and 50 µL/well of each dilution or a control containing no NaSCN was added in duplicate and incubated for 15 minutes at RT. Plates were washed 6 times with PBS-T followed by IgG detection using a goat anti-human IgG (γ-chain specific) alkaline phosphatase conjugate (1:1000) (cat no. A3187 Sigma-Aldrich). This was followed by a final 6 times wash step in PBS-T, and the plates were developed with the addition of 100 µL/well of pNPP substrate. Absorbance was read continuously at 405 nm until wells without NaSCN reached an OD of approximately 1.0.

Avidity (%) was then calculated (OD_{405} with NaSCN / OD_{405} without NaSCN) × 100. Avidity percentages were then plotted against $\log_{10}$transformed NaSCN concentrations, and non-linear regression was performed in Prism using the log(inhibitor) vs. normalised response model to generate sigmoidal dose response curves. The half-maximal inhibitory concentration (IC_{50}) was calculated for each sample.

ELISpot Assay for detection of memory B cells

ELISpot based quantification of RVG-specific memory B cells through polyclonal expansion and differentiation of memory B cells into antibody secreting cells (ASC) and depletion of pre-existing plasma cells was performed as previously described.[3,4] Frozen peripheral blood mononuclear cells (PBMCs) from the paediatric immunology subset were thawed and resuspended in complete culture medium (CM) consisting of RPMI 1640 (Sigma, R-5886) supplemented with 10% heat-inactivated, batch-tested fetal bovine serum (FBS; Sigma, F9665), Penicillin-Streptomycin (Sigma, P-4458), L-Glutamine (Sigma, G-7513), sodium pyruvate (Life Technologies, 1140035), and β-mercaptoethanol (Life Technologies, 31350010). PBMCs were then washed, and both cell viability and concentration determined using an automated cell counter (Countess II FL Invitrogen). Cells were then cultured in 96-well round-bottom culture plates (Greiner 650180) at a final concentration of 2×10^6 cells/mL, in the presence of the following polyclonal stimulants: Staphylococcus aureus Cowan strain (SAC; Pansorbin cell suspension, VWR, international, 507861) at a 1:5000 dilution, pokeweed mitogen (Sigma-Aldrich cat no. L-9379) at 1:6000 dilution, and CpG oligonucleotide ODN-2006 (TCG-TCG-TTT-TGT-CGT-TTT-GTC-GTT; Invitrogen, tlrl-2006-5) at a 1:58.8 dilution. Cultured cells were then incubated for six days at 37 °C with 5% CO_2 and 95% humidity to promote the differentiation of resting memory B cells into antibody-secreting cells (ASCs) before ELISpot analysis. Following stimulation, PBMCs were harvested, washed with AutoMACS rinsing solution (Miltenyi Biotec, 130-091-222), and counted using an automated cell counter.

The enumeration of antigen-specific and total IgG+ ASCs was performed using ELISpot. 96-well PVDF ELISpot plates (Millipore, MAIPS4510) were pre-activated with 35% ethanol, followed by five washes with sterile water. Wells were then coated overnight at 4 °C with 100ul/ well with one of the following:

purified Rabies virus glycoprotein (RVG) at 2.5 µg/mL, Anti-human IgG (Goat anti-human IgG-UNLB, Southern Biotech, 2047-01) at 20 µg/mL, a cocktail of diphtheria (Statens Serum institute, 2657) at 10 µg/mL and tetanus toxoid (Statens Serum institute, 2674 at 5 µg/mL, or PBS alone (negative control). Plates were then subsequently washed three times with 200 µL/well of sterile PBS and blocked with 200 µL/well of CM for at least one hour at 37 °C in 5% CO_2 and 95% humidity.

Cultured PBMCs were then resuspended at 2×10^6 cells/mL in CM, and 100 µL/well (2×10^5 cells) were added to wells coated with RVG (in triplicate) and diphtheria /tetanus toxoid (in duplicate or triplicate, depending on the number of cells available). For total IgG+ ASC enumeration, 100 µL/well cells were added undiluted, 1:15 diluted and 1:225 diluted to wells coated with anti-human IgG, in triplicate or duplicate depending on cell availability. This was then followed by overnight incubation at 37 °C with 5% CO_2 and 95% humidity for 16 hrs. After incubation, cells were discarded by flicking, and plates were tapped onto tissue paper. Plates were then washed four times with PBS containing 0.05% Tween-20 (PBS-T, P1379, Sigma-Aldrich) and a final wash with PBS alone. Detection was then done using goat anti-human IgG conjugated to alkaline phosphatase (AP) (Merk Life Science UK Ltd, 401442) diluted 1:5000 in CM, and added to each well at 50 µL/well. Plates were then incubated at room temperature for 4 hours, followed by 4 washes with PBS-T, a final 3 washes with distilled water.

To develop the plates, substrate solution (Bio-Rad AP, 170-6432) was freshly prepared, and 50 µL/well added. Spot development was monitored and stopped with 200 µL/well of water upon the appearance of distinct spots, while avoiding excessive background colouration. This was then followed by 3 washes with distilled water and dried overnight. The plates were then read using pre-defined counting parameters on an AID ELISpot reader and software (v7.0). Plates were then manually checked for debris and background artefacts, which were removed before data export to Excel. Statistical analyses were then performed using GraphPad Prism v10.

T cell ELISpot

To assess T-cell responses, ex vivo IFN-γ ELISpots were performed, as previously described,[2] on fresh peripheral blood for participants from the 'immunology subset' at days 0 and 14. 2.5×10^5 PBMCs/well were stimulated using five pools of ten 20-mer rabies glycoprotein peptides with 10-mer overlaps (Mimotopes UK Ltd) in culture media, culture media alone (negative control) or anti-human CD3 antibodies (positive control; Tonbo Biosciences). All pools and controls were tested in triplicate, and the lower limit of detection for the ELISpot assay was 20 spot-forming units (sfu) per million PBMCs.

Anti-vector neutralising antibody

Anti-ChAdOx2 vector neutralising antibody titres (AVA) were measured for full-dose ChAdOx2 RabG recipients, using a previously-described assay.[5]

In brief, reporter-expressing ChAdOx2 or ChAdOx1 SEAP virus was incubated with a dilution series of heat-inactivated serum (final dilutions of 1:18, 1:72, 1:288, 1:1152 and 1:4608) for 1 hour at 37°C. This was then added to GripTite 293 cells (Invitrogen R795-07) seeded in a 96 well plate at 3×10^4 cells per well for 1 hour at 37°C before being removed and replaced with fresh medium. After 24 hours supernatants were assessed for concentration of SEAP using the Phospha-Light SEAP reporter gene assay system (Thermo Fisher T1017). Each plate included negative and positive controls for neutralisation (respectively, ChAdOx2 SEAP without serum, and with a pool of day 28 post-vaccination serum known to be strongly neutralising).

The antibody titre was calculated as the reciprocal of the dilution of the day 0 serum required to reduce the expression level of SEAP by 50% of the level seen without serum. Any sample failing to reduce the level by at least 50% at the first dilution in the series (1 in 18) was considered negative for neutralisation.

All day 0 samples were run in the ChAdOx2 AVA assay, and a selection of 30 participants (15 adult, 15 paediatric) chosen for further analysis; participants were chosen to give a mix of negative, moderate and strong AVA responses to ChAdOx2 at day 0. Samples from these participants were assayed for ChAdOx2 AVA at days 28 and the nominal 1 year visit, and for ChAdOx1 AVA at days 0, 28 and the nominal 1 year visit.

Spearman rank correlation analysis was performed to examine for any relationship between day 0 ChAdOx2 AVA and day 28 VNA. Non-parametric analysis was selected on the basis that, if any relationship exists, it is likely to be non-linear (e.g. apparent only above a certain level of anti-vector antibody titre), and the data set contained several samples negative for AVA.

Supplementary figures

Supplementary figure 1: Detailed participant flow

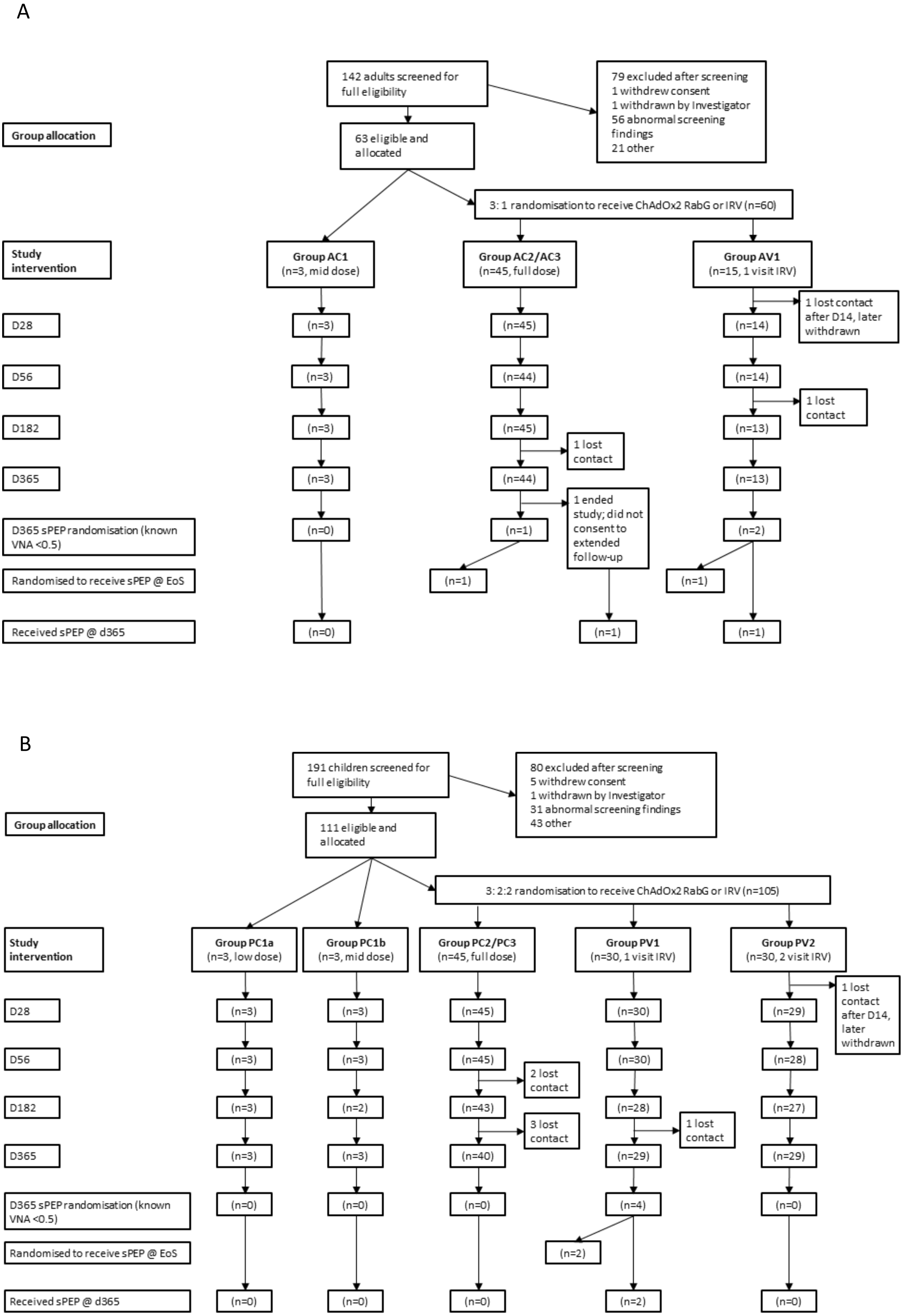

Supplementary figure 1. Detailed participant flow.

CONSORT flow diagrams for adults (A) and children (B), illustrating the study design and participant recruitment. Safety follow-up was conducted for all enrolled participants and at all timepoints. Unless “withdrawn” or “lost contact” is shown, fluctuation in n at individual timepoints reflects occasional volunteers missing individual visits. EoS – end of study. Numbers shown include one child who attended the day 56 visit 1 day outside the permitted visit window, twelve adults who attended the day 365 visit 1-4 days outside the permitted window for day 365 without having lost contact with the study team, and one child in group PV1 who was temporarily lost to follow-up, and attended the day 365 visit 70 days outside the permitted window, and was excluded from immunogenicity analyses (see supplementary table 13). The investigators agreed prospectively (before data analysis) that data from the visits 1-4 days outside the window should be considered as part of immunogenicity analyses for the D365 visit, while data from the group PV1 participant temporarily lost to follow-up should not be. Day 365 visits were skewed towards the end of the permissible window (days 273-546), with a median visit day of 523 for adults and 432 for children.

Supplementary figure 2. Solicited adverse events, including dose-escalation groups.

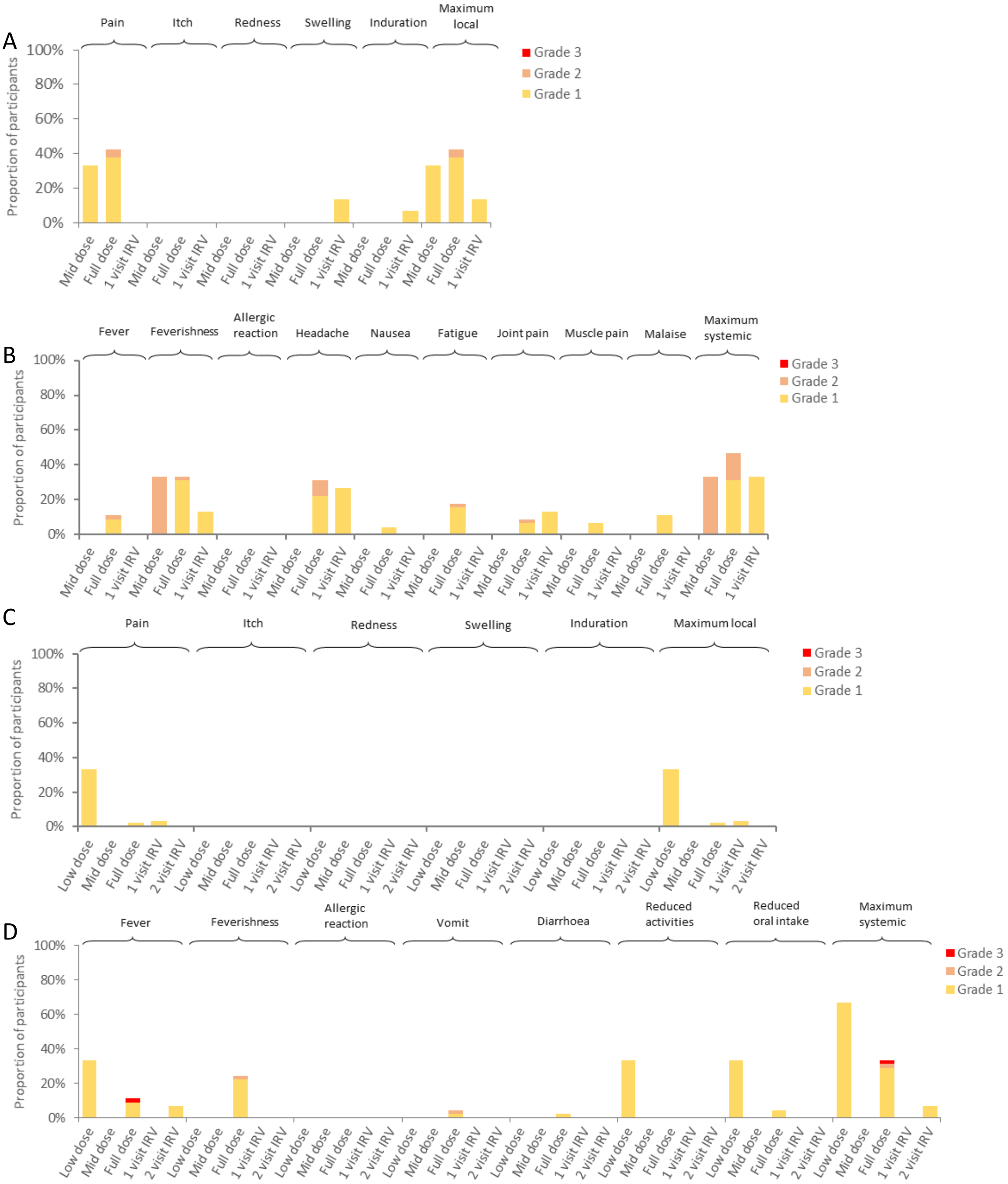

Supplementary figure 2. Solicited adverse events, including dose-escalation groups.

Local (A, C) and systemic (B, D) adverse events in adult (A, B) and paediatric (C, D) participants. This figure differs from Figure 2 in that it shows all groups (including low-dose and mid-dose ChAdOx2 RabG lead-in groups). All data is stratified according to primary vaccination group (low-, mid- and full-dose ChAdOx2 RabG, single-visit IRV, two-visit IRV). For each of the solicited events, the percentage of participants in each group reporting the adverse event in the 7 days following the day 0 vaccination is shown, reflecting the maximum severity reported for each participant during that period. In addition, to provide a global view of reactogenicity, the highest graded of all local and systemic reactions is shown for each volunteer ("Maximum local" and "Maximum systemic").

Supplementary figure 3. Virus neutralising antibody (VNA) levels for females and males.

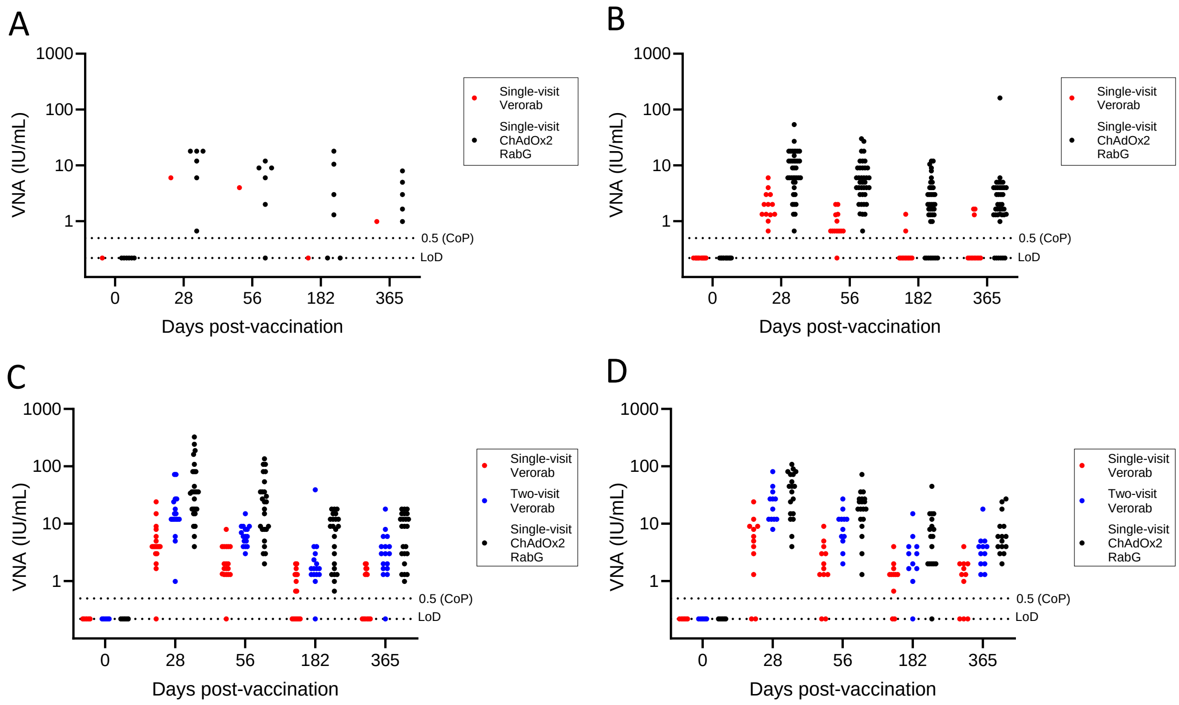

Supplementary figure 3. Virus neutralising antibody (VNA) levels for adult female (A), adult male (B), paediatric female (C) and paediatric male (D) participants; participants received either a single-visit IRV (red), two-visit IRV (blue) or single-visit, full-dose ChAdOx2 RabG (black) primary vaccination. Day 365 visits were skewed towards the end of the permissible window (days 273-546), with a median visit day of 523 for adults and 432 for children. Dotted lines indicate the limit of detection (LoD) and the 0.5 IU/mL level indicative of an adequate response to vaccination (correlate of protection; CoP) as indicated.

Supplementary figure 4. Duration of maintenance of VNA ≥0.5 IU/mL.

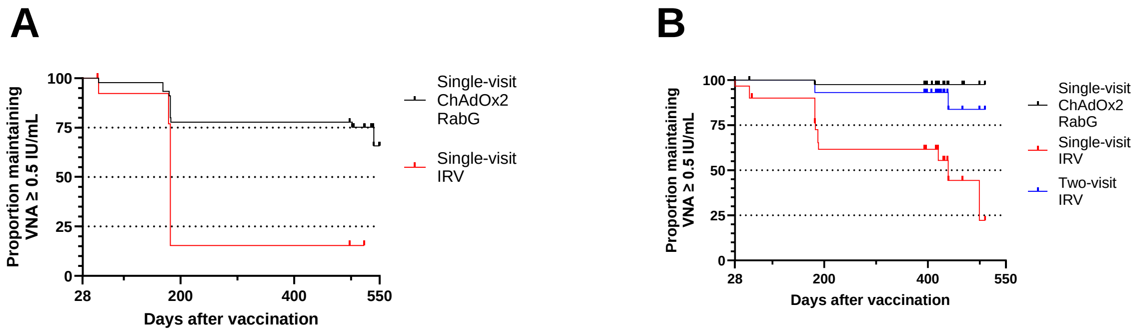

Nominal visit day	28	182	365
Median actual visit day	28	182	523
At risk, ChAdOx2 RabG (n)	45	44	35
At risk, single-visit IRV (n)	14	12	2
Censored, ChAdOx2 RabG (n, cumulative)	0	0	33
Censored, single-visit IRV (n, cumulative)	0	1	3

Nominal visit day	28	182	365
Median actual visit day	28	182	432
At risk, ChAdOx2 RabG (n)	45	41	37
At risk, single-visit IRV (n)	30	26	17
At risk, two-visit IRV (n)	29	29	27
Censored, ChAdOx2 RabG (n, cumulative)	1	7	44
Censored, single-visit IRV (n, cumulative)	0	2	16
Censored, two-visit IRV (n, cumulative)	0	0	26

Supplementary figure 4. Duration of maintenance of VNA ≥0.5 IU/mL.

Maintenance of seropositivity is represented by Kaplan-Meier survival curves for adults (A) and children (B) who received single-visit, full-dose ChAdOx2 RabG (black), single-visit IRV (red), or two-visit IRV (blue, children only).

X-axis shows actual days after vaccination, rather than nominal visit timepoint. Symbols indicate censoring, i.e. the last visit date for volunteers with VNA maintained at ≥0.5 IU/mL until that day. Tables under each panel indicate numbers at risk in each group *versus* time, and the cumulative number in each group censored at or before each nominal visit timepoint. Where the latter exceeds the number of symbols visible on the plot, this is because of censoring of multiple individuals on the same day, i.e. overlaid symbols. Individuals may be both 'at risk' and then censored at the same visit.

Participants were considered to be seropositive and 'at risk' from day 28, with the exception of the one paediatric single-visit IRV recipient who never seroconverted. For simplicity, the two paediatric single-visit IRV recipients who seroconverted 'late' (at day 56) were considered to be at risk from day 28.

Participants were considered to lose seropositivity when they first returned a seronegative result (irrespective of any later positive results). This is a conservative approach as several participants did return VNA <0.5 IU/mL at day 182 but ≥0.5 IU/mL at day 365, perhaps due to assay variability.

The small number of participants who missed a day 56 or day 182 visit (for n, see supplementary tables 10 and 11) but did return for later visit(s) were considered to remain at risk until the final visit which they did attend.

Comparative survival curve analysis, using a log-rank test of time until VNA<0.5, was listed by the SAP as an optional exploratory analysis. This was not performed using the current data set because the interval censoring of the data violates an assumption of the log-rank test. This will instead be performed when additional data is available to permit estimation of this time as a continuous variable by modelling antibody kinetics.

Supplementary figure 5. Relationship of VNA and total IgG ELISA results.

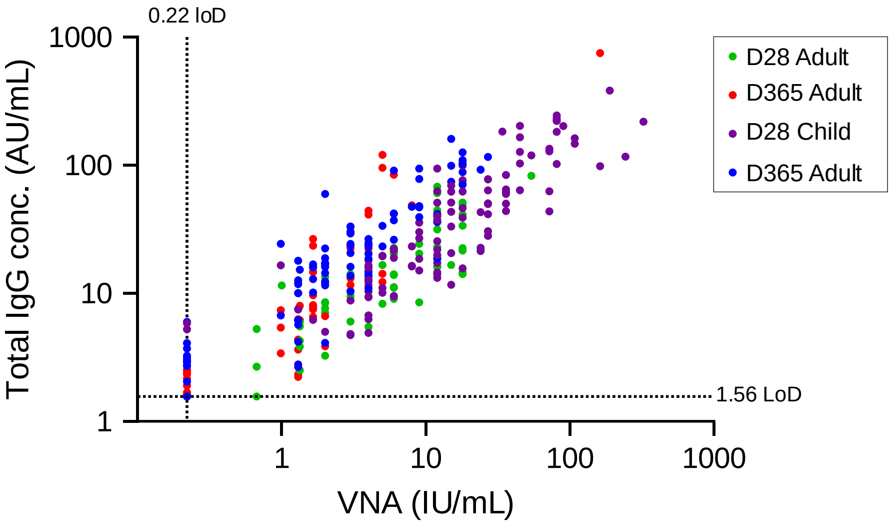

Supplementary figure 5. Relationship of VNA and total IgG ELISA results.

Data for all full-dose ChAd and IRV participants combined, by timepoint and age group: adults 28 days post vaccination (green), adults 365 days post vaccination (red), children 28 days post-vaccination (purple), and children 365 days post-vaccination (blue). Dotted lines indicate the limit of detection (LoD) for each assay. Day 365 visits were skewed towards the end of the permissible window (days 273-546), with a median visit day of 523 for adults and 432 for children.

Supplementary figure 6. Total anti-RVG IgG ELISA results following sPEP.

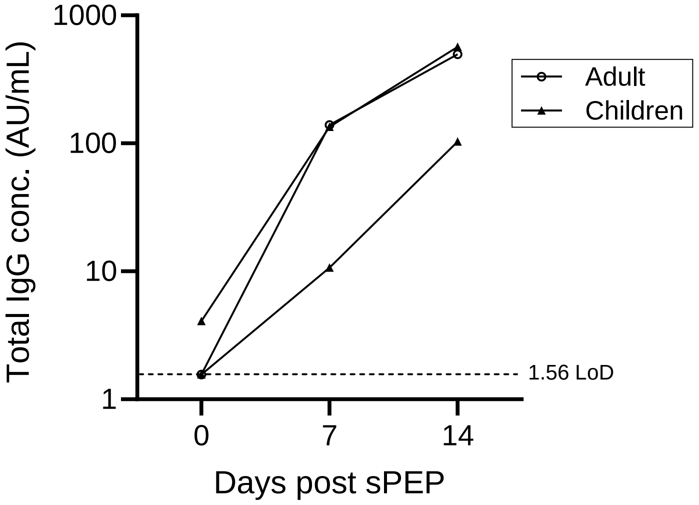

Supplementary figure 6. Total anti-RVG IgG ELISA results following sPEP.

One adult (open circle) and two child (closed triangles) participants who had received single-visit IRV were randomised to receive sPEP at the nominal day 365 visit and their anti-rabies virus glycoprotein (RVG) total IgG levels prior to sPEP initiation, 7 and 14 days later measured. LoD; limit of detection.

Supplementary figure 7. Avidity of anti-RVG IgG responses 28 days post-vaccination.

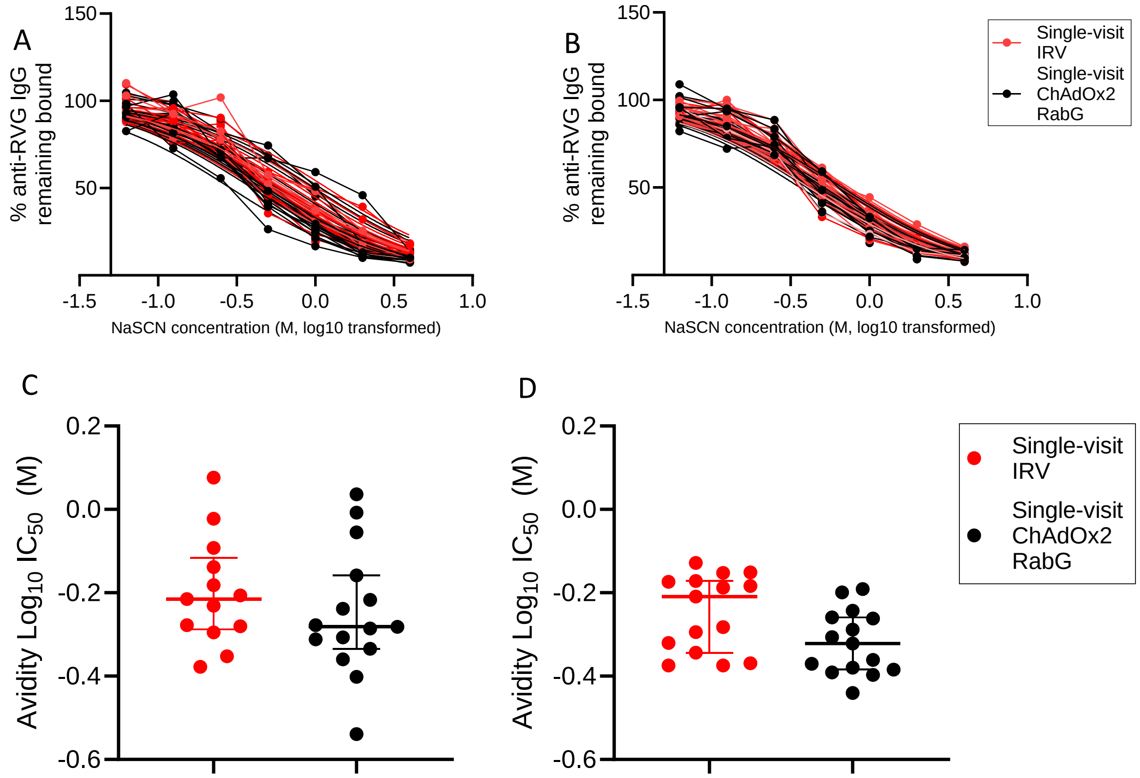

Supplementary figure 7. Avidity of anti-RVG IgG responses 28 days post-vaccination.

(A & B): Avidity of anti-RVG total IgG responses in adult (A) and child (B) 'immunology subset' participants were measured by ELISA where samples were incubated with varying concentrations of the chaotropic agent sodium thiocyanate (NaSCN) or control (no NaSCN). For each sample, data from incubation with the control was used to set the 100% rate for antibody binding, and then relative inhibition at each NaSCN concentration quantified.

(C & D): The log_{10} IC_{50} from each sample was plotted for adults (C) and children (D). Responses were found to not be significantly different for adults, while statistically higher avidity was found for paediatric IRV recipients (p=0.25 for adults and p=0.021 for children, 2-tailed Mann Whitney test, post-hoc analysis).

Supplementary figure 8. Anti-ChAdOx1 vector neutralising antibody titres.

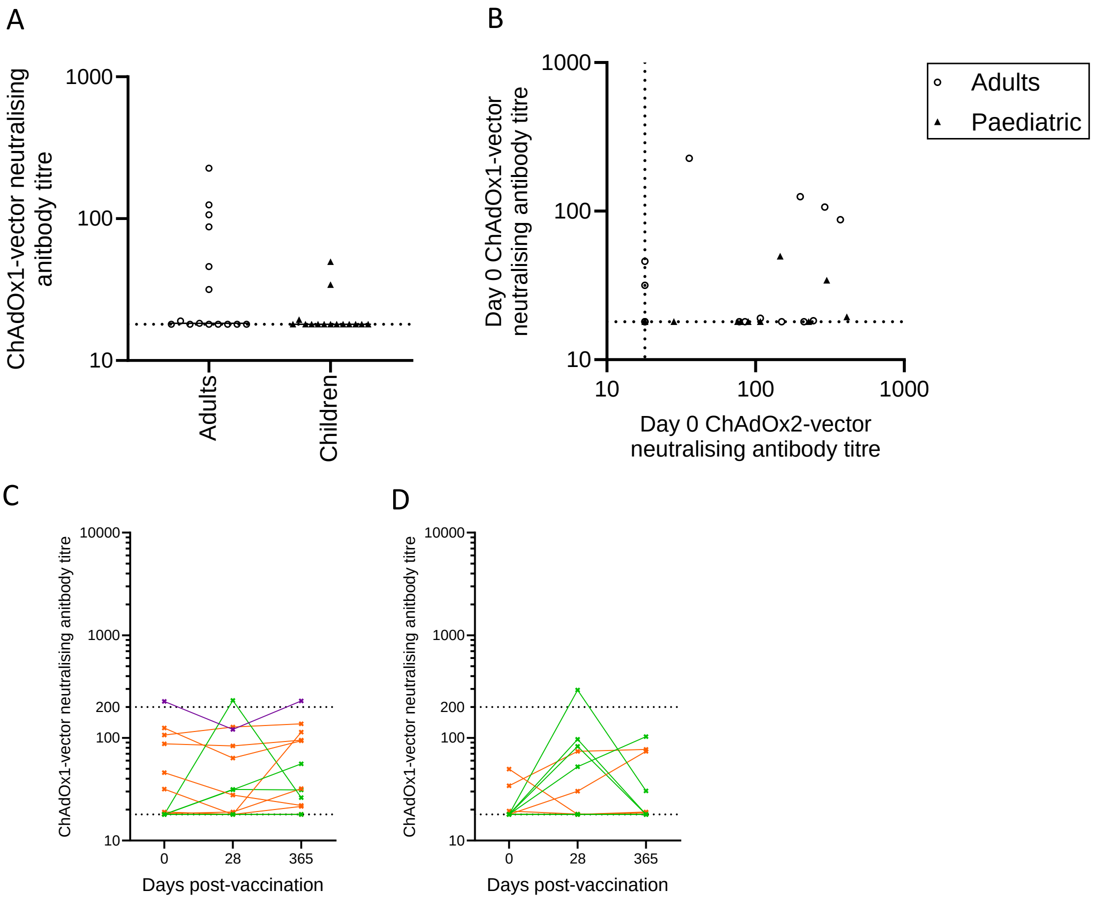

Supplementary figure 8. **Anti-ChAdOx1 vector neutralising antibody titres**.

Dotted lines represent the LoD for the neutralisation assay.

(A): Baseline (day 0) anti-ChAdOx1 vector neutralising antibody titres for adults (open circles) and children (closed triangles) who received a full-dose of ChAdOx2 RabG.

(B): Pooled analysis of adult (open circles) and child (closed triangles) participants showed a significant correlation between baseline AVA ChAdOx1 and ChAdOx2 responses (n=30). Spearman rank correlation for pooled data; p=0.0175, ρ 0.4305 (95% CI 0.07209 to 0.6905).

(C & D): Anti-ChAdOx1 vector antibody (AVA) kinetics in adults (C) and children (D). To investigate anti-vector antibody (AVA) kinetics in adults (C) and children (D) with a range of pre-vaccination titres, day 28 and nominal day 365 AVA titres were measured for five adult participants randomly selected from each of three strata defined by baseline anti-ChAdOx2 AVA titre. Colour coding is based on anti-ChAdOx1 AVA titre at day 0 (negative (≤18, green); moderate (>18, <200, orange); and high (≥200, purple)). For children, there were only 3 participants with a high anti-ChAdOx2 baseline titre, all of whom were included. 7 moderate and 5 negative anti-ChAdOx2 child participants were randomly selected. Day 365 visits were skewed towards the end of the permissible window (days 273-546), with a median visit day of 523 for adults and 432 for children.

Supplementary tables

Supplementary table 1. Deviations from the protocol.

Line listing of all deviations from the protocol reported in the study up to and including the nominal day 365 visit.

Type	Details
Dosing error	One adult participant received ChAdOx2 RabG as two half-dose injections, one in each deltoid, approximately 2 hours apart
Missed visits	Four adult participants missed a total of six study visits
Missed visits	Sixteen child participants missed a total of twenty-two study visits
Visit window	Twelve adult participants attended their day 365 visit one to four days outside the window, and are included in VNA data analyses
Visit window	One child participant attended their day 56 visit one day outside the window, and are included in the VNA analyses
Visit window	One child participant, who had lost contact with the study team, completed their day 365 visit seventy days outside the window and were excluded from VNA analyses
Sampling error	One participant in immunology subset did not have extra samples collected for exploratory immunology at all visits
Missing data	Samples from one adult and one child participant who attended the day 365 visit in window did not have samples available for VNA analysis for that visit
No sampling	One child participant was diagnosed with a pre-existing medical after completion of the day 28 visit, and no more blood samples were taken once the condition was identified

Supplementary table 2. Serious adverse events

Line listing of all serious adverse events (SAEs) reported in the study up to the time of paper preparation in September 2025.

Group	ChAdOx2 RabG dose and route	Serious Adverse Event (MedDRA PT)	Number of days after vaccination SAE was reported	Outcome	Relatedness
Group AC3, full dose	5×10^{10} vp IM	Miscarriage (Hospitalisation)	105	Resolved	Unlikely Related
Group AC3, full dose	5×10^{10} vp IM	Miscarriage (Hospitalisation)	875	Resolved	Unlikely Related
Group AC3, full dose	5×10^{10} vp IM	Miscarriage (Hospitalisation)	938	Resolved	Unlikely Related

Supplementary table 3. Local and systemic adverse events in adult groups, broken down by participant sex.

Solicited AEs by adult group and sex for 7 days after vaccination (D0-D6). Data are presented as; number of participants reporting event (% participants reporting event; 95% CI for % participants); number of events reported. "NA" indicates insufficient data points to generate result, due to there being no participants of that sex in that group. Confidence intervals (CIs) are not reported when group n≤3. Further to the data in this table, one male (group AC2) reported headache on day 7.

	Sex	Group AC1, mid dose $2.5x10^{10}$ vp ChAdOx2 RabG	Groups AC2/AC3, full dose $5x10^{10}$ vp ChAdOx2 RabG	Group AV1 1 visit IRV
Number of participants	Female	0	6	1
Number of participants	Male	3	39	14
Any local solicited adverse event	Female	NA	2 (33%;4-85); 2	0
Any local solicited adverse event	Male	1 (33%;-); 1	17 (44%;28-60); 28	2 (14%;3-48); 9
Pain	Female	NA	2 (33%;4-85); 2	0
Pain	Male	1 (33%;-); 1	17 (44%;28-60); 28	0
Swelling	Female	NA	0	0
Swelling	Male	0	0	2 (14%;3-48); 6
Induration	Female	NA	0	0
Induration	Male	0	0	1 (7%;1-44); 3
Any system solicited adverse event	Female	NA	2 (33%;4-85); 6	0
Any system solicited adverse event	Male	1 (33%;-); 1	19 (49%;33-65); 50	5 (36%;14-66); 9
Fever	Female	NA	1 (17%;1-81); 1	0
Fever	Male	0	4 (10%;4-25); 4	0
Feverishness	Female	NA	2 (33%;4-85); 2	0
Feverishness	Male	1 (33%;-); 1	13 (33%;20-50); 13	2 (14%;3-48); 2
Headache	Female	NA	2 (33%;4-85); 2	0
Headache	Male	0	12 (31%;18-48); 12	4 (29%;10-60); 5
Nausea	Female	NA	0	0
Nausea	Male	0	2 (5%;1-19); 2	0
Fatigue	Female	NA	0	0
Fatigue	Male	0	8 (21%;10-37); 8	0
Joint pain	Female	NA	1 (17%;1-81); 1	0
Joint pain	Male	0	3 (8%;2-22); 3	2 (14%;3-48); 2
Muscle pain	Female	NA	0	0
Muscle pain	Male	0	3 (8%;2-22); 3	0
Malaise	Female	NA	0	0
Malaise	Male	0	5 (10%;4-25); 5	0

Supplementary table 4. Local and systemic adverse events in paediatric groups, broken down by participant sex.

Solicited AEs by paediatric group and sex for 7 days after vaccination (D0-D6). Data are presented as; number of participants reporting event (% participants reporting event; 95% CI for % participants); number of events reported. Confidence intervals (CIs) are not reported when group n≤3. Further to the data in this table, reduced oral intake was reported for one female (group PC1b) and feverishness for one male (group PV2) on day 7.

	Sex	Group PC1a, low dose $1x10^{10}$ vp ChAdOx2 RabG	Group PC1b, mid dose $2.5x10^{10}$ vp ChAdOx2 RabG	Groups PC2/PC3, full dose $5x10^{10}$ vp ChAdOx2 RabG	Group PV1 1 visit IRV	Group PV2 2 visit IRV
Number of participants	Female	1	2	19	12	13
Number of participants	Male	2	1	26	18	17
Any local solicited adverse event	Female	0	0	0	0	0
Any local solicited adverse event	Male	1 (50%,-); 3	0	1 (4%;0-25); 1	1 (6%;1-35); 1	0
Pain	Female	0	0	0	0	0
Pain	Male	1 (50%;-); 3	0	1 (4%;0-25); 1	1 (6%;1-35); 1	0
Swelling	Female	0	0	0	0	0
Swelling	Male	0	0	0	0	0
Induration	Female	0	0	0	0	0
Induration	Male	0	0	0	0	0
Any system solicited adverse event	Female	1 (100%;-); 1	0	5 (26%;10-52); 7	0	1 (8%;1-47); 1
Any system solicited adverse event	Male	1 (50%;-); 2	0	10 (38%;21-59); 14	0	1 (6%;1-37); 1
Fever	Female	0	0	2 (11%;2-37); 2	0	1 (8%;1-47); 1
Fever	Male	1 (50%;-); 1	0	3 (12%;3-32); 3	0	1 (6%;1-37); 1
Feverishness	Female	0	0	4 (21%;7-47); 4	0	0
Feverishness	Male	0	0	7 (27%;13-48); 7	0	0
Vomit	Female	0	0	1 (5%;1-34); 1	0	0
Vomit	Male	0	0	1 (4%;0-25); 1	0	0
Diarrhoea	Female	0	0	0	0	0
Diarrhoea	Male	0	0	1 (4%;0-25); 1	0	0
Reduced activities	Female	0	0	0	0	0
Reduced activities	Male	1 (50%;-); 1	0	0	0	0
Reduced oral intake	Female	1 (100%;-); 1	0	0	0	0
Reduced oral intake	Male	0	0	2 (8%;2-28); 2	0	0

Supplementary table 5. Unsolicited adverse events (AEs) in adult participants.

Line listing of related and unrelated unsolicited adverse events reported within 28 days of vaccination on day 0. Presented by MedDRA System Organ Class (SOC), MedDRA Preferred Terms (PT) and severity grade.

	Participants reporting AE, n (%)			
	**Group AC1 Mid dose (n=3)**	**Groups AC2/AC3 Full dose (n=45)**	**Group AV1 1 visit IRV (n=15)**	**Total All groups (n=63)**
**AEs assessed as "possibly", "probably" or "definitely" related to vaccination**				
**Respiratory, thoracic and mediastinal disorders**	**-**	**2 (4%)**	**1 (7%)**	**3 (5%)**
Upper respiratory tract infection				
Grade 1	-	2 (4%)	-	2 (3%)
Acute tonsillitis				
Grade 1	-	-	1 (7%)	1 (2%)
**Participants with ≥1 unsolicited AE assessed as "possibly", "probably" or "definitely" related to vaccination**	**-**	**2 (4%)**	**1 (7%)**	**3 (5%)**
**AEs assessed as "No relationship" or "Unlikely related" to vaccination**				
**Respiratory, thoracic and mediastinal disorders**	**-**	**4 (9%)**	**2 (13%)**	**6 (10%)**
Upper respiratory tract infection				
Grade 1	-	4 (9%)	2 (13%)	6 (10%)
**Gastrointestinal disorders**	**-**	**2 (4%)**	**-**	**2 (3%)**
Gastritis				
Grade 1	-	1 (2%)	-	1 (2%)
Gastroenteritis				
Grade 1	-	1 (2%)	-	1 (2%)
**Infections and infestations**	**1 (33%)**	**7 (16%)**	**-**	**8 (13%)**
Food poisoning				
Grade 1	1 (33%)	1 (2%)	-	2 (3%)
Urinary tract infection				
Grade 1	-	6 (13%)	-	6 (10%)
**Skin and subcutaneous tissue disorders**	**-**	**2 (4%)**	**-**	**2 (3%)**
Burn				
Grade 1	-	1 (2%)	-	1 (2%)
Tinea corporis				
Grade 1	-	1 (2%)	-	1 (2%)
**Participants with ≥1 unsolicited AE assessed as "no relationship" or "unlikely" related to vaccination**	**1 (33%)**	**14 (31%)**	**2 (13%)**	**17 (27%)**

Supplementary table 6. Unsolicited adverse events (AEs) in paediatric participants.

Line listing of related and unrelated unsolicited adverse events reported within 28 days of vaccination on day 0. Presented by MedDRA System Organ Class (SOC), MedDRA Preferred Terms (PT) and severity grade.

	Participants reporting AE, n (%)					
	**Group PC1a Low dose (n=3)**	**Group PC1b Mid dose (n=3)**	**Groups PC2/PC3 Full dose (n=45)**	**Group PV1 1 visit IRV (n=30)**	**Group PV2 2 visit IRV (n=30)**	**Total All groups (n=111)**
**AEs assessed as "possibly", "probably" or "definitely" related to vaccination**						
**General disorders and administration site conditions**	**-**	**-**	**7 (16%)**	**-**	**1 (3%)**	**8 (7%)**
Pyrexia						
Grade 1	-	-	6 (13%)	-	1 (3%)	7 (6%)
Grade 2	-	-	1 (2%)	-	-	1 (1%)
**Respiratory, thoracic and mediastinal disorders**	**-**	**-**	**2 (4%)**	**-**	**-**	**2 (2%)**
Upper respiratory tract infection						
Grade 1	-	-	1 (2%)	-	-	1 (1%)
Pneumonia						
Grade 2	-	-	1 (2%)	-	-	1 (1%)
**Infections and infestations**	**-**	**-**	**-**	**-**	**1 (3%)**	**1 (1%)**
Food poisoning						
Grade 2	-	-	-	-	1 (3%)	1 (1%)
**Participants with ≥1 unsolicited AEs assessed as "possibly", "probably" or "definitely" related to vaccination**	**-**	**-**	**9 (20%)**	**-**	**2 (7%)**	**11 (10%)**
**AEs assessed as "No relationship" or "Unlikely related" to vaccination**						
**General disorders and administration site conditions**	**-**	**-**	**-**	**1 (3%)**	**1 (3%)**	**2 (2%)**
Pyrexia						
Grade 1	-	-	-	1 (3%)	1 (3%)	2 (2%)
**Respiratory, thoracic and mediastinal disorders**	**-**	**1 (33%)**	**2 (4%)**	**2 (7%)**	**-**	**5 (5%)**
Upper respiratory tract infection						
Grade 1	-	-	-	1 (3%)	-	1 (1%)
Pneumonia						
Grade 1	-	-	1 (2%)	-	-	1 (1%)
Grade 2	-	1 (33%)	-	-	-	1 (1%)
Nasopharyngitis						
Grade 1	-	-	-	1 (3%)	-	1 (1%)
Tonsillitis						
Grade 2	-	-	1 (2%)	-	-	1 (1%)
**Infections and infestations**	**-**	**1 (33%)**	**1 (2%)**	**1 (3%)**	**1 (3%)**	**4 (4%)**
Urinary tract infection						
Grade 1	-	-	1 (2%)	-	-	1 (1%)
Grade 2	-	1 (33%)	-	1 (3%)	1 (3%)	3 (3%)
Septicaemia NOS						
Grade 1	-	-	1 (2%)	-	-	1 (1%)
**Gastrointestinal disorders**	**-**	**-**	**1 (2%)**	**2 (7%)**	**-**	**3 (3%)**
Gastritis						
Grade 2	-	-	-	1 (3%)	-	1 (1%)
Gastroenteritis			-			
Grade 1	-	-	-	1 (3%)	-	1 (1%)
Trichuriasis						
Grade 1	-	-	1 (2%)	-	-	1 (1%)
**Skin and subcutaneous tissue disorders**	**-**	**-**	**-**	**1 (3%)**	**2 (7%)**	**3 (3%)**
Dermatitis						
Grade 1	-	-	-	-	1 (3%)	1 (1%)
Scabies						
Grade 1	-	-	-	-	1 (3%)	1 (1%)
Skin fungal infection						
Grade 2	-	-	-	1 (3%)	-	1 (1%)
**Musculoskeletal and connective tissue disorders**	**-**	**-**	**1 (2%)**	**-**	**-**	**1 (1%)**
Muscle strain						
Grade 2	-	-	1 (2%)	-	-	1 (1%)
**Participants with ≥1 unsolicited AE assessed as "no relationship" or "unlikely" related to vaccination**	**-**	**2 (67%)**	**4 (9%)**	**6 (20%)**	**4 (13%)**	**16 (14%)**

Supplementary table 7: Laboratory abnormalities in adult participants.

Clinical laboratory results measured at each evaluated timepoint in the trial for all adult groups. Laboratory AEs were assessed according to pre-specified locally established laboratory reference intervals and graded by the trial site, using ranges outlined in Supplementary table 9.

	Mid dose ChAdOx2 RabG			Full dose ChAdOx2 RabG			Single-visit IRV		
	D7	D14	D28	D7	D14	D28	D7	D14	D28
**White Cell Count (n evaluated)**	**3**	**3**	**3**	**45**	**45**	**45**	**15**	**15**	**14**
Normal	3	3	3	43	41	40	12	15	12
Grade 1 Decreased WCC	-	-	-	2	3	3	3	-	-
Grade 2 Decreased WCC	-	-	-	-	-	1	-	-	-
Grade 1 Increased WCC	-	-	-	-	1	1	-	-	2
**Haemoglobin (n evaluated)**	**3**	**3**	**3**	**45**	**45**	**45**	**15**	**15**	**14**
Normal	3	3	3	44	43	42	15	14	14
Grade 1 Decreased Hb	-	-	-	1	2	3	-	1	-
**Platelets (n evaluated)**	**3**	**3**	**3**	**45**	**45**	**45**	**15**	**15**	**14**
Normal	3	3	3	44	42	45	14	13	13
Grade 1 Decreased Platelets	-	-	-	1	2	-	1	2	1
Grade 1 Decreased Platelets	-	-	-	-	1	-	-	-	-
**Neutrophils (n evaluated)**	**3**	**3**	**3**	**45**	**45**	**45**	**15**	**15**	**14**
Normal	3	3	3	42	42	41	14	15	14
Grade 1 Decreased Neutrophils	-	-	-	3	1	3	1	-	-
Grade 2 Decreased Neutrophils	-	-	-	-	2	1	-	-	-
**Lymphocytes (n evaluated)**	**3**	**3**	**3**	**45**	**45**	**45**	**15**	**15**	**14**
Normal	3	3	3	45	43	43	14	15	14
Grade 1 Decreased Lymphocytes	-	-	-	-	1	2	1	-	-
Grade 3 Decreased Lymphocytes	-	-	-	-	1	-	-	-	-
**Eosinophils (n evaluated)**	**3**	**3**	**3**	**45**	**45**	**45**	**15**	**15**	**14**
Normal	3	3	3	43	44	45	14	15	14
Grade 1 Increased Eosinophils	-	-	-	2	1	-	1	-	-
**ALT (n evaluated)**	**3**	**3**	**3**	**45**	**45**	**45**	**15**	**15**	**14**
Normal	3	3	3	45	45	45	14	15	14
Grade 1 Increased ALT	-	-	-	-	-	-	1	-	-
**Creatinine (n evaluated)**	**3**	**3**	**3**	**45**	**45**	**45**	**15**	**15**	**14**
Normal	3	3	3	44	43	42	13	13	12
Grade 1 Increased Creatinine	**-**	-	-	1	2	3	2	2	2

Supplementary table 8: Laboratory abnormalities in child participants.

Clinical laboratory results measured at each evaluated timepoint in the trial for all paediatric groups. Laboratory AEs were assessed according to pre-specified locally established laboratory reference intervals and graded by the trial site, using ranges outlined in Supplementary table 9.

	**Low dose ChAdOx2 RabG**			**Mid dose ChAdOx2 RabG**			**Full dose ChAdOx2 RabG**			**Single-visit IRV**			**Two-visit IRV**		
	**D7**	**D14**	**D28**	**D7**	**D14**	**D28**	**D7**	**D14**	**D28**	**D7**	**D14**	**D28**	**D7**	**D14**	**D28**
**White Cell Count (n evaluated)**	**3**	**3**	**3**	**3**	**3**	**3**	**45**	**44**	**45**	**30**	**30**	**30**	**29**	**30**	**29**
Normal	3	3	3	3	3	3	43	41	44	28	28	27	28	30	28
Grade 1 Decreased WCC	-	-	-	-	-	-	2	1	1	2	2	3	1	-	1
Grade 1 Increased WCC	-	-	-	-	-	-	-	1	-	-	-	-	-	-	-
Grade 3 Increased WCC	-	-	-	-	-	-	-	1	-	-	-	-	-	-	-
**Haemoglobin (n evaluated)**	**3**	**3**	**3**	**3**	**3**	**3**	**45**	**44**	**45**	**30**	**30**	**30**	**29**	**30**	**29**
Normal	3	3	3	3	3	2	44	43	44	30	29	30	29	30	29
Grade 1 Decreased Hb	-	-	-	-	-	1	-	-	-	-	1	-	-	-	-
Grade 2 Decreased Hb	-	-	-	-	-	-	-	1	1	-	-	-	-	-	-
Grade 3 Decreased Hb	-	-	-	-	-	-	1	-	-	-	-	-	-	-	-
**Platelets (n evaluated)**	**3**	**3**	**3**	**3**	**3**	**3**	**45**	**44**	**45**	**30**	**30**	**30**	**29**	**30**	**29**
Normal	3	3	3	3	3	3	45	44	45	30	30	30	29	30	29
**Neutrophils (n evaluated)**	**3**	**3**	**3**	**3**	**3**	**3**	**45**	**44**	**45**	**30**	**30**	**30**	**29**	**30**	**29**
Normal	3	3	3	3	3	3	44	43	45	27	30	26	29	28	29
Grade 1 Decreased Neutrophils	-	-	-	-	-	-	1	1	-	3	-	3	-	1	-
Grade 2 Decreased Neutrophils	-	-	-	-	-	-	-	-	-	-	-	1	-	-	-
Grade 3 Decreased Neutrophils	-	-	-	-	-	-	-	-	-	-	-	-	-	1	-
**Lymphocytes (n evaluated)**	**3**	**3**	**3**	**3**	**3**	**3**	**45**	**44**	**45**	**30**	**30**	**30**	**29**	**30**	**29**
Normal	3	3	3	3	3	3	45	43	44	29	30	30	28	29	28
Grade 1 Decreased Lymphocytes	-	-	-	-	-	-	-	1	1	1	-	-	1	1	1
**Eosinophils (n evaluated)**	**3**	**3**	**3**	**3**	**3**	**3**	**45**	**44**	**45**	**30**	**30**	**30**	**29**	**30**	**29**
Normal	3	3	3	3	3	3	44	44	45	30	29	30	29	30	29
Grade 1 Increased Eosinophils	-	-	-	-	-	-	1	-	-	-	1	-	-	-	-
**ALT (n evaluated)**	**3**	**3**	**3**	**3**	**3**	**3**	**45**	**44**	**45**	**30**	**30**	**30**	**29**	**30**	**29**
Normal	3	3	3	3	3	2	43	42	43	29	30	30	29	30	28
Grade 1 Increased ALT	-	-	-	-	-	1	2	2	2	-	-	-	-	-	1
Grade 2 Increased ALT	-	-	-	-	-	-	-	-	-	1	-	-	-	-	-
**Creatinine (n evaluated)**	**3**	**3**	**3**	**3**	**3**	**3**	**44**	**44**	**45**	**30**	**30**	**30**	**29**	**30**	**29**
Normal	**3**	3	3	3	3	3	44	43	45	30	30	30	29	29	29
Grade 1 Increased Creatinine	-	-	-	-	-	-	-	1	-	-	-	-	-	-	-
Grade 2 Increased Creatinine	-	-	-	-	-	-	-	-	-	-	-	-	-	1	-

Supplementary table 9. Ranges used to grade laboratory abnormalities.

For adult and child participants in Supplementary table 7 and Supplementary table 8 respectively.

Parameter	Units	Age range	Normal range	Grade 1 Mild	Grade 2 Moderate	Grade 3 Severe
White Cell Count Decreased	$10^3/\mu l$	6 months - 5 years	5.1 - 16.2	2 - < 5.1	1.5 - 1.999	1 - 1.499
		6 - 10 years	4.5 - 12.9	2 - < 4.5		
		>10 years	3.48 - 9.11	2.5 - < 3.48	1.5 - 2.499	
White Cell Count Increased	$10^3/\mu l$	6 months - 5 years	5.1 - 16.2	> 16.2 - 18	> 18 - 20	> 20 - 25
		6 - 10 years	4.5 - 12.9	> 12.9 - 16	> 16 - 20	
		>10 years	3.48 - 9.11	> 9.11 - 15	> 15 - 20	
Haemoglobin Decreased	gm/dL	6 months - 5 years	8.6 - 13.2	8 - < 8.6	7 - < 8	<7
		6 - 10 years	9.6 - 14.1	9 - < 9.6	7 - < 9	
Haemoglobin Decreased (Female)		>10 years	9.6 - 14.1			
Haemoglobin Decreased (Male)			12.6 - 17.3	9 - < 12.6		
Platelets Decreased	$10^3/\mu l$	6 months - 5 years	91 - 491	75 - < 91	50 - < 75	25 - < 50
		6 - 10 years	91 - 456			
		>10 years	107 - 396.2	75 - < 107		
Neutrophils Decreased	$10^3/\mu l$	6 months - 5 years	1.3 - 6.9	0.8 - < 1.3	0.6 - 0.799	0.4 - 0.599
		6 - 10 years	1.2 - 6.2	0.8 - < 1.2		
		>10 years	1.18 - 5.46	0.8 - < 1.18		
Lymphocytes Decreased	$10^3/\mu l$	6 months - 5 years	2.1 - 9.5	0.8 - < 2.1	0.5 - < 0.8	0.2 - < 0.5
		6 - 10 years	1.8 - 6.4	0.8 - < 1.8		
		>10 years	1.19 - 3.4	0.8 - < 1.19		
Eosinophils Increased	$10^3/\mu l$	6 months - 5 years	0.1 - 1.6	> 1.6 - 2.4	> 2.4 - 4.8	> 4.8
		6 - 10 years	0.1 - 2.1	> 2.1 - 3.2	> 3.2 - 6.3	> 6.3
		>10 years	0 - 0.78	> 0.78 - 1.5	> 1.5 - 5	> 5
ALT Increased	U/L	1-5 years	10 - 28	> 28 - < 70	70 - < 140	140 - < 280
		6 - 10 years	9 - 35	> 35 - < 87.5	87.5 - < 175	175 - < 350
		>10 years	3.5 - 46.8	> 46.8 - 117	> 117 - 234	> 234 - 468
Creatinine Increased	µmol/L	1-5 years	15 - 50	> 50 - 65	> 65 - 90	> 90 - < 175
		6 - 10 years	24 - 49	> 49 - 63.7	> 63.7 - 88.2	> 88.2 - < 171.5
		>10 years	49 - 95.3	> 95.3 - 150.28	> 150.28 - 176.8	> 176.8 - 221

Supplementary table 10. Summary of VNA data for adult groups from day 28.

The limit of detection for the VNA assay was 0.22 IU/mL. For calculation of geometric means and confidence intervals, negative results were assigned a value of 0.22. * Indicates one participant who had VNA >0.5 IU/mL at D365 but did not consent for study extension beyond 1 years and received sPEP at the one year visit in accordance with the protocol. CIs are not reported when n≤3.

Visit day	Statistic	Group AC1, mid dose $2.5x10^{10}$ vp ChAdOx2 RabG	Groups AC2/AC3, full dose $5x10^{10}$ vp ChAdOx2 RabG	Group AV1 1 visit IRV
28	Number of people	3	45	14
	n with VNA <0.5	0	0	0
	n with VNA ≥0.5	3	45	14
	Min	1	1	1
	Q1	1	5	1
	Median	4	9	2
	Q3	6	18	3
	Max	6	54	6
	Geometric mean	3.2	7.4	2.0
	95% CI	–	5.5,9.9	1.4,3.0
56	Number of people	3	44	14
	n with VNA <0.5	0	1	1
	n with VNA ≥0.5	3	43	13
	Min	1	≤0.22	≤0.22
	Q1	1	3	1
	Median	2	6	1
	Q3	2	9	1
	Max	2	30	4
	Geometric mean	1.4	4.8	0.9
	95% CI	–	3.6,6.4	0.6, 1.4
182	Number of people	3	45	13
	n with VNA <0.5	1	10	11
	n with VNA ≥0.5	2	35	2
	Min	≤0.22	≤0.22	≤0.22
	Q1	≤0.22	1	≤0.22
	Median	1	2	≤0.22
	Q3	1	4	≤0.22
	Max	1	18	1
	Geometric mean	0.7	1.7	0.3
	95% CI	–	1.2, 2.5	0.2,0.4
Nominal 365 (median 523) (includes sPEP+0)	Number of people	3	44	13
	n with VNA <0.5	2	6	9
	n with VNA ≥0.5	1	38	4
	Min	≤0.22	≤0.22	≤0.22
	Q1	≤0.22	1	≤0.22
	Median	≤0.22	2	≤0.22
	Q3	1	4	1
	Max	1	162	2
	Geometric mean	0.4	2	0.4
	95% CI	–	1.4,2.9	0.2,0.7
sPEP+7	Number of people	–	1*	1
	n with VNA <0.5	–	0	0
	n with VNA ≥0.5	–	1	1
	Min	–	108	12
	Q1	–	108	12
	Median	–	108	12
	Q3	–	108	12
	Max	–	108	12
	Geometric mean	–	108	12
	95% CI	–	–	–
sPEP+14	Number of people	–	1*	1
	n with VNA <0.5	–	0	0
	n with VNA ≥0.5	–	1	1
	Min	–	243	45
	Q1	–	243	45
	Median	–	243	45
	Q3	–	243	45
	Max	–	243	45
	Geometric mean	–	243	45
	95% CI	–	–	–

Supplementary table 11. Summary of VNA data for paediatric groups from day 28.

The limit of detection for the VNA assay was 0.22 IU/mL. For calculation of geometric means and confidence intervals, negative results were assigned a value of 0.22. CIs are not reported when n≤3.

Visit day	Statistic	Group PC1a, low dose $1x10^{10}$ vp ChAdOx2 RabG	Group PC1b, mid dose $2.5x10^{10}$ vp ChAdOx2 RabG	Groups PC2/PC3, full dose $5x10^{10}$ vp ChAdOx2 RabG	Group PV1 1 visit IRV	Group PV2 2 visit IRV
28	Number of people	3	3	45	30	29
	n with VNA <0.5	0	0	0	3	0
	n with VNA ≥0.5	3	3	45	27	29
	Min	15	5	4	≤0.22	1
	Q1	15	5	15	3	12
	Median	45	8	36	4	15
	Q3	75	36	81	8	27
	Max	75	36	324	24	81
	Geometric mean	37.0	11.3	33.6	3.8	16.6
	95% CI	–	–	24.5,46.1	2.5,6.0	11.9,23.2
56	Number of people	3	3	44	30	28
	n with VNA <0.5	0	0	0	3	0
	n with VNA ≥0.5	3	3	44	27	28
	Min	9	2	1	≤0.22	2
	Q1	9	2	9	1	5
	Median	12	2	21	2	7
	Q3	18	12	36	4	11
	Max	18	12	135	9	27
	Geometric mean	12.5	3.2	17.4	1.9	7
	95% CI	–	–	12.5,24.3	1.3,2.6	5.6,8.8
182	Number of people	3	2	40	28	27
	n with VNA <0.5	0	1	1	10	2
	n with VNA ≥0.5	3	1	39	18	25
	Min	2	≤0.22	≤0.22	≤0.22	≤0.22
	Q1	2	≤0.22	2	≤0.22	1
	Median	2	2	8	1	2
	Q3	5	4	12	1	4
	Max	5	4	45	4	39
	Geometric mean	2.7	0.9	5.2	0.7	2.1
	95% CI	–	-	3.6,7.5	0.5,1.0	1.4,3.2
Nominal 365 (median 432) (includes sPEP+0)	Number of people	3	3	38	28	29
	n with VNA <0.5	0	1	0	12	1
	n with VNA ≥0.5	3	2	38	16	28
	Min	2	≤0.22	1	≤0.22	≤0.22
	Q1	2	≤0.22	3	≤0.22	2
	Median	3	3	6	1	3
	Q3	8	8	12	2	4
	Max	8	8	27	4	18
	Geometric mean	3.4	1.7	6.1	0.7	3
	95% CI	–	–	4.5,8.2	0.5,1.1	2.2,4.1
sPEP+7	Number of people	–	–	–	2	–
	n with VNA <0.5	–	–	–	0	–
	n with VNA ≥0.5	–	–	–	2	–
	Min	–	–	–	4	–
	Q1	–	–	–	4	–
	Median	–	–	–	11	–
	Q3	–	–	–	18	–
	Max	–	–	–	18	–
	Geometric mean	–	–	–	8.5	–
	95% CI	–	–	–	-	–
sPEP+14	Number of people	–	–	–	2	–
	n with VNA <0.5	–	–	–	0	–
	n with VNA ≥0.5	–	–	–	2	–
	Min	–	–	–	54	–
	Q1	–	–	–	54	–
	Median	–	–	–	81	–
	Q3	–	–	–	108	–
	Max	–	–	–	108	–
	Geometric mean	–	–	–	76.4	–
	95% CI	–	–	–	-	–

Supplementary table 12. VNA comparisons between groups at multiple timepoints

Each row denotes a pairwise comparison between single-visit, full-dose ChAdOx2 RabG (ChAd), one-visit inactivated rabies vaccine (1V IRV), or two-visit IRV (2V IRV) regimens, within the specified adult or paediatric (paed) age group & at the specified nominal timepoint. Stars indicate the pre-specified primary immunological hypothesis tests for adults and children respectively. Other calculated values and tests are *post hoc*. In accordance with the statistical analysis plan (SAP), no correction for multiple comparison was performed. Day 365 visits were skewed towards the end of the permissible window (days 273-546), with a median visit day of 523 for adults and 432 for children.

Some data sets contain negative results. Negative results were assigned an arbitrary value equal to the lower limit of detection of the assay (i.e. the highest possible result if the sample were found to be positive with a more sensitive assay). This will tend to reduce differences between more and less immunogenic regimes, and hence be conservative with respect to estimates of difference and tests for difference.

Vaccine immunogenicity differences are typically expressed in terms of geometric mean ratios (GMRs). Because of the presence of negative results and the fact several data sets fail normality testing despite log transformation, we also report here ratios of medians. Bounds of confidence intervals for ratio of medians were estimated by first calculating and then exponentiating the bounds of the 95% Wilcoxon/Mann-Whitney confidence interval (CI) for the Hodges-Lehmann estimate of the difference between medians of log-transformed VNA. These should be treated with caution as an assumption of the Hodges-Lehmann estimate is similarity of the distributions between groups. It is a known property of confidence intervals for Hodges-Lehmann estimates that they may exclude the point estimate (or the observed median difference), as is seen for the comparison of paediatric ChAdOx2 RabG & IRV1 groups.

Because of the widespread use of GMRs in the field, the fact that the distributions of data are not grossly asymmetrical, and the fairly close agreement between calculated GMRs and median ratios, we focus on GMRs (and CIs for them) in the manuscript text.

With respect to hypothesis testing, the statistical analysis plan specified two-tailed comparative testing with $\alpha=0.05$, with Student's or Welch's t-test to be used (as appropriate to the equality of variance) for comparisons where normality tests were passed, and Mann-Whitney tests otherwise. We therefore report the appropriate t-test result only for comparisons for which these assumptions were fulfilled. For completeness we have calculated Mann-Whitney test results for all comparisons, and for simplicity in the manuscript text, we report Mann-Whitney results throughout, providing consistency of approach with the tests used for the pre-specified analyses (for which normality assumptions were failed). Applying this test consistently is again conservative (as the Mann-Whitney test is generally less powerful than a t-test). As can be seen from the table, p-values <0.05 were obtained by both methods in all cases where both a t-test and a Mann-Whitney test were performed, and thus choice of test does not influence conclusions.

Supplementary table 12: VNA comparisons between groups at multiple timepoints

Caption is provided on previous page.

Timepoint and groups compared				Geometric mean ratio (GMR) of VNA			Ratio of medians			Tests of assumptions of t-test, after log transformation			Comparative tests, after log transformation		
Nominal visit day	Age	Regimen 1	Regimen 2	GMR	Lower bound of 95% CI	Upper bound of 95% CI	Ratio	Lower bound of 95% CI	Upper bound of 95% CI	Normality, d'Agostino-Pearson p, regimen 1	Normality, d'Agostino-Pearson p, regimen 2	F-test for equal variance, p	Student's t-test, p	Welch's t-test, p	Mann-Whitney, p
28	Adult	ChAd	1V IRV	3.6	2.1	6.3	4.5	2.3	6.0	0.096	0.82	0.063	<0.0001	N/A	<0.0001
28	Paed	ChAd	1V IRV	8.7	5.2	14.7	9.0	4.5	12.0	0.90	0.0078	N/A	N/A	N/A	<0.0001
28	Paed	ChAd	2V IRV	2.0	1.3	3.2	2.4	1.2	3.0	0.90	0.015	N/A	N/A	N/A	0.0025
56	Adult	ChAd	1V IRV	5.2	3.0	9.0	8.2	3.0	9.0	0.026	0.54	N/A	N/A	N/A	<0.0001
56	Paed	ChAd	1V IRV	9.3	5.7	15.1	11.4	6.0	14.6	0.64	0.039	N/A	N/A	N/A	<0.0001
56	Paed	ChAd	2V IRV	2.5	1.7	3.7	3.2	1.8	4.0	0.64	0.88	0.0004	N/A	0.0008	<0.0001
182	Adult	ChAd	1V IRV	6.2	2.9	13.2	9.1	4.5	13.6	0.22	0.0001	N/A	N/A	N/A	<0.0001
182	Paed	ChAd	1V IRV	7.4	4.4	12.5	7.1	5.5	11.5	0.21	0.003	N/A	N/A	N/A	<0.0001
182	Paed	ChAd	2V IRV	2.5	1.4	4.3	4.8	1.5	5.0	0.21	0.075	0.33	0.0015	N/A	0.0009
*365	Adult	ChAd	1V IRV	5.1	2.5	10.6	9.1	3.0	13.6	0.0083	0.15	N/A	N/A	N/A	<0.0001*
*365	Paed	ChAd	1V IRV	8.6	5.3	14.0	4.6	5.5	13.6	0.19	0.0001	N/A	N/A	N/A	<0.0001*
365	Paed	ChAd	2V IRV	2.0	1.3	3.1	2.0	1.3	3.1	0.19	0.051	0.38	0.0017	N/A	0.0028

Supplementary table 13: Calculation of likely boosted protection

Table reports number in each category, n (%). Category 3 encompasses both participants known prior to the D365 visit to have VNA<0.5 but allocated to sPEP at the end of the trial, and participants for whom data available at the d365 visit indicated VNA<0.5 (and hence not yet allocated to sPEP at d365).

Blue shading indicates participants for whom the proportion likely to have satisfactory sPEP responses was imputed from the data from participants in the same group who did receive sPEP; this was as specified by the SAP, but the post-sPEP data was very limited. Grey shading indicates data treated as missing: in the case of participants lost to follow-up with VNA>0.5, this was as specified by the SAP; for participants with VNA<0.5 from groups in which no participants underwent D365 sPEP, this was post hoc as the SAP did not specify an approach.

The one participant in the adult ChAd group who did not consent to follow-up had VNA≥0.5 at D365 so is considered in category 1 despite having received booster vaccination at that visit, per protocol. The one participant in the paediatric single-visit Verorab group who was temporarily lost to follow-up and attended the nominal day 365 visit 70 days outside the permissible window is considered in category 4, reflecting the investigators'decision that VNA data from this late visit should not be considered in D365 analyses.

All participants for whom data was imputed or who were treated as missing in this analysis had attained VNA≥0.5 IU/mL at earlier timepoints. Although the sPEP data to support imputation is very limited, we consider it likely that such participants, who had demonstrated good early responses to vaccination, would indeed mount satisfactory sPEP responses, as seen among participants with satisfactory early responses in other PrEP studies, across a range of regimens[6-10] and among the few participants in this study who did undergo sPEP having had relatively weak initial responses. Nonetheless the small number undergoing sPEP renders the estimate of boosted protection more sensitive to this assumption than had been anticipated at the time the SAP was prepared. A fuller analysis of likely boosted protection will be carried out once more participants have completed their sPEP visits.

Supplementary table 13. Calculation of likely boosted protection

Caption is provided on previous page.

			Category ->	Category 1	Category 2	Category 3	Category 4
			Category description ->	VNA≥0.5 at d365	VNA<0.5 prior to D365. Received sPEP & sPEP+7 VNA≥0.5	VNA<0.5 at D365. Allocated to sPEP later in trial	Lost to follow-up while VNA≥0.5
		Total number vaccinated	Effect on calculation of likely boosted protection ->	Likely protected (independent of boost)	Likely protected (possibly dependent on boost)	See caption	Treated as missing.
AGE	REGIMEN						
Adult	***ChAdOx2 RabG***	45		38 (84%)	0 (0%)	6 (13%)	1 (2%)
Adult	***1-dose Verorab***	15		4 (27%)	1 (7%)	8 (53%)	2 (13%)
Paed	***ChAdOx2 RabG***	45		38 (84%)	0 (0%)	0 (0%)	7 (16%)
Paed	***1-visit Verorab***	30		16 (53%)	2 (7%)	10 (33%)	2 (7%)
Paed	***2-visit Verorab***	30		28 (93%)	0 (0%)	1 (3%)	1 (3%)

Supplementary author list - the RAB002 study team

Rose Paul Mkumbange	Interventions and Clinical Trials Department, Ifakara Health Institute, Bagamoyo, Tanzania.
Ibrahim Sasamalo	Interventions and Clinical Trials Department, Ifakara Health Institute, Bagamoyo, Tanzania.
Aina-Ekisha Kahatano	Interventions and Clinical Trials Department, Ifakara Health Institute, Bagamoyo, Tanzania.
Safiness Daudi Mchome	Interventions and Clinical Trials Department, Ifakara Health Institute, Bagamoyo, Tanzania.
Bakari Mwalimu Bakari	Interventions and Clinical Trials Department, Ifakara Health Institute, Bagamoyo, Tanzania.
Neema Balige	Interventions and Clinical Trials Department, Ifakara Health Institute, Bagamoyo, Tanzania.
Hania Msami	Interventions and Clinical Trials Department, Ifakara Health Institute, Bagamoyo, Tanzania.
Mohamed Mohamed	Interventions and Clinical Trials Department, Ifakara Health Institute, Bagamoyo, Tanzania.
Egbert Stanslaus Kenya	Interventions and Clinical Trials Department, Ifakara Health Institute, Bagamoyo, Tanzania.

Supplementary references

1. World Organisation for Animal Health. Terrestrial Manual Chapter 2.1.17. Rabies (infection with rabies virus and other lyssaviruses). 2018. https://www.woah.org/fileadmin/Home/eng/Health_standards/tahm/2.01.17_RABIES.pdf.
2. Jenkin D, Ritchie AJ, Aboagye J, et al. Safety and immunogenicity of a simian-adenovirus-vectored rabies vaccine: an open-label, non-randomised, dose-escalation, first-in-human, single-centre, phase 1 clinical trial. *Lancet Microbe* 2022; **3**(9): e663-e71.
3. Clutterbuck EA, Lazarus R, Yu LM, et al. Pneumococcal conjugate and plain polysaccharide vaccines have divergent effects on antigen-specific B cells. *J Infect Dis* 2012; **205**(9): 1408-16.
4. O'Connor D, Clutterbuck EA, Gibani MM, et al. Prediction and characterisation of the human B cell response to a heterologous two-dose Ebola vaccine. *Nat Commun* 2025; **16**(1): 6331.
5. Folegatti PM, Bellamy D, Roberts R, et al. Safety and Immunogenicity of a Novel Recombinant Simian Adenovirus ChAdOx2 as a Vectored Vaccine. *Vaccines* 2019; **7**(2).
6. De Pijper CA, Langedijk AC, Terryn S, et al. Long-term Memory Response After a Single Intramuscular Rabies Booster Vaccination 10-24 Years After Primary Immunization. *J Infect Dis* 2022; **226**(6): 1052-6.
7. Langedijk AC, De Pijper CA, Spijker R, Holman R, Grobusch MP, Stijnis C. Rabies Antibody Response After Booster Immunization: A Systematic Review and Meta-analysis. *Clin Infect Dis* 2018; **67**(12): 1932-47.
8. Overduin LA, Koopman JPR, Prins C, et al. Boostability after single-visit pre-exposure prophylaxis with rabies vaccine: a randomised controlled non-inferiority trial. *The Lancet Infectious diseases* 2024; **24**(2): 206-16.
9. Soentjens P, De Koninck K, Tsoumanis A, et al. Comparative Immunogenicity and Safety Trial of 2 Different Schedules of Single-visit Intradermal Rabies Postexposure Vaccination. *Clin Infect Dis* 2019; **69**(5): 797-804.
10. Suwansrinon K, Wilde H, Benjavongkulchai M, et al. Survival of neutralizing antibody in previously rabies vaccinated subjects: a prospective study showing long lasting immunity. *Vaccine* 2006; **24**(18): 3878-80.

Study Title: A Phase Ib/II age de-escalation, dose escalation, partially randomised, open-label head-to-head study of the safety and immunogenicity of the candidate rabies vaccine ChAdOx2 RabG

Internal Reference Number: RAB002

OxTREC Ref: 8-20

ClinicalTrials.gov Identifier: NCT04270838

Date: 06 February 2023

Protocol Version: 5.0

**Chief Investigator (UOXF):**	**Dr Alexander D. Douglas**<br>The Jenner Institute<br>Wellcome Centre for Human Genetics<br>University of Oxford<br>Roosevelt Drive<br>Oxford<br>United Kingdom<br>OX3 7BN
**Principal Investigator (IHI):**	**Dr. Ally Olotu**<br>Ifakara Health Institute<br>P.O. Box 74<br>Bagamoyo,<br>Tanzania
**Sponsor:**	University of Oxford
**Funder:**	UK Medical Research Council
**Protocol Authors:**	Daniel Jenkin, Adam Ritchie, Ally Olotu, Alexander D. Douglas

Confidentiality Statement

This document contains confidential information that must not be disclosed to anyone other than the trial Sponsor, the Investigator Team, and members of the Ethics Committees and Regulatory Authorities. This information cannot be used for any purpose other than the evaluation or conduct of the clinical investigation without the prior written consent of the principal investigator.

Signature Page

**Chief Investigator (UOXF):**	**Dr Alexander D. Douglas**

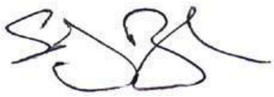

Signed______________________________________

Conflict of interest Statement: Dr Alexander Douglas is a named inventor on a patent application relating to the ChAdOx2 adenovirus serotype and would share in any income resulting from the patent.

**Principal Investigator (IHI):**	**Dr Ally Olotu**

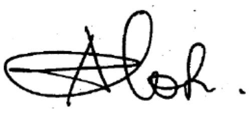

Signed______________________________________

Conflict of interest statement: None

I have read this protocol, and I agree to abide by all provisions set forth herein. I agree to comply with the principles of the International Conference on Harmonization Tripartite Guideline on Good Clinical Practice (GCP).

TABLE OF CONTENTS

1 SYNOPSIS 7
2 ABBREVIATIONS 9
3 BACKGROUND AND RATIONALE 11
3.1 Rabies 11
3.2 Impact of rabies 11
3.2.1 Local Rabies impact 11
3.3 Current rabies control and vaccination 11
3.3.1 Pre-exposure prophylaxis (PrEP) 12
3.3.2 Post-exposure prophylaxis (PEP) 12
3.3.3 Animal vaccination 12
3.4 The need for a new vaccine 13
3.5 Rabies glycoprotein as a vaccine antigen 13
3.6 Adenovirus-vectored vaccines 13
3.7 ChAdOx2 14
3.8 Development of ChAdOx2 vaccine vector 15
3.9 Development of ChAdOx2 RabG 16
3.10 Preclinical studies 16
3.11 Previous clinical experience 16
3.12 Rationale 19
3.13 Vaccine development strategy 21
3.14 Vaccination Services 22
4 OBJECTIVES AND OUTCOME MEASURES 23
4.1 Objectives 23
4.2 Outcome Measures 23
4.3 Time point(s) of evaluation 23
5 TRIAL DESIGN 24
5.1 Summary of trial design 24
5.2 Rationale for Trial Design 25
5.2.1 Rationale for ChAdOx2 RabG Route & Dose 25
5.2.2 Rationale for Comparator IRV Vaccine Route & Dose 26
5.2.3 Rationale for extended follow-up and simulated post-exposure prophylaxis. 27
5.2.4 Rationale for age de-escalation approach 28
5.2.5 Rationale for exclusion of participants who have received an adenovirus vectored vaccine within 6 months prior to enrolment, and advice to avoid other vaccines for 28 days after enrolment. 28
5.3 Safety oversight 29
5.3.1 UK Safety Data Reviews by National Ethical and Regulatory Authorities 29
5.3.2 Scheduled SMC and CI/PI data reviews during age de-escalation and dose escalation 30
5.3.3 Dose reduction in the event of increased reactogenicity 31
5.3.4 Holding Rules 32

5.3.5 Sentinel Participants 33
5.4 Trial Centre 33
6 PARTICIPANT IDENTIFICATION AND RECRUITMENT 34
6.1 Study Population 34
6.2 HIV Prevalence 34
6.3 Sensitization 34
6.4 Inclusion Criteria 35
6.5 Exclusion Criteria 36
6.6 Contraindications to subsequent vaccination 37
6.6.1 Indications for deferral of vaccination 37
6.6.2 Permanent Contraindications for further vaccination 38
6.6.3 Withdrawal of study participant 38
6.6.4 Replacement of Individual Participants after Withdrawal 39
6.7 Pregnancy 39
6.8 Managing volunteers during COVID-19 outbreak 39
7 TRIAL PROCEDURES 42
7.1 Informed Consent 42
7.2 Screening and Eligibility Assessment 42
7.2.1 Screening Visit 1 (SC1) 43
7.2.2 Screening Visit 2 (SC2) 44
7.3 Baseline Assessments 44
7.4 Allocation / randomisation procedure 44
7.5 Detailed scheduled study visits 45
7.6 Unscheduled Visits: 50
7.7 Sample Handling 50
7.8 Laboratory Evaluations 52
7.8.1 Safety Blood Assessments 52
7.8.2 Urinalysis: 52
7.8.3 Malaria Microscopy: 52
7.8.4 Serology 52
7.8.5 Urine Sample Pregnancy Test 52
7.9 Rationale for blood volume collected 53
7.10 Research Assays to Address Secondary endpoints 53
7.11 Study termination 54
8 INVESTIGATIONAL MEDICINAL PRODUCT (IMP) 55
8.1 IMP Description 55
8.1.1 ChAdOx2 RabG 55
8.1.2 Inactivated rabies vaccine 55
8.2 Storage of IMP 56
8.3 Accountability of the Investigational Product 56

8.4 Concomitant Medication 56
8.5 Post-trial treatment 57
9 SAFETY REPORTING 58
9.1 Definitions 58
9.2 Causality 59
9.3 Procedures for Recording Adverse Events 60
9.3.1 Recording adverse events 60
9.3.2 Solicited Systemic AEs 60
9.3.3 Solicited Local AEs: 61
9.3.4 Unsolicited AEs 61
9.3.5 Follow-up of Adverse Events 62
9.3.6 Grading the severity of adverse events 62
9.4 Reporting Procedures for Serious Adverse Events 63
9.4.1 Reporting Procedures for SUSARs 63
9.5 Safety Monitoring Committee 64
9.6 Development Safety Update Reports 65
10 STATISTICAL ANALYSIS CONSIDERATION 66
11 DATA HANDLING AND RECORD KEEPING 67
11.1 Data handling and record keeping 67
11.2 Access to Data 67
11.3 Source document and Case Report Form 67
12 QUALITY ASSURANCE PROCEDURES 69
12.1 Monitoring 69
12.2 Investigator procedures 69
12.3 Modification to protocol 69
12.4 Protocol deviation 69
12.5 Audit & inspection 70
12.6 Study results feedback 70
13 ETHICAL AND REGULATORY CONSIDERATIONS 71
13.1 Declaration of Helsinki 71
13.2 Guidelines for Good Clinical Practice 71
13.3 Approvals 71
13.4 Reporting 71
13.5 Participant Confidentiality 71
13.6 Potential Benefits to participants 72
13.7 Potential Risks and Burden to Participants 72
13.7.1 Vaccination: 72
13.7.2 Phlebotomy: 73
13.8 Incentives 73

13.9 Future use of stored samples 73
14 FINANCE AND INSURANCE 74
14.1 Funding 74
14.2 Insurance 74
15 PUBLICATION POLICY 75
16 REFERENCES 76
17 APPENDIX A: SCHEDULE OF PROCEDURES 80
18 APPENDIX B: SAE REPORTING FLOW CHART 82
19 APPENDIX C: AMENDMENT HISTORY 83
20 APPENDIX D: KEY ROLES AND GENERAL INFORMATION 87

1 SYNOPSIS

**Trial Title**	A Phase Ib/II age de-escalation, dose escalation, partially randomised, open-label head-to-head study of the safety and immunogenicity of the candidate rabies vaccine ChAdOx2 RabG.
**Study Reference**	RAB002
**Trial Registry**	ClinicalTrials.gov Identifier: NCT04270838
**Clinical Phase**	Phase Ib/II
**Trial Site**	Ifakara Health Institute Clinical Trial Facility, Tanzania

Trial Design:

Age de-escalation, dose-escalation, open label, head-to-head partially randomised trial. The study will consist of the groups as shown below:

Groups	Age at enrolment	n	Vaccination at enrolment
**AC1**	18-45 years	3	2.5×10^{10} vp ChAdOx2 RabG
**AC2**	18-45 years	12	5×10^{10} vp ChAdOx2 RabG
**AC3**	18-45 years	33 or 42	Adult preferred dose ChAdOx2 RabG*
**AV1**	18-45 years	15	Verorab<br>(2 site ID single visit)
**PC1a**	2-6 years	3	1×10^{10} vp ChAdOx2 RabG
**PC1b**	2-6 years	3	Half adult preferred dose ChAdOx2 RabG*
**PC2**	2-6 years	12	Full adult preferred dose ChAdOx2 RabG*
**PC3**	2-6 years	33 or 42	Paediatric preferred dose ChAdOx2 RabG*
**PV1**	2-6 years	30	Verorab (2 site ID single visit)
**PV2**	2-6 years	30	Verorab (2 site ID x **2 visits day 0 & 7**)

A= adult, P = paediatric, C1 = low dose ChAdOx2 RabG sentinel groups, C2 = high dose ChAdOx2 RabG n=12 tolerability assessment, C3 = preferred dose ChAdOx2 RabG, bringing the total to n=45 at that dose, V1 = single-visit Verorab, V2 = two-visit Verorab

*Section 5.3 describes the process of review of data from the preceding groups by Safety Monitoring Committee, CI & PI which will govern dose escalation in each age group, and the selection of the adult preferred dose and paediatric preferred dose. It is anticipated that the adult preferred dose will be 2.5×10^{10} vp or 5×10^{10} vp, and the paediatric preferred dose will be 50-100% of the adult preferred dose. The design ensures that 45 adults will receive the adult preferred dose, and 45 children will receive the paediatric preferred dose.

All participants will receive Verorab at 2 visits with the first of these designated 'day SPEP+0'. Day SPEP+0 may vary for different individuals within a permitted window, 273-546 days after enrolment (i.e., 9-18 months), at the investigators' discretion (as described in section 7.5).

- On day SPEP+0, the participant will receive 0.1mL Verorab ID at each of 4 sites as 'simulated post-exposure prophylaxis'

• On day SPEP+14, the volunteer will receive 0.1mL Verorab ID at each of 2 sites, and follow-up will cease.<br><br>All volunteers will thus receive Verorab on at least 2 visits, constituting robust pre-exposure prophylaxis independent of the vaccination given at enrolment.		
**Study Participants**	Healthy adults (18-45 years) and young children (2-6 years) residing in Bagamoyo, Tanzania. Up to 192 participants will be enrolled.	
**Follow up duration**	Up to 5.5 years.	
**Planned Trial Period**	Up to 6.5 years after the start of recruitment	
	**Objectives**	**Outcome Measures**
**Primary**	To determine safety and tolerability of ChAdOx2 RabG in healthy adults (18-45 years) and children (2-6 years) residing in a rabies endemic country.	• Local and systemic solicited AEs<br>• Unsolicited AEs<br>• Laboratory AEs<br>• Post vaccination serious adverse events during the study period.
**Secondary**	To assess immunogenicity of ChAdOx2 RabG and single-visit 2-site intradermal vaccination with licensed rabies vaccine in adults and children, in a rabies endemic country	• Rabies virus neutralising antibody as assessed by rapid fluorescent focus inhibition test (RFFIT)
**Exploratory**	To assess other anti-rabies antibody titres measured by ELISA, T cell responses measured by ELISpot and other assays.	• Cellular and humoral immunity to rabies antigen
**Investigational Medicinal Product(s)**	• ChAdOx2 RabG<br>• VERORAB (1 dose vial), a WHO pre-qualified inactivated rabies vaccine (IRV)	
**Route of Administration**	ChAdOx2 RabG will be given by intramuscular injection to the deltoid area of the non-dominant arm.<br>Licensed rabies vaccine will be given by intradermal injection at two to four anatomical sites (deltoids, thighs or suprascapular areas), as specified for particular visits.	

2 ABBREVIATIONS

ALT	Alanine Aminotransferase
ASC	Antibody Secreting Cells
BCG	Bacillus Calmette–Guérin
BDH	Bagamoyo District Hospital
BMI	Body Mass Index
CBF	Clinical Biomanufacturing Facility
CHW	Community Health Worker
CMI	Cell Mediated Immunity
CRF	Case Report form
CRO	Clinical Research Organization
CTA	Clinical Trial Agreement
CTF	Clinical Trial Facility
DNA	Deoxyribonucleic acid
DPT	Diphtheria, Pertussis and Tetanus.
EDC	Electronic Data Capture
EPI	Expanded Program of Immunization
GCP	Good Clinical Practice
HIV	Human Immunodeficient Virus
HRA	Health Research Authority
ICF	Informed Consent Form
ICS	Informed Consent Sheet
IFN	Interferon
IHI	Ifakara Health Institute
IRB	Institutional Review Board
IRV	Inactivated Rabies Vaccine
ISM	Independent Safety Monitor
IVD	Immunization and Vaccine Development
KEMRI	Kenya Medical Research Institute
MRC	Medical Research Council
NatHREC	National Health Research Ethics Sub-Committee (NatHREC)
OPV	Oral Polio Virus
OXTREC	Oxford Tropical Research Ethics Committee
PBMC	Peripheral blood mononuclear cells
PEP	Post-exposure prophylaxis
PIS	Participant Information Sheet
PrEP	Pre-exposure prophylaxis
RBC	Red Blood Cells
RSV	Respiratory Syncytial Virus
SAE	Serious Adverse Event
SAR	Serious Adverse Reaction
SDV	Source Data Verification
SMC	Safety Monitoring Committee

SOP	Standard Operating Procedures
SPEP	Simulated post-exposure prophylaxis
TMDA	Tanzanian Medicines and Medical Devices Authority
TMF	Trial Master File
TSG	Oxford University Hospitals NHS Foundation Trust / University of Oxford Trials Safety Group
UOXF	University of Oxford
WHO	World Health Organization

3 BACKGROUND AND RATIONALE

3.1 Rabies

Rabies is a neglected tropical disease caused by an enveloped, single-stranded RNA virus of the *Rhabdoviridae* family. The rabies virus (RABV) genome consists of five genes encoding glycoprotein, matrix protein, nucleoprotein, phosphoprotein, and the viral RNA polymerase [1, 2].

RABV has a wide host-range and spreads via the saliva of an infected animal coming into contact with a wound or mucosal surface. The virus spreads primarily though the peripheral nervous system to the central nervous system, where the most significant symptoms leading to death occur. The incubation period ranges from several days to several years in humans, with 2-3 months being most common. Without vaccination, infection results in acute encephalitis and death, although treatment with post-exposure prophylaxis (PEP) is highly effective if it occurs soon after exposure [2, 3].

3.2 Impact of rabies

The belief that rabies is a bygone disease for which an adequate vaccine exists is incorrect. The annual, global human impact of rabies is approximately 59,000 deaths, the loss of 3.7 million disability-adjusted life years (DALYs) and US$ 8.6 billion in economic costs. The greatest burden of impact is felt in Asia and Africa, where 95% of deaths occur [2, 4].

Around 99% of human exposures to rabies is through dog bites, although transmission via bats is the primary mode of transmission in the Americas. Following potential exposure, the cost of PEP treatment represents 31 days' wages for the average Asian and 51 days wages for the average African [2]. Even when costs are not prohibitive to individuals, PEP is often unavailable in local clinics when needed.

3.2.1 Local Rabies impact

Rabies is responsible for c. 1500 deaths per year in Tanzania, around half of which occur in children [5]. Access and adherence to PEP is variable and incomplete [6].

3.3 Current rabies control and vaccination

Rabies control is multifaceted, including pre-exposure prophylaxis (PrEP) and PEP in humans, and vaccination of animal reservoirs.

The first rabies vaccine was developed in the 19th century by Pasteur, making it the second human disease for which a vaccine was successfully developed. Recommended current vaccines are produced using cell culture systems and are used for both PrEP and PEP. In

some countries vaccines are still produced using mammalian nervous system tissue, which the WHO strongly recommends against [2].

The WHO released a new position paper on the use of rabies vaccines in April 2018 [7, 8]. This included the recommendation of the greater use of intradermal (ID) over intramuscular (IM) administration and new regimens for PrEP and PEP. These changes are primarily aimed at decreasing costs and improving access and adherence, while maintaining comparable levels of protection.

3.3.1 Pre-exposure prophylaxis (PrEP)

PrEP usually involves 3 doses of vaccine spread over several weeks. The new WHO guidelines suggest 2 doses of vaccine, administered either ID or IM, spread over one week is sufficient for protection [8]. Experience with single-visit regimes with existing licensed vaccines is limited but available evidence suggests that a substantial proportion of recipients of such regimes fail to sero-convert and antibody titer maintenance is relatively poor [9-11].

3.3.2 Post-exposure prophylaxis (PEP)

For individuals at high risk of rabies exposure that have not been previously vaccinated, current WHO guidelines suggest 3-4 doses of vaccine spread over 1-3 weeks, with RIG for the most serious level of exposure [8]. In practice RIG is often unavailable, or excluded from local recommendations, largely on grounds of cost.

For individuals at high risk of rabies exposure who have been previously vaccinated, PEP involves 2 doses of vaccine 3 days apart, or 4-site ID administration at a single visit, and no RIG [8, 12, 13].

3.3.3 Animal vaccination

The major vectors are dogs in Asia and Africa. Unfortunately, many parenteral dog vaccination campaigns fail to reach adequate levels of coverage [14]. Dog vaccination has been endorsed by the Global Alliance for Rabies Control and enshrined in the Zero by Thirty strategy as the preferred route to reduce human rabies mortality [15, 16]. However, funding for this approach remains poor and many regions lack – and will likely continue to lack – the animal health infrastructure to implement this.

3.4 The need for a new vaccine

These existing control measures are inadequate: current human vaccines are too expensive for mass use, and canine vaccination is challenging in many settings.

Existing inactivated-virus human rabies vaccines require expensive manufacturing processes, repeated dosing and, with one exception, cold-chain storage. They are not cost-effective for PrEP in low-income settings [17]. PEP is prohibitively expensive, representing 51 day's wages for the average African, and not promptly available in areas where most cases occur [2, 4, 17].

Despite the spread of canine vaccination as a method of control, with significant success in certain areas, global incidence is stable and, in some areas, rising. In many areas there is a lack of animal health infrastructure to achieve the necessary >70% coverage in dog populations that are highly mobile and turn over every 2 years. Rural settings, where dog populations are more dispersed, are particularly challenging. Yet in many such settings high rates of routine paediatric immunisation have been achieved, e.g. against measles, suggesting routine population-wide PrEP as a pathway to effectively reduce human rabies mortality in areas which struggle to implement canine vaccination.

A low cost, single dose, efficacious human rabies vaccine would thus be a major advance for rabies control. Rabies elimination would also require control in dogs and other reservoirs, but a new human vaccine has the potential to have a major impact on global mortality and economic costs arising from rabies.

3.5 Rabies glycoprotein as a vaccine antigen

Rabies glycoprotein (RabG) is the key surface antigen against which protective neutralising virus antibodies are induced by currently available vaccines [18]. Thus, millions of individuals have previously been safely administered this antigen. Vaccination with RabG via adenovirus vectors has been shown to induce RabG antibody titers comparable to currently available vaccines in animal models, and to protect non-human primates from rabies challenge [18, 19]. ChAdOx2 RabG contains the full length RabG sequence.

3.6 Adenovirus-vectored vaccines

Adenoviruses are attractive vectors for human vaccination. They possess a stable genome so that inserts of foreign genes intended for *in vivo* expression are maintained during repeated culture. They can enter a wide variety of cell types and their genetic material remains extra-chromosomal, meaning there is no potential for insertional mutagenesis in host cells. Replication incompetent adenovirus vectors have been engineered through the deletion of the E1 locus, ensuring there is no replication within vaccine recipients' cells. These viruses can still be propagated with high yields *in vitro*

using cell lines expressing the E1 locus, such as human embryonic kidney 293 cells (HEK 293 cells) [20].

Previous mass vaccination campaigns in over 2 million adult US military personnel using orally administered live human adenovirus (AdHu) serotype 4 and 7 have shown good safety and efficacy data [21]. AdHus are under development as vectors for malaria, HIV and hepatitis C vaccines, amongst others. They have been used extensively in human trials with a consistently excellent safety profile.

A limiting factor to widespread use of human adenovirus as vaccine vectors has been the level of pre-existing immunity, with seroprevalence of up to 90% reported in sub-Saharan Africa [22, 23]. This limits the immunogenicity of the AdHu vectored vaccines, with several animal models showing prior exposure to AdHu attenuates responses to subsequent challenge with AdHu vectored vaccines but not Chimpanzee adenovirus (AdC) vectored vaccines, including against rabies glycoprotein [24-26]. The ideal adenovirus vector would be replication deficient, not cause human disease, produce high yields during manufacture, and be highly immunogenic. AdCs that have these characteristics are increasingly accepted as widely applicable vectors for human use [27, 28].

Key to their applicability as vaccine vectors is that AdCs exhibit hexon structures that show enough homology to AdHu hexon to enable effective infection of human cells, while circumventing pre-existing immunity to the most common AdHu serotypes [27, 29]. Several trials have now shown excellent immunogenicity in a variety of unselected human populations, including children in low-income settings [30-32]. Moreover, the vectors have an excellent track record of safety and tolerability (drawn from experience in >1000 vaccinees with Oxford-developed vaccines alone). Industrial confidence in the platform is demonstrated by heavy investment by both Johnson & Johnson and GSK: both have purchased companies developing simian adenovirus vaccines, are involved in Phase II/III trials of adenovirus-based Ebola vaccines, and have invested in adenovirus manufacturing capabilities. Chimpanzee adenoviral vectors can be manufactured cost-effectively [33] and are now in clinical development as possible vaccines against malaria, HIV, tuberculosis, influenza, hepatitis C, RSV, cancer and Ebola.

3.7 ChAdOx2

ChAdOx2 is a replication-incompetent adenovirus vector derived from wild type AdC serotype C68 (AdC68) and ChAdOx1, a viral vector previously developed in the Jenner Institute. ChAdOx1 is described by Dicks *et al.* [34] and has had a good safety profile in numerous clinical trials. ChAdOx1 was constructed in a bacterial artificial chromosome (BAC) to facilitate genetic manipulation of genomic clones with improved stability and flexibility. Cellular immunogenicity of ChAdOx1 was comparable to that of other species E derived chimpanzee adenovirus vectors including ChAd63, the first simian adenovirus vector to enter clinical trials in humans. ChAdOx2 was then produced in the same manner, starting from the replication-competent AdC68 with further modification to the E4 region to increase yields during manufacture [27]. As ChAdOx2 is replication incompetent, E4 is

not expressed in vaccine recipients' cells. This modified E4 region was derived from ChAdOx1 and AdHu5, both of which have a good safety profile in clinical trials.

Serotype Y25 (from which ChAdOx1 is derived), AdC68 (also known as SAdV25, and from which ChAdOx2 is derived), and ChAd63 are all species E adenoviruses and have a close phylogenetic relationship when nucleotide sequences of the hexon and fibre proteins are analysed (Figure 1).

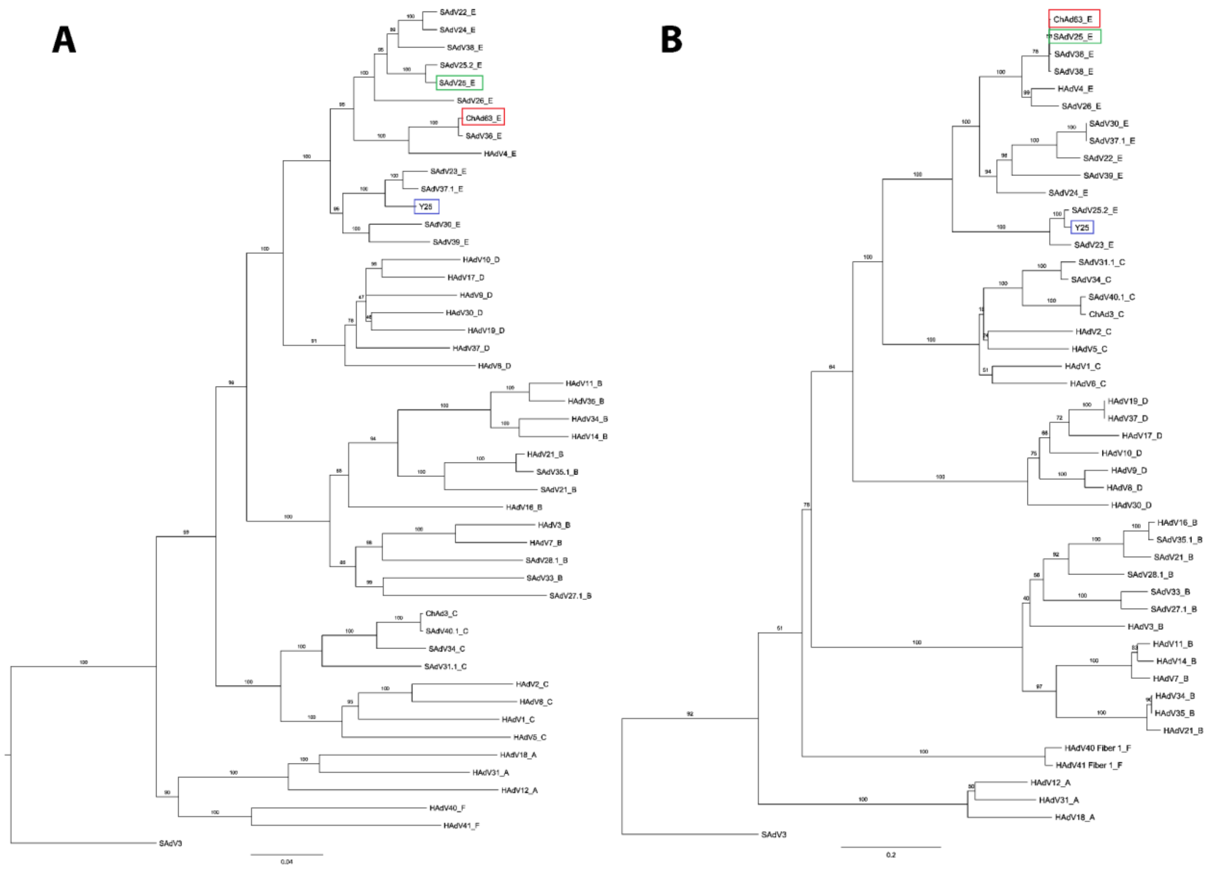

*Figure 1. Phylogenetic trees based on alignment of nucleotide sequences of (A) hexon and (B) the fibre protein of different human and chimpanzee adenovirus serotypes. AdC68 (SAdV25; green), Y25 (blue) and ChAd63 (red) are highlighted. Modified from **[34]**.*

3.8 Development of ChAdOx2 vaccine vector

To generate a molecular clone of the AdC68 genome, a BAC gap repair vector was constructed containing PCR-amplified regions of homology to the left and right flanks of the viral genome as described in Chartier *et al* [35]. An extra homology flank downstream of the adenovirus E1 region was included to enable deletion of E1 and placement of a unique restriction site at the E1 locus, concomitant with genomic insertion into the BAC. The E1 region is essential for viral replication, hence the ability to delete E1 at this stage renders the new vector immediately replication incompetent. Replication incompetent (E1-deleted) clones were successfully identified by PCR screening and transfection into E1 complementing HEK293 cells confirmed the ability of all candidate clones of the new vector

to generate infectious virions. The non-essential E3 region was subsequently deleted to increase the insertion capacity of the vector.

Proteins encoded by the E4 region interact with E1 proteins during viral replication, and the imperfect interaction between the gene products of the AdHu5 E1 gene produced by HEK293 cells and simian E4 gene products has been found to result in impaired viral replication in this cell line, and consequently lower virus yields [29, 34, 36]. In ChAdOx1, Ad5 E4Orf4,6/7 have been inserted to replace the homologous simian virus coding sequence, resulting in improved viral replication during vaccine production [34]. Since no replication of the virus takes place after immunization, this replacement has no effect on immunogenicity of the viral vector. In the construction of ChAdOx2, the whole of the native AdC68 E4 region was replaced with the equivalent regions from ChAdOx1.

3.9 Development of ChAdOx2 RabG

ChAdOx2 RabG encodes the rabies glycoprotein. A genomic clone of ChAdOx2 RabG was prepared by Gateway® recombination between an entry plasmid containing the native coding sequence for glycoprotein from the ERA strain rabies virus under the transcriptional control of an Intron-A containing human cytomegalovirus immediate-early promoter, and the E1-and E3-deleted ChAdOx2 destination vector.

3.10 Preclinical studies

Studies have shown ChAdOx2 RabG to be immunogenic, inducing both antibody [18] and T cell responses in mice. Full details are available in the current version of the ChAdOx2 RabG Investigator's Brochure (IB).

In a non-human primate trial, an AdC68 vectored RabG vaccine outperformed the current human rabies vaccine: a single dose protected 100% of animals challenged 22 months after vaccination [19].

3.11 Previous clinical experience

The first-in-human clinical trial of ChAdOx2 RabG (RAB001) is being carried out at Oxford in the UK from 2019 to 2023. The first 12 participants in the RAB001 trial have now completed the study. A further 6 participants have also received a 5×10^{10} vp dose of ChAdOx2 RabG and are in the follow up period (Table 1). Further information is available in the current version of the IB. This Tanzanian trial is the second clinical trial of ChAdOx2 RabG, and commenced after establishment of a good safety profile in the RAB001 trial (see section 5.3 for pre-specified review process). The gene product of the antigenic insert, RabG, is present in several currently licensed rabies vaccines, and thus has been administered successfully to millions of individuals.

Another ChAdOx2 vectored vaccine expressing a different insert has been administered to 22 healthy volunteers taking part in a clinical trial in the UK conducted by the University

of Oxford [37]. This vaccine was successfully administered at the same doses used in the RAB001 study, and there were no serious adverse events (SAEs) associated with the vaccine reported to date. The number of participants and doses are outlined in Table 1.

***Table 1**. University of Oxford clinical experience with ChAdOx2 viral vectored vaccines.*

Country	Trial	Vaccine	Age	Route	Dose	Number of Volunteers Received ChAdOx2 (up to 1 Oct 2019)
UK	RAB001	ChAdOx2 RabG	21-63	IM	5×10^{9} vp	3
					2.5×10^{10} vp	3
					5×10^{10} vp	12
UK	HAV001	ChAdOx2 HAV	18-50	IM	5×10^{9} vp	3
					2.5×10^{10} vp	3
					5×10^{10} vp	16

Chimpanzee adenovirus vaccine vectors have been safely administered in non-COVID-19 clinical trials to over 1000 people, targeting a wide range of pathogens (and also cancer). ChAdOx1 is a chimpanzee adenovirus vaccine vector system that has been administered to over 200 healthy volunteers taking part in non-COVID-19 clinical trials conducted by the University of Oxford. The vaccine studies, doses, and number of participants are outlined in Table 2.

Beginning in 2020, ChAdOx1 nCOV-19 has been administered to tens of thousands of participants in clinical trials. It was subsequently approved for emergency use by UK, European and WHO regulators, using a regimen of two intramuscular doses. Billions of doses have been delivered around the world, and there is now extensive clinical experience of its use. Common side effects include pain, feeling feverish, chills, muscle ache, headache, and malaise. These effects appear to be less pronounced in recipients in older age groups. Very rare but serious and in some cases fatal adverse reactions have also occurred, including a syndrome of vaccine-induced thrombosis with thrombocytopenia (VITT). These_adverse effects are discussed further in the ChAdOx2 RabG IB. Given the rarity of these events we believe the risk: benefit balance of participation in RAB002 is strongly positive (see section 3.12).

ChAdOx1 and ChAdOx2 are derived from closely related species E chimpanzee adenoviruses, modified in similar ways and sharing a region of non-structural gene sequence (see section 3.7 and Figure 1), making ChAdOx1 safety data relevant to consideration of ChAdOx2.

ChAd63 is another chimpanzee adenovirus vaccine vector system successfully and safely administered to human participants in a range of clinical trials [38]. The University of Oxford has a wide range of experience using this vector in clinical trials in collaboration with several different trial sites in Africa as shown in Table 3. ChAd63 is also a species E chimpanzee adenovirus closely related to the virus from which ChAdOx2 RabG is derived (see section 3.7 and Figure 1).

***Table 2**. University of Oxford clinical experience with ChAdOx1 viral vectored vaccines.*

Country	Trial	Vaccine	Age	Route	Dose	Number of Volunteers (Received ChAdOx1 up to 1 May 2019)
UK	FLU004	ChAdOx1 NP+M1	18-50	IM	5×10^{8} vp	3
					5×10^{9} vp	3
					2.5×10^{10} vp	3
					5×10^{10} vp	6
UK	FLU005	ChAdOx1 NP+M1<br>MVA NP+M1 (week 8)	18-50	IM	2.5×10^{10} vp	12
		ChAdOx1 NP+M1<br>MVA NP+M1 (week 52)	18-50	IM	2.5×10^{10} vp	12
		MVA NP+M1<br>ChAdOx1 NP+M1 (week 8)	18-50	IM	2.5×10^{10} vp	12
		MVA NP+M1<br>ChAdOx1 NP+M1 (week 52)	18-50	IM	2.5×10^{10} vp	9
		ChAdOx1 NP+M1	>50	IM	2.5×10^{10} vp	12
		ChAdOx1 NP+M1<br>MVA NP+M1 (week 8)	>50	IM	2.5×10^{10} vp	12
UK	TB034	ChAdOx1 85A	18-50	IM	5×10^{9} vp	6
					2.5×10^{10} vp	12
		ChAdOx1 85A<br>MVA85A (week 8)	18-50	IM	2.5×10^{10} vp	12
UK	VANCE01	ChAdOx1.5T4<br>MVA.5T4	18-75	IM	2.5×10^{10} vp	34
UK	VAC067	ChAdOx1 LS2	18-45	IM	5×10^{9} vp	3
					2.5×10^{10} vp	10
UK	MERS001	ChAdOx1 MERS	18-50	IM	5×10^{9} vp	6
					2.5×10^{10} vp	9
					5×10^{10} vp	9
UK	CHIK001	ChAdOx1 Chik	18-50	IM	5×10^{9} vp	6
					2.5×10^{10} vp	9
					5×10^{10} vp	9
UK	VAMBOX	ChAdOx1 MenB.1	18-50	IM	$2.5x10^{10}$ vp	3
					$5x10^{10}$ vp	24
Switzerland	TB039	ChAdOx1 85a	18-55	IM / aerosol inhaled	$5x10^{9}$ vp (IM)	6
					$2.5x10^{10}$ vp (IM)	36
					$1x10^{9}$ vp (inhaled)	3
					$5x10^{9}$ vp (inhaled)	3
Uganda	TB042	ChAdOx1 85a	12-17<br>18-49	IM	$5 x10^{9}$ vp<br>$2.5 x10^{10}$ vp	12

***Table 3** University of Oxford International clinical experience with ChAd63 vectored vaccines*

Country	Trial	Vaccine	Age	Route	Dose	Number of Volunteers Vaccinated (up to May 2019)
Kenya	VAC040	ChAd63 ME-TRAP	18-50 yrs (male only)	IM	1 x 10^{10} vp, 5 x 10^{10} vp	30
Gambia	VAC041 (Groups A,B)	ChAd63 ME-TRAP	18-50 yrs (male only)	IM	1 x 10^{10} vp, 5 x 10^{10} vp	16
Gambia	VAC041 (Groups 2A-2C, 3A-3C)	ChAd63 ME-TRAP	2-6 yrs	IM	1 x 10^{10} vp, 5 x 10^{10} vp	24
Gambia	VAC042	ChAd63 ME-TRAP	5-12 months, 10 weeks	IM	1 x 10^{10} vp, 5 x 10^{10} vp	48
Gambia	VAC058	ChAd63 ME-TRAP	1-12 weeks	IM	5 x 10^{10} vp	45
Kenya	VAC046	ChAd63 ME-TRAP	18-50 yrs (male only)	IM	5 x 10^{10} vp	61
Senegal	VAC047	ChAd63 ME-TRAP	18-50 yrs (male only)	IM	5 x 10^{10} vp	60
Burkina Faso	VAC050	ChAd63 ME-TRAP	5-17 months	IM	5 x 10^{10} vp	380
Tanzania	VAC070	ChAd63 Rh5	18-35 yrs, 1-6 yrs, 6-11 months	IM	1 x 10^{10} vp 5 x 10^{10} vp	63

3.12 Rationale

Rabies causes significant mortality and hardship globally, particularly in low- and middle-income countries where it is endemic. Although both pre- and post-exposure prophylaxis is available, costs for both are too high, which coupled with the need for multiple visits, prevents their full potential being realised. Pre-exposure prophylaxis is too expensive for large-scale vaccination campaigns that have been used to combat other diseases like measles. Post-exposure prophylaxis carries significant out of pocket expenses for many people that lead to it being unaffordable or causing financial stress. ChAdOx2 RabG has the potential to lower costs and make mass vaccination campaigns realistic.

Chimpanzee adenovirus vaccine vectors have been safely administered to thousands of people using a wide range of infectious disease targets. ChAdOx2 viral vectored vaccines have shown to be both safe and immunogenic in a previous clinical trial (HAV001) [39]. Single-dose immunisation with the ChAdOx2 RabG vaccine has been shown to elicit high levels of neutralising antibody in animal models.

The RAB002 study will provide valuable data on safety, immunology, and longevity of responses against RabG following vaccination with ChAdOx2 RabG in adult and pediatric populations from a rabies endemic region.

The original protocol for RAB002 was developed prior to the COVID-19 pandemic, and with very limited prior information from the RAB001 Phase I study of ChAdOx2 RabG in the UK. Revisions to the RAB002 study design have therefore been made, both in light of the availability of additional data from RAB001 and in light of experience gained from the development and global use of ChAdOx1 nCoV-19.

As of November 2022, the RAB001 study in the UK has been extended through an amendment to increase the numbers of participants receiving a full adult dose of ChAdOx2 RabG (5×10^{10} vp). Results from the first 12 participants have surpassed expectations, with all volunteers in the $2.5x10^{10}$ VP group (n=3) and $5x10^{10}$ VP group (n=6) achieving VNA titers >0.5 IU/mL (the accepted serological marker of satisfactory response to rabies vaccination) by day 56 after a single dose. 6/7 who have reached the one-year follow-up point had maintained VNA >0.5 IU/mL at this point, and the one participant who had dropped below this level achieved high VNA levels again within 7 days of receiving simulated PEP.

Data from RAB001 encouraged expansion of the RAB002 study (through SA001) from the previously approved Phase Ib design (n=48) to a larger Phase Ib/II design (n=192) which will enable collection of additional data on safety, immunogenicity and in particular longevity of induced immune responses in Tanzanian adults and children. Enhancements to the design at that time included:

- **Dose titration for both adults and children**. UK immunogenicity was good at both $2.5x10^{10}$ VP and $5x10^{10}$ VP, and although the safety profile was very good, reactogenicity (in small numbers) at the upper dose was towards the upper end of tolerability for a product administered to healthy individuals (for further details please see IB). The revised RAB002 design allows for selection of well-tolerated adult and paediatric preferred doses, informed by data obtained in Tanzania and hence suitable for the Tanzanian population.

- **Larger numbers of adult and paediatric participants receiving ChAdOx2 RabG,** permitting collection of a larger volume of safety & immunogenicity data.

Creation of flexibility to extend follow-up of subsets of adults and children for extended period, up to 5 years.

- **Inclusion of additional comparator groups.** The previous design only included one comparator group (children receiving an IRV at a single visit at baseline). The revised design will also allow immunogenicity of ChAdOx2 RabG to be compared to that of single-visit IRV vaccination in adults, and to two-visit IRV vaccination in children. We consider single-visit IRV to be the most relevant comparator for single-visit ChAdOx2 RabG, both on scientific grounds and because we believe single-visit IRV is probably the most cost-effective approach to PrEP using currently licensed vaccines. Single-visit IRV PrEP is however unlicensed and recommended by WHO only when two-visit PrEP is not possible. Two-visit IRV PrEP (days 0 and 7) is therefore now included as a WHO-recommended gold standard. As previously, all participants will be offered a two-visit PrEP regime at the end of their participation in the study.

As of November 2022, initial data on safety and immunogenicity (up to day 56) for adult participants in this study (RAB002) trial is encouraging. For ChAdOx2 RabG,

immunogenicity is consistent with the immunogenicity seen in the UK in RAB001. Immunogenicity of single-visit Verorab is also encouraging. It is therefore desirable to amend the study (through SA003) to establish the duration of maintenance of protective levels of VNA for all regimes, including the recall response to simulated PEP. The revised design of the study will allow participants to opt in to a follow up period of up to 5.5 years. Any participants who do not opt in to this extended period of follow up will receive simulated PEP at 1 year as originally planned. Participants in the extended follow up period will have their levels of VNA measured regularly, and those who are found to drop below the protective level (0.5IU/mL) will be randomized to receive simulated PEP starting at either their next visit or at the 5 year visit.

Experience with ChAdOx1 nCoV-19 during the COVID-19 pandemic has been informative in a number of ways. Manufacturing of chimpanzee adenovirus vectored vaccines has advanced substantially, reinforcing the belief that this technology is suitable to achieve very low-cost rabies vaccination. The safety profile of the ChAdOx1 nCoV-19 vaccine is also now very well understood. It is unclear which of the rare serious adverse effects associated with ChAdOx1 nCoV-19 would also occur after vaccination with a different serotype of adenovirus, expressing a different antigen, in a genetically different population in a different environment (for further discussion see the IB).

We have reviewed the risk: benefit balance of use of ChAdOx2 RabG in the current study and believe this remains strongly favourable. In particular the incidence of vaccine-induced thrombosis with thrombocytopenia after administration of a single dose of ChAdOx1 nCov-19 in 18–49-year-olds in the UK is c. 1:49,000 (all cases) and c. 1:270,000 (fatal cases). In contrast lifetime risk of death due to rabies in Tanzania is of the order of 1:2000, with incidence highest in children [4]. As it is now clear that ChAdOx2 RabG induces protective antibody levels in UK participants in RAB001, and as all RAB002 participants will additionally have received effective PrEP with a licensed vaccine by the end of the study, the benefit to RAB002 participants in terms of reduction of rabies risk is likely to be many-fold greater than the risk of VITT.

Despite the rarity of VITT, the revised study design does contain measures to mitigate the already small risk, in particular exclusion of participants with risk factors predisposing to thrombosis, and platelet counts at days 7, 14 and 28 after vaccination.

3.13 Vaccine development strategy

The data from the RAB002 clinical trial will be used to support larger Phase II/III trials both in Tanzania and other countries where rabies is endemic.

3.14 Vaccination Services

The United Republic of Tanzania provides immunization services countrywide both on routine and non-routine bases. Immunization services are coordinated by the Immunization and Vaccine Development (IVD) services. This service is available at public and private facilities free of charge. 80% of the 5,650 health facilities in the country provide immunization services. Tanzanian immunization policy calls for support for routine immunization to accelerate the control of vaccine preventable diseases. The following vaccines are provided: BCG, OPV, DTP-HepB-Hib (pentavalent), pneumococcal, rotavirus, tetanus toxoid, Measles-Rubella and HPV. The immunization schedule in mainland Tanzania is shown in Table 4. Additionally, vitamin A supplementation is administered via the vaccination programme and takes place at 9, 15 and 21 months.

Table 4 Immunization Schedule in Tanzania.

Antigen	Time of administration
BCG, OPV 0	At birth or first contact
OPV1, DTP-HepB-Hib1,PCV 1, Rota 1	6 Weeks of age
OPV2, DTP-HepB-Hib2, PCV 2, Rota 2	10 Weeks of age
OPV3, DTP-HepB-Hib3 , PCV3, IPV	14 Weeks of age
TT 1	First contact
TT 2	1 month after 1st dose
TT 3	6 months after 2nd dose
TT 4	1 year after 3rd dose
TT 5	1 year after 4th dose
MR 1	9 Months of age
MR 2	18 Months of age
HPV 1	9 years
HPV 2	6 months after 1st dose

The Immunization and Vaccine Development (IVD) services are provided through four main strategies. The primary strategy is through routine immunization services at health facilities. The secondary strategies include outreach services to remote communities and mop-up campaigns aimed at selected districts to capture defaulters and reach out to children missed in routine services. Finally, there are occasional national vaccination campaigns which are implemented to reach large populations in a given period, as a supplementary activity to the routine immunization in order to increase the immunity in the community.

4 OBJECTIVES AND OUTCOME MEASURES

4.1 Objectives	4.2 Outcome Measures	4.3 Time point(s) of evaluation
**Primary Objective**<br>To assess the safety profile of ChAdOx2 RabG in healthy adult volunteers (18-45 years) and young children (2-6 years) residing in a rabies-endemic country.	• Solicited local reactogenicity signs and symptoms	D0 to D7
	• Solicited systemic reactogenicity signs and symptoms	D0 to D7
	• Unsolicited adverse events	D0 to D28
	• Clinical laboratory adverse events.	D0 to D28
	• Occurrence of serious adverse events	D0 until final follow up visit
**Secondary Objectives**<br>To assess immunogenicity of ChAdOx2 RabG administered to adults and young children residing in a rabies endemic country following primary vaccination, including length of response maintenance, and secondary (recall) response.<br><br>To compare immunogenicity of ChAdOx2 RabG with a single visit two site intradermal IRV PrEP regimen.	• Rabies virus neutralising antibody as assessed by rapid fluorescent focus inhibition test (RFFIT)	Data from multiple timepoints (D0, D28, D56, D186, D365, D730, D1095, D1460, D1825) will contribute to analyses specified by the SAP.
	• Anamnestic rabies virus neutralising antibody response as assessed by rapid fluorescent focus inhibition test (RFFIT), at 7 days following simulated rabies virus exposure through IRV administration	SPEP+0 and SPEP+7
**Exploratory Objectives**<br><br>Other rabies-specific exploratory immunology assays may be carried out.	• May include anti-rabies antibody titres measured by ELISA, T cell responses measured by ELISpot and other assays, and anti-adenovirus-vector antibody responses	Variable

5 TRIAL DESIGN

5.1 Summary of trial design

- Experimental design: Phase Ib/II, open-label, head-to-head, age de-escalation dose-escalation, partially randomized trial.
- Healthy adults (18-45 years) and children (2-6 years) will be screened; those determined to be eligible, based on the inclusion and exclusion criteria, will be enrolled in the study.
- Route of administration: ChAdOX2 RabG will be administered by the intramuscular route to the deltoid region of the non-dominant arm. Inactivated rabies virus (VERORAB) will be administered intradermally at 2-4 anatomical sites (as specified for particular visits) including: deltoids, thighs or suprascapular areas.
- Each participant will be observed for at least 1 hour after vaccination at day 0 to evaluate and treat any acute adverse events (AEs).
- There will be a 7-day follow-up period for collection of solicited AEs from the initial D0 vaccination. Day 0, 1 and 7 evaluations will be carried out at the study centre by the study clinician and day 2, 3, 4, 5 and 6 evaluations will be carried out by a trained community health worker in the participant's home, after each vaccination.
- There will be a 28-day (day of vaccination and 28 subsequent days) period after the day 0 vaccine dose for reporting unsolicited symptoms.
- Serious adverse events (SAEs) will be recorded throughout the study period. All SAEs will be captured in the period following the administration of the D0 vaccination until the final follow up visit. Prior to D0 vaccination, SAEs due directly to study procedures will also be captured.
- Rabies virus neutralisation antibody titres will be determined at baseline and days 28, 56, 365, 730, 1095, 1460, 1825, SPEP+0 and SPEP+7 depending on the period of follow up for each individual participant. For each participant, the SPEP+0 visit will replace one of either the day 365. 730, 1095, 1460 or 1825 visits.
- The duration of involvement in the study from enrolment will be up to 5.5 years. The primary vaccination phase of the study takes 1 week and the post-primary vaccination follow-up lasts for up to 5.5 years, at which stage participants will receive 2 doses of a VERORAB separated by 14 days.
- Participants will be invited to opt in to extended follow up of up to 5.5 years. Participants will have the VNA levels measured regularly, and those dropping below 0.5IU/mL randomised 1:1 to begin simulated PEP at either the next available visit or at the latest opportunity (14 days before the participant's last study visit, which will take place up to 5.5 years post-enrolment).
- Participants who do not opt in to extended follow up will be involved in the study for up to 570 days, as consented to under protocol versions up to v4.0.
- The study investigators may decide to end the extended period of follow up if, in their judgement, immune responses have dropped below protective levels in so many individuals that further follow up is not of scientific value.

- Data collection: conventional Case Report Form (CRF) followed by transcription into the OpenClinica Electronic Data Capture system.

5.2 Rationale for Trial Design

This section details rationale for specific trial design choices. For broader rationale relating to the development strategy, including modifications to the trial made in the light of experience from RAB001 and use of ChAdOx1 nCoV-19, please see section 3.12.

5.2.1 Rationale for ChAdOx2 RabG Route & Dose

Most clinical trials with adenovirus-vectored vaccines, including the RAB001 study, have used the IM route.

Pre-clinical studies using comparable doses to those selected for this trial, have demonstrated the long-term efficacy of chimpanzee adenovirus vectored rabies glycoprotein against rabies challenge when administered as a single intramuscular dose (see section 3.10). The upper end of the dose range selected for this trial ($5x10^{10}$ VP) is based on those used in previous ChAd vectored vaccine trials (involving adults and children) which are known to induce robust antibody and T-cell responses with minimal reactogenicity (see section 3.11). There are substantial data showing good safety and tolerability in African adults, children and infants who received the same dose of Chad63 (a highly similar vector) as in the upper end of the dose range planned for this study [40-43].

Both $2.5x10^{10}$ VP and $5x10^{10}$ VP doses were shown to have good safety profile in healthy UK adults, although there was a non-significant trend towards more reactogenicity with the high dose. Paracetamol would be administered by study clinician if there is fever during close monitoring of participants post-vaccination. With ChAdOx2 RabG itself in the UKWith ChAdOx2 RabG itself in the UK

We intend to select the maximum tolerable dose in a given age group as the preferred dose for the study, with an upper limit for adults of $5x10^{10}$ VP and an upper limit for children of the adult preferred dose. There would be no obvious benefit to use of a dose less than the maximum tolerable dose (the cost of drug substance is very low even at a dose of $5x10^{10}$ VP). Given the UK data, we do not expect doses above $5x10^{10}$ VP to have attractive tolerability.

The dose escalation strategy therefore aims to provide data to select between a preferred dose of $2.5x10^{10}$ VP or $5x10^{10}$ VP in adults, and a preferred dose of 50 or 100% of the adult preferred dose in children.

The dose-limiting reactogenicity for adenovirus vectors occurs in the 48h after administration, peaking at 24h [44], and consists of relatively mild systemic symptoms which occur commonly (for example, fatigue at grade 1-2 in ~55%, and grade 3 in ~5% of recipients of ChAdOx1 nCoV-19 without paracetamol). It is therefore possible to determine

from a small number of days of follow-up of a small group (e.g., 12) whether a given dose is likely to be tolerable in a larger cohort.

The dose-escalation strategy for this study, both for adults and children, therefore, provides small sentinel groups at lower doses before assessment of reactogenicity in 12 participants at the upper possible dose.

Based upon at least 7 days of follow-up of these groups, the CI and PI will select the preferred dose to be used for the remaining participants.

Stated doses are the maximum to be administered and may be reduced at the discretion of the investigators or SMC.

The investigators may provide prophylactic paracetamol for optional use for 24h after vaccination, if it is felt that this is likely to assist tolerability. With ChAdOx1 nCoV-19, paracetamol is routinely recommended and does not adversely affect immunogenicity.

The design provides for 45 adults & 45 children to be vaccinated at the preferred dose, whichever dose is chosen i.e., if the lower possible dose is preferred, additional participants will be vaccinated in groups AC3 or PC3 as required to reach a total of 45 at that dose.

5.2.2 Rationale for Comparator IRV Vaccine Route & Dose

IRV vaccine will be administered intradermally in 0.1mL aliquots.

1) At each of 4 anatomical sites for simulated PEP for all subjects (on ‘day SPEP+0’). This is a WHO-recommended PEP regime for previously immunised individuals, as discussed in section 5.2.3.
2) At each of 2 anatomical sites
   a) At baseline, on a single occasion, for groups AV1 and PV1. Although this is not a licensed or WHO recommended regimen, single visit 2-site ID IRV has been suggested as a potentially effective future option for rabies mass pre-exposure vaccination [45]. Establishing non-inferiority of ChAdOx2 RabG immunogenicity versus this shortened/minimal IRV regimen will help inform the decision of whether to move the development program forward into a subsequent phase II/III trial. It will also provide the first data (to our knowledge) regarding the immunogenicity of this regime in an African population: this will be of value in itself as, although we believe ChAdOx2 RabG is quite likely to be preferable, others feel that single visit ID IRV should be considered for PrEP.
   b) At baseline, on each of two occasions (study days 0 and 7), for group PV2. This is a gold-standard WHO recommended PrEP regime. Our view is that it is in some ways a less relevant comparator than single-visit regimes to be given to AV1 and PV1 (as it is already regarded by most in the field as being too expensive for widespread use), but we expect that others will wish to see head-to-head comparison of the immunogenicity of ChAdOx2 RabG with this regime.

c) At the final study visit (on ‘day SPEP+14’) for all subjects. All volunteers will therefore have received IRV at ≥2 visits by the end of the study, constituting robust pre-exposure prophylaxis (and hence providing them with benefit in the event of any future rabies exposure), independent of the vaccination given at enrolment. The current WHO-preferred PrEP regime is 2-site ID at days 0 and 7 but the lower dose / number of sites and shorter interval than our participants will receive are motivated by cost and practicality rather than efficacy, and use of the 7 day interval would impair our ability to monitor responses to the first SPEP dose (see section 5.2.3). A wide range of 2-visit regimes and intervals are known to provide effective PrEP, and the use of 4 × 0.1mL at baseline and a 14-day interval would, from immunological first principles, be expected to result in responses equivalent or superior to the WHO-recommended regime [8, 46-48].

At visits where intradermal IRV will be administered, this will be injected at 2-4 different anatomical sites. Acceptable sites for administration are left or right: deltoids, thighs and suprascapular areas as per WHO guidance [49].

5.2.3 Rationale for extended follow-up and simulated post-exposure prophylaxis.

Individuals who have received rabies PrEP and are subsequently exposed to the virus may be protected by any of the following three mechanisms:

- Pre-formed antibodies present at the time of exposure. VNA titers >0.5 IU/mL at the time of exposure are considered predictive of protection by this mechanism.
- An immunological memory response laid down by PrEP and recalled by PEP vaccination.
- Immunological memory responses laid down by PrEP vaccination and recalled by exposure to the virus (i.e., presumed subclinical infection), *without* PEP vaccination. There is evidence from animal studies that this can occur, even when VNA has waned to undetectable levels [50]. This is not well understood or robust in humans, and hence is *not* a preferred mechanism of protection, but it is likely to provide long-term partial protection to individuals who have had PrEP in the distant past but whose antibody titers have waned to <0.5 IU/mL.

In investigating a novel PrEP regime, it is therefore desirable to measure both the long-term maintenance of VNA titers (in the absence of antigenic restimulation) *and* the recall response to antigen (e.g. a simulated PEP vaccination).

During the extended period of follow-up in this study, participants will therefore have VNA titers measured before and after simulated PEP. Participants who opt in to the extended follow up of up to 5.5 years will have samples regularly taken in order to measure VNA levels, revealing the kinetic of neutralising antibody levels and allowing us to randomize those participants who have a VNA level below 0.5 IU/mL to receive SPEP at either their next visit or a visit towards the end of the study (up to 5.5 years post-enrolment).

We are not seeking to develop ChAdOx2 RabG for PEP use at this time, but instead to validate its use in a strategy of routine single visit ChAd PrEP for all, supplemented by single visit IRV PEP if needed following exposure.

Therefore for simulated PEP, participants will receive IRV according to a WHO recommended 4-site ID single-visit PEP schedule (see section 3.3.2). The scientific goal of this is to measure the memory immune response that was induced by ChAdOx2 RabG following re-exposure to the rabies glycoprotein: by administering IRV, we will effectively be measuring the secondary or recall response to ChAdOx2 RabG. The strength and kinetics of such recall responses may ultimately be a key factor in determining the efficacy of ChAdOx2 RabG in future clinical trials.

The initial IRV dose will be sufficient to allow us to measure the memory immune response to rabies glycoprotein. However, we also feel it is in the volunteers' best interests to have obtained an effective course of PrEP, independent of the efficacy or otherwise of the experimental ChAdOx2 RabG vaccine (as discussed in section 5.2.2). We will follow all participants who opt in to the extended follow up of up to 5.5 years until the end of the study, regardless of which study they received SPEP at, including taking blood for exploratory immunology.

5.2.4 *Rationale for age de-escalation approach*

The intended target population for ChAdOx2 RabG will be young children in rabies endemic regions. This study is conducted as an age de-escalation study starting first in adults before moving directly to the target population (children aged 2-6). Age de-escalation has been safely carried out directly from adults to children aged 2-6 in previous ChAd vectored vaccine studies conducted by the University of Oxford (see section 3.11).

As this is the first time ChAdOx2 RabG will be administered to young children, we will start with a lower dose of 1×10^{10} vp ChAdOx2 RabG as a further safety precaution before progressively escalating, dependent upon safety reviews, to the adult preferred dose, and using reactogenicity data at that dose level in up to 12 children to select the paediatric preferred dose (as detailed in section 5.3).

5.2.5 *Rationale for exclusion of participants who have received an adenovirus vectored vaccine within 6 months prior to enrolment, and advice to avoid other vaccines for 28 days after enrolment.*

At the time of writing of protocol v2.1, no adenovirus vectored vaccines were licensed for use in the study area, and it was considered reasonable to exclude any participant who had previously received any adenovirus vectored vaccine (which would have been through a previous research study).

It is now possible that participants, in particular adults, may have received ChAdOx1 nCoV-19 (Oxford / AstraZeneca / Serum Institute of India) or Ad26.COV2.S (Johnson & Johnson). There is no current experience of ChAdOx2 vaccination after either of these.

We are not aware of any grounds for safety concern about receipt of ChAdOx2 after a previous adenovirus. Reactogenicity of adenovirus vectors, including VITT, is not increased after administration of a second dose (although most evidence is based upon homologous boosting).

The possible concern about administration of ChAdOx2 RabG to individuals who have previously received an adenovirus vector is that anti-vector immunity induced by the first vaccine may attenuate immunogenicity of the ChAdOx2 RabG. We consider that this is unlikely to occur more than 6 months after administration of another vaccine because

- ChAdOx2 has not previously been used in Tanzania. The only adenovirus-vector serotypes which participants could plausibly have encountered are heterologous, which minimises attenuation of immunogenicity of the second dose.
- It has been demonstrated that re-administration of the same vaccine (e.g. second and even third doses of ChAdOx1 nCoV-19) can achieve immunogenicity, and that this tends to be strongest with a longer interval (e.g. greater than 8 weeks) since the first dose.

We have selected a 6-month minimum interval to further minimise the risk of a confounding effect of previous adenovirus vaccination, while also leaving open the possibility of gathering evidence which may support future use of ChAdOx2 RabG in populations who have received adenovirus-vectored vaccines, including against COVID-19, in the relatively distant past. History of previous adenovirus-vectored vaccination and baseline anti-vector antibody response data will be collected for exploratory analysis to assess for any relationship with ChAdOx2 RabG immunogenicity.

It is possible also that volunteers may be offered COVID-19 vaccines after enrolment in the study, and that these may include adenovirus-vectored vaccines. We will advise participants at enrolment to avoid receiving any other vaccine (including COVID-19 vaccines) for 28 days after enrolment. This is necessary to mitigate the risk that another vaccine could cause AEs or an effect upon the IMP's immunogenicity which might affect the study objectives. Beyond 28 days we believe that the risk of this is minimal, and that the risk that ChAdOx2 RabG could attenuate the immunogenicity of the subsequent vaccine is also minimal (for the same reasons listed above). We do not think that the very cautious 6-month interval between *previous* adenovirus-vectored vaccination and receipt of the IMP is appropriate *after* receipt of the IMP – we believe it would be in volunteers' interests to avoid further delay to COVID-19 vaccination in particular.

5.3 Safety oversight

5.3.1 UK Safety Data Reviews by National Ethical and Regulatory Authorities

Before this trial may commence, the SMC must review the safety report from RAB001 which will contain safety data (solicited AEs, unsolicited AEs, SAEs and laboratory AEs) up until day 28 post ChAdOx2 RabG vaccination from 12 UK adults. If the safety outcomes are favourable, the independent Safety Monitoring Committee (SMC) will recommend the

trial starts. Their recommendation and safety data will then be submitted to IRB, NatHREC and TMDA for review. When all three bodies concur with the SMC recommendations and approve the study to continue, vaccination of the first group of Tanzanian adults can commence.

As of August 2021, this review has taken place and all members of the SMC recommend commencing the RAB002 study. Review by the IRB, NatHREC and TMDA has also taken place, and vaccination of Tanzanian adults is approved.

5.3.2 Scheduled SMC and CI/PI data reviews during age de-escalation and dose escalation

Multiple reviews of safety / reactogenicity data are scheduled, completion of which will be required before each dose escalation or age de-escalation step, as shown in Table 5.

In general, reviews for **safety** will be performed by the SMC. Reviews for **tolerability** (i.e. judgment of the level of grade 1-2 AEs which are likely to be acceptable for ongoing development and deployment, but which do not constitute a significant safety risk to the participants) will be performed by the CI and PI.

The SMC will be convened by the study Sponsor, with composition and processes in accordance with section 9.5 and a safety monitoring charter. For each SMC review, the SMC will be provided with a formal safety report, and they will provide recommendation with regard to proceeding with vaccination.

Table 5 gives target enrolment figures at each stage and durations of follow-up data to be available prior to continuation to the following stage. Loss to follow-up is expected to be low, but some attrition may be unavoidable. Volunteers lost to follow-up between vaccination and study day 7 (i.e., before completion of the core safety data collection period) will be replaced with the next available volunteer. Each SMC or CI/PI review may proceed once ≥70% of the number of ChAdOx2 RabG recipients targeted for each stage have been followed up for the desired duration.

Table 5: Sequence of SMC and CI/PI data reviews and participant enrolment:

Study stage	Review required before commencing vaccination	Group(s) to be enrolled,<br>Total n to be enrolled & randomisation ratio
1<br>(adult)	Review 1, SMC/ IRB / NatHREC / TMDA:<br>Review of RAB001 UK data up to day 28.<br>Is reactogenicity acceptable to commence vaccination in RAB002?<br>This review has taken place.	AC1, n=3, ChAdOx2 RabG $2.5x10^{10}$ VP<br><br>Total n=3<br>No randomisation
2<br>(adult)	Review 2, CI/PI, tolerability:<br>Review of stage 1 data to day 3.<br>Is tolerability of $2.5x10^{10}$ VP acceptable for escalation to $5x10^{10}$ VP?<br>This review had taken place.	AC2, n=3, ChAdOx2 RabG $5x10^{10}$ VP<br>AV1, n=1, Verorab 2-site ID single-visit<br><br>Total n=4<br>Randomised 3 AC2: 1 AV1
3<br>(adult)	Review 3, CI/PI, tolerability:<br>Review of stage 2 data to day 3.<br>Is tolerability acceptable to vaccinate a further 9 participants with $5x10^{10}$ VP?<br>This review had taken place.	AC2, n=9, ChAdOx2 RabG $5x10^{10}$ VP<br>AV1, n=3, Verorab 2-site ID single-visit<br><br>Total n=12<br>Randomised 3 AC2: 1 AV1
4<br>(adult)	Review 4, CI/PI, tolerability:<br>Review of stage 1-3 data to day 7.<br>Based upon tolerability, should the adult preferred dose be $2.5x10^{10}$ or $5x10^{10}$ VP?<br>This review has taken place.	AC3, n=33 or 42, ChAdOx2 RabG adult preferred dose<br>AV1, n=11, Verorab 2-site ID single-visit<br><br>If adult preferred dose = $5x10^{10}$ VP → n=33 in group AC3 → total n=44, randomised 3:1<br><br>If adult preferred dose = $2.5x10^{10}$ VP → n=42 in group AC3 → total n=53, randomised 42:11
5<br>(paed)	Review 5, SMC, safety:<br>Review of stage 1-4 data to day 28<br>Is adult safety data satisfactory to commence vaccination of children?<br>This review has taken place.	PC1a, n=3, ChAdOx2 RabG $1x10^{10}$ VP<br><br>Total n=3<br>No randomisation
6<br>(paed)	Review 6, CI/PI, tolerability:<br>Review of stage 5 data to day 3.<br>Is tolerability of $1x10^{10}$ VP acceptable for escalation to half adult preferred dose?<br>This review had taken place.	PC1b, n=3, ChAdOx2 RabG half adult preferred dose<br><br>Total n=3<br>No randomisation
7<br>(paed)	Review 7, SMC, safety:<br>Review of stage 5-6 data to day 7.<br>Is safety in groups PC1a/b acceptable for escalation to full adult preferred dose?<br>This review has taken place.	PC2, n=3, ChAdOx2 RabG full adult preferred dose<br>PV1, n=2, Verorab 2-site ID single-visit<br>PV2, n=2, Verorab 2-site ID 2-visit<br><br>Total n=7<br>Randomised 3 PC2: 2 PV1: 2 PV2
8<br>(paed)	Review 8, CI/PI, tolerability:<br>Review of stage 7 data to day 3.<br>Is tolerability acceptable to vaccinate a further 9 participants with full adult preferred dose?<br>This review has taken place	PC2, n=9, ChAdOx2 RabG full adult preferred dose<br>PV1, n=6, Verorab 2-site ID single-visit<br>PV2, n=6, Verorab 2-site ID 2-visit<br><br>Total n=21<br>Randomised 3 PC2: 2 PV1: 2 PV2
9<br>(paed)	Review 9, CI/PI, tolerability:<br>Review of stage 5-8 data to day 7.<br>Based upon tolerability, should the paediatric preferred dose be half or full adult preferred dose?<br>This review has taken place	PC3, n=33 or 42, ChAdOx2 RabG paediatric preferred dose<br>PV1, n=22, Verorab 2-site ID single-visit<br>PV2, n=22, Verorab 2-site ID 2-visit<br><br>If paediatric preferred dose = full adult preferred dose → n=33 in group AC3 → total n=77,<br>randomised 3 PC3: 2 PV1 : 2 PV2<br><br>If paediatric preferred dose = half adult preferred dose → n=42 in group AC3 → total n=86,<br>randomised 42 PC3: 22 PV1 : 22 PV2

5.3.3 Dose reduction in the event of increased reactogenicity

At any point, if either the SMC, the CI or the PI find that a given dose level is poorly tolerated by volunteers, subsequent volunteers will instead be administered a lower dose, which will typically be half of that otherwise planned.

5.3.4 Holding Rules

If any of the criteria specified in Table 6 are met all vaccinations will be put on temporary hold until guidance from the SMC is obtained to proceed with vaccinations. The SMC will be convened by the study Sponsor and will be provided with a formal safety report to review, and the committee will provide a recommendation with regard to proceeding with further vaccinations.

SMC recommendation alone will be sufficient to resume vaccinations following the triggering of holding rules except in the circumstance of an SAE deemed possibly, probably or definitely related to ChAdOx2 RabG administration. In this circumstance, vaccinations may only restart following additional reviews of the event by the following: IHI IRB, Oxford Tropical Research Ethics Committee (OxTREC), National Health Research Ethics Sub-Committee (NatHREC) and TMDA (in addition to SMC review).

Table 6: Holding rule criteria.

**Severity or seriousness**	**Solicited / unsolicited**	**Local / systemic**	**Period of onset after vaccination (within, h)**	**Duration of persistence at grade 3 after onset**	**Number of participants experiencing same event**
Grade 3	Solicited	Local	48	72	≥2 *and* >20% of currently enrolled within age group*
Grade 3	Solicited	Systemic	48	48	≥2 *and* >20% of currently enrolled within age group*
Grade 3	Unsolicited, at least possibly related to ChAdOx2 RabG.	Local or systemic	48	48	≥2 *and* >20% of currently enrolled within age group*
Serious	At least possibly related to ChAdOx2 RabG	Any	Any	Any	Any 1

*For avoidance of ambiguity regarding the meaning of ‘≥2 *and* >20%’, exactly 2 participants within an age group (i.e., adult or paediatric) experiencing the same qualifying AE would trigger a holding rule if ≤9 participants in that age group have been vaccinated. If ≥10 participants in that age group have been vaccinated, the holding rule would trigger if >20% of participants experience the same qualifying AE.

5.3.5 Sentinel Participants

Protocol v2.1 specified criteria for SMC review to be triggered by vaccination of sentinel participants within the low and higher-dose paediatric groups.

Equivalent safeguards are now provided by the combination of the holding rules and the extended series of step-by-step reviews during progression from study stages 5 to 8 in the revised design (tabulated in Table 5).

5.4 Trial Centre

The study will take place at the IHI Clinical Trial Facility (CTF) located in Bagamoyo town. The facility has clinical consultation rooms, a pharmacy, vaccination room, data management room and an observation area for study participants. There is a resuscitation room that is equipped with oxygen, suction, defibrillator and resuscitation kits. The facility is supported by Bagamoyo Research and Training Centre (BRTC) laboratory located within the grounds of Bagamoyo District Hospital (BDH). BRTC laboratory provides routine haematology, biochemistry and microbiology services. The laboratory can also conduct a wide range of immunology and biomedical analyses.

The clinical trial facility has experienced trained staff including pharmacists, physicians and highly experienced nurses. The facility has a Quality Assurance department responsible for ensuring trials are conducted according to relevant Protocols and SOPs and data collected is of high quality and integrity. Study physicians and nurses are ACLS and PALS certified and are involved in regular re-training activities involving simulations.

Although the study will be run on an outpatient basis and it is not anticipated that unscheduled visits will be required, a clinician will be accessible 24 hours a day by phone to provide medical care to participants as needed outside of the scheduled study visits. All participants who require outpatient treatment will be encouraged to come to the CTF during the day. All cases that require inpatient care will be seen at BDH. BDH is a public hospital providing secondary level health care services. The District Hospital has one paediatric ward and a general ward. There are facilities for basic radiology and ultrasound. Routine paediatric surgery is carried out at the District Hospital. Referral to tertiary level facilities is rarely required, but, in cases where such referrals are required participants will be sent to Muhimbili National Hospital in Dar es Salaam. All SAEs requiring treatment will be managed in the inpatient wards in BDH.

6 PARTICIPANT IDENTIFICATION AND RECRUITMENT

6.1 Study Population

Participants will be recruited from around Bagamoyo town in Bagamoyo district. Bagamoyo district is one of the 6 districts of the Pwani region of Tanzania (area 8,462.63 km^2). According to the 2012 Tanzania National Census, the population of the Bagamoyo District was 343,412. The population of Bagamoyo is highly mixed due to migration and settlement of different ethnic groups.

6.2 HIV Prevalence

Potential participants will be tested for HIV and HIV positive individuals excluded from the study. The prevalence of HIV in Tanzania is estimated to be about 5.9% [51]. The prevalence among healthy children in the community is thought to be below 1%. A Prevention of Mother to Child Transmission of HIV program has been operational in Bagamoyo since 2010 and the Ministry of Health anti-viral program is operational. Highly Active Antiretroviral Therapy (HAART) is readily available to all HIV positive people at selected government hospitals.

6.3 Sensitization

After approval from ethics committees, local authorities will be informed about the study in order to receive permission to approach the community. A series of meetings will then be held to explain the study to the potential participants in the community. These meetings will consist of sensitization meetings for general information and pre-screening meetings for more detailed information. We will use community healthcare workers or trained study team members to inform potential participants and parents/guardians to the sensitization meetings. Volunteers that previously participated in an epidemiological study conducted at IHI clinical trial facility using similar sensitization procedures and have consented to be contacted for possible participation in a study like this as per the initial informed consent, may also be invited to the sensitization meetings. The need and the difficulties of developing a vaccine against rabies will be discussed in a culturally appropriate fashion, as well as an outline of the proposed trial, including the rationale, the background data available, the study objectives and the screening and consent procedures. Particular attention will be paid to study procedures, immunization and blood collection. To avoid misunderstandings, the purpose of blood collection and the associated risks will be explained in a culturally sensitive fashion. Opportunity will be provided for the community members to ask questions and get responses from the Investigators.

Potentially interested participants including the parents/guardians will be asked to register their names at the end of the meetings with the study staff. Potential participants will be invited to attend a pre-screening meeting at CTF.

At the pre-screening meeting the PI and trained study clinicians and nurses will provide detailed information about the study using the PIS in a group and individual sessions. A copy of the PIS will also be provided before commencing these meetings. The PI will ensure potential participants or parents/guardians fully understand the risks and benefits associated with participation in the study.

It will be stressed that ChAdOx2 RabG is an experimental vaccine and cannot be guaranteed to provide protection against rabies virus and that therefore it will still be necessary to seek full post-exposure vaccination and treatment in the event of an exposure to rabies. It will also be made clear that the study will require HIV testing of the participants. It will be made clear that screening will include testing for a range of diseases (not just HIV) and non-disease criteria, so exclusion from participating in the trial does not imply being HIV positive and the confidentiality of those participants infected with HIV will be protected. It will be stressed that this is the beginning of a long process of ChAdOx2 RabG vaccine development.

Opportunity will be provided for the potential participants to ask questions and get responses from the PI and study physicians. Those who are still interested in taking part will then be invited for a formal screening visit where an informed consent form will be signed or thumb-printed. A copy of the PIS will be given to the potential participants to take home and read further including with others such as spouses, friends or relatives.

6.4 Inclusion Criteria

Only participants who meet all the inclusion criteria will be enrolled into the trial;

- Adult groups: Healthy male or female adults aged 18-45 years at the time of enrolment with signed consent.
- Adult groups (Female only participants): Must be non-pregnant (as demonstrated by a negative urine pregnancy test) and willing to use an effective form of contraception.

  *Female volunteers are required to use an effective form of contraception during the course of the study, or if they agree to the extended period of follow-up of up to 5.5 years, use an effective form of contraception during the first year of enrolment in the study. As this is a Phase I, study and there is currently no information about the effect of this vaccine on a foetus. Acceptable forms of contraception for female volunteers include:

  - Established use of oral, injected or implanted hormonal methods of contraception.
  - Placement of an intrauterine device (IUD) or intrauterine system (IUS).
  - Total abdominal hysterectomy.
- Paediatric Groups: Healthy male or female young children aged 2-6 years at the time of enrolment with signed consent obtained from parents or guardians.

- Paediatric Groups: completion of the Expanded Programme on Immunisation (EPI) at least 6 months prior to study enrolment.
- Planned long-term (at least 61 months from the date of the first vaccination) or permanent residence in Bagamoyo town.
- Adults with a Body Mass Index (BMI) 18 to 30 Kg/m^2; or young children with Z-score of weight-for-age within ±2SD.
- Correctly answer all 10 questions on the protocol and study procedures understanding questionnaire within 2 attempts.

6.5 Exclusion Criteria

The participant may not enter the trial if ANY of the following apply:

- Clinically significant congenital abnormalities as judged by the PI or other delegated individual.
- Clinically significant history of skin disorder, allergy, cardiovascular disease, respiratory disease, endocrine disorder, liver disease, renal disease, gastrointestinal disease and neurological illness which may significantly increase the risk to the volunteer because of participation in the study, affect the ability of the volunteer to participate in the study or impair interpretation of the study data as judged by the PI or other delegated individual.
- Any confirmed or suspected immunosuppressive or immunodeficient state, including HIV infection; asplenia; recurrent, severe infections and chronic (more than 14 days) immunosuppressant medication within the past 6 months (inhaled and topical steroids are allowed).
- Any condition which would place the individual at elevated risk of serious COVID-19 infection, or any other factor which may make the individual eligible for priority COVID-19 vaccination (i.e. ahead of others in their age group).
- History of cancer (except basal cell carcinoma of the skin and cervical carcinoma in situ).
- History of allergic disease or reactions likely to be exacerbated by any component of the vaccines, including IRVs e.g. amphotericin B, chlortetracycline, neomycin, polymyxin, streptomycin
- Any history of anaphylaxis in relation to vaccination.
- Clinically significant laboratory abnormality as judged by the PI or other delegated individual.
- Receipt of any previous rabies vaccinations, including an incomplete course.
- History of vaccination with previous adenoviral vectored vaccines in the 6 months prior to enrolment in the study (see section 5.2.5), or of vaccination with any other vaccine (including non-adenovirus-vectored COVID-19 vaccines) in the 28 days prior to enrolment.
- Planned / likely receipt of any other vaccine within 28 days after enrolment.

- History of bleeding disorder (e.g., factor deficiency, coagulopathy or platelet disorder), or prior history of significant bleeding or bruising following IM injections or venepuncture, or continuous anticoagulation e.g., with warfarin
- History of confirmed major thrombotic event, (including cerebral venous sinus thrombosis, deep vein thrombosis, pulmonary embolism) or,
- History of antiphospholipid syndrome.
- History of prior receipt of unfractionated heparin
- History of heparin induced thrombocytopenia
- Receipt of any blood products/ immunoglobulins within the three months preceding the planned administration of the vaccine candidate.
- Participation in another research study involving receipt of an investigational product in the 30 days preceding enrolment, or planned use during the study period.
- Seropositive for hepatitis B surface antigen (HBsAg) or hepatitis C (HCV IgG).
- Likelihood of travel away from the study area.
- Female participant who is pregnant, lactating or planning pregnancy during the course of the trial.
- Scheduled elective surgery or other procedures requiring general anaesthesia during the trial.
- Any other significant disease, disorder or situation which, in the opinion of the Investigator, may either put the participants at risk because of participation in the trial, or may influence the result of the trial, or the participant's ability to participate in the trial.
- Contraindication to use of paracetamol

6.6 Contraindications to subsequent vaccination

6.6.1 <u>Indications for deferral of vaccination</u>

These events constitute contraindications to administration of the IMP at that point in time and will result in deferral of vaccination to another time within the protocol specified window, or withdrawal at the discretion of the Investigator. AEs should be followed-up according to the instructions in Section 9.

- Acute disease at the time of administration of the IMP (acute disease is defined as the presence of a moderate or severe illness with or without fever). All vaccines can be administered to persons with a minor illness such as diarrhoea or mild upper respiratory infection without fever, i.e., axillary temperature < 37.5°C.
- Axillary temperature of ≥ 37.5°C.
- Abnormal laboratory parameters that have been determined to be clinically significant.

- Pregnancy and breast feeding during the extended follow-up period of 1-5 years. Any participant who is pregnant or breast feeding will have the SPEP+0 visit (which initiates receipt of Verorab, see section 7.5) delayed until pregnancy and breast feeding is finished.
- On request of the participant.
- On advice of the safety monitor.
- On advice of the Investigators.

6.6.2 *Permanent Contraindications for further vaccination*

The following AEs constitute absolute contraindications to further administration of the IMP. If any of these AEs occur during the study, the participant must not receive additional doses of vaccine, but will continue with the full safety monitoring procedure (if the participant has already received a vaccination) as per protocol:

- Acute allergic reaction, significant IgE-mediated event, angioedema or anaphylactic shock following the administration of vaccine investigational product.
- Any confirmed or suspected immunosuppressive or immunodeficient condition, including human immunodeficiency virus (HIV) infection.
- The participant experiences an SAE that is determined to be related to the study product.
- The participant starts taking a concomitant medication for a chronic illness.
- Pregnancy during the first year of the study (for participants who consent to undergo extended follow up of up to 5.5 years) or at any time during the study (for participants who do not consent to undergo extended follow up).
- On request of the participant.
- On advice of the Investigators.
- On advice of the IRB, regulatory authority or SMC.

6.6.3 *Withdrawal of study participant*

Participants have a right to withdraw from the study at any time and are not obliged to give their reasons for doing so. The Investigator may withdraw the participant at any time in the interests of the participant’s health and well-being. In addition, the participant may withdraw/be withdrawn for any of the following reasons:

- Any serious adverse event (SAE) deemed to be related to the IMP.
- Any adverse event (AE) that, according to the clinical judgment of the Investigator, is considered a contraindication to proceeding with the study procedures.
- The use of concomitant, chronic medication active on the immune system (systemically administered steroids, immunosuppressive agents) or potentially affecting ChAdOx2 RabG.
- Participant completely lost to follow-up.
- Poor compliance with study procedures, such that participant safety or completeness and integrity of data are jeopardized.
- Administrative decision by the Investigator.

- Ineligibility (either arising during the study or retrospectively, having been overlooked at screening).
- Significant protocol deviation.

If a participant withdraws from the study, blood samples collected before withdrawal will be used/ stored to address the safety and immunogenicity objectives of the current protocol unless the participant specifically requests otherwise. All participants who are withdrawn after having received vaccination will be followed up until the end of the study for safety reasons (if they consent to this) but will not receive additional vaccinations or have further study samples or data collected. All participants can withdraw their permission to store the samples for future studies at any time.

6.6.4 Replacement of Individual Participants after Withdrawal

If for any reason a participant cannot receive the first vaccination, they can be replaced by a backup participant that has been screened and found to be eligible. Randomisation would not have occurred and so can proceed unaffected.

In the unlikely event that a participant is lost to follow-up or withdraws between randomisation and 7 days after receiving ChAdOx2 RabG, this participant will be replaced with the next available eligible participant (without randomisation).

However, if a participant is withdrawn more than 7 days after receiving ChAdOx2 RabG, or at any point after receiving IRV, there will be no replacement.

6.7 Pregnancy

Should a participant become pregnant during the trial (for participants who do not consent to undergo extended follow up of up to 5.5 years), or during the first year of the trial (for participants who do consent to undergo extended follow up of up), she will not be vaccinated but will be followed up as other participants and in addition will be followed until pregnancy outcome, with the participant’s permission. We will not routinely perform venepuncture on such participants.

The reason for withdrawal will be recorded in the CRF. If the participant is withdrawn due to an adverse event, the Investigator will arrange for follow-up visits or telephone calls until the adverse event has resolved or stabilised.

Should a participant become pregnant during the extended follow up period, they will not undergo the SPEP+0 visit, which initiates receipt of Verorab, until pregnancy and breast feeding are completed. They will remain in the study until pregnancy and breast feeding are complete. We will not routinely perform venepuncture on such participants while pregnant or breast feeding.

6.8 Managing volunteers during COVID-19 outbreak

Reports from authorities indicates that hospitalized COVID-19 cases have declined significantly. However, the population is still being urged to take all necessary precautions as the transmission in still ongoing.

The suspect SARS-COV2 case will be defined as:

- A patient with acute respiratory illness (fever and at least one sign/symptom of respiratory disease, e.g. cough, shortness of breath, sore throat, rhinitis, OR
- A patient with any acute respiratory illness AND having been in contact with a confirmed or probable COVID-19 case in the last 14 days prior to symptom onset; OR
- A patient with severe acute respiratory illness (fever and at least one sign/symptom of respiratory disease, e.g., cough, shortness of breath; AND requiring hospitalization) AND in the absence of an alternative diagnosis that fully explains the clinical presentation.

<u>Vaccine associated fever</u>

- Both VERORAB and adenovirus vectored vaccines are known to cause self-limiting vaccine related reactions (fever, malaise, etc) particularly during the first 48 hours following vaccination.
- Fever that arises and resolves within 48 hours of vaccination can be considered to be vaccine related if it is **not** accompanied by any respiratory symptoms. In this circumstance, it will not be considered a suspect SARS-COV2 case and will not routinely require referral for covid19 testing.

All participants who present with symptoms suggestive of SARS-COV2 at screening, during vaccinations and follow-up visits will be managed as shown by guidelines in *Table 7*. All volunteers who require COVID-19 testing will be referred to public testing facilities where samples will be collected for testing at National Public Health Laboratory. The study will cover the costs of treatment of volunteers who are diagnosed with COVID-19. The management of COVID-19 will follow the guidelines developed by the Ministry of Health, Community Development, Gender, Elderly and Children (MoHCDEC).

Table 7: Guidelines on management of COVID-19 cases

**Situation**	**Action**
Asymptomatic infection at any time	No need for testing. Risk to staff can be mitigated by PPE.
Symptomatic at D0 and at subsequent vaccinations:	Advice and facilitate COVID-19 testing at Public testing facility.<br>If meets case definition for suspect COVID-19:<br>• Advise and facilitate COVID-19 testing at Public testing facility<br>• Postpone vaccination until COVID-19 testing result is available<br>If positive for COVID-19<br>• Postpone vaccination until:<br>o At least 14 days from the onset of symptoms **AND**<br>o Symptoms have resolved and the participant has been afebrile for at least 24 hours prior to vaccination<br>If negative for COVID-19<br>• Postpone vaccination if symptoms are moderate or severe or if febrile.<br>• Vaccinate if symptoms resolve or become mild and afebrile. (Volunteer need to be afebrile for 24 hrs before vaccination).
Symptomatic at follow-up visits	If meets case definition for suspect COVID-19:

	• Advise and facilitate COVID-19 testing at Public testing facility<br>• Follow-up the patient by phone and discourage visit at study clinic while awaiting test results.<br>If positive for COVID-19<br>• Advise patient to isolate as per the current local guidelines and prescribe supportive treatment. Consider arranging admission if indicated for unwell patients.<br>• Postpone sample collection until better<br>• Continue follow up with home visit by study staff with PPE.<br>If negative for COVID-19<br>• Continue with follow-up as planned

Understanding of appropriate investigation and management of COVID-19 continues to evolve, as do local guidance and facilities. The trial's guidance on management of possible and confirmed COVID-19 may be altered from that outlined in Table 7 to an alternative policy which the CI and PI agree offers at least equivalent quality of care and safety to participants, staff and the community.

The protocol seeks to address the possibility that volunteers may have recently received COVID-19 vaccination or may be offered it during the study. The investigators have considered the following possibilities:

- Interference of COVID-19 vaccination with evaluation of the IMP, impairing ability to reach this study's objectives.
- Interference of study vaccination with efficacy of COVID-19 vaccination
- Illness occurring due to delay to COVID-19 vaccination due to participation in the study.

These possibilities are addressed by exclusion criteria and protocol instructions defining permissible intervals between prior COVID-19 vaccination and study vaccination (see sections 5.2.5, 6.5, and 7.5). In brief, the study will take a cautious approach to excluding volunteers who have received potentially interfering vaccines *before* enrolment and will exclude participants at high risk of COVID-19 (for whom any delay to COVID-19 vaccination is undesirable) or who are due to receive a non-study vaccine in the 28 days after enrolment. We do not believe that receipt of ChAdOx2 RabG is likely to affect efficacy of COVID-19 vaccines (including those based upon heterologous adenovirus serotypes) given at least 28 days after ChAdOx2 RabG.

7 TRIAL PROCEDURES

7.1 Informed Consent

Adult participants must provide adequate informed consent and personally sign or thumb print and date the latest approved version of the Informed Consent form before any trial specific procedures are performed. The parent/guardian of child volunteers must provide adequate informed consent and sign or thumb print and date the latest approved version of the informed consent form before any trial specific procedures are performed.

Written versions of the Participant Information Sheet (PIS) and Informed Consent Form (ICF) will be presented to the participants detailing no less than: the exact nature of the trial; what it will involve for the participant; the implications and constraints of the protocol; the known side effects and any risks involved in taking part. It will be clearly stated that the participant is free to withdraw from the trial at any time for any reason without prejudice to future care, without affecting their legal rights and with no obligation to give the reason for withdrawal.

Prior to screening, adult participants and parents/guardians of child participants will be required to demonstrate their understanding of the study by answering a questionnaire, provided by study personnel, which will consist of 10 true/false statements. Participants / parents / guardians must respond correctly to all 10 questions. They will have a maximum of two attempts after which those who fail to correctly answer all questions will be excluded. Those who correctly respond to all 10 questions will be allowed to sign the ICF.

Reconsenting of participants already involved in the study due to any changes will also involve a questionnaire. However, failure to respond correctly to all 10 questions in two attempts will not result in exclusion, as the participants will already be enrolled in the study. Instead participants/parents/guardians will be able to review the PIS and ICF again and discuss the study with the study team before attempting the questionnaire again.

The participant will be allowed as much time as wished to consider the information, and the opportunity to question the Investigator, or other independent parties, to decide whether they will participate in the trial. Written Informed Consent will then be obtained by means of participant-dated signature or thumb-print and dated signature of the person who presented and obtained the Informed Consent. If the participant is illiterate a literate witness will be present during the consent process and verify the process by signing and dating the ICF. Consenting will be done by trained study staff including the clinicians and nurses. A copy of the signed ICF will be given to the participant. The original signed form will be retained at the trial site.

7.2 Screening and Eligibility Assessment

Screening procedures will only start once a participant or parent/guardian has provided a written informed consent and continue until all participants have been enrolled and at least

2 eligible back-up participants have been identified. Two screening visits will be conducted for each participant.

Participants will not be considered to be enrolled until treatment allocation during the day 0 visit (i.e., randomisation, or vaccination for non-randomised groups).

7.2.1 Screening Visit 1 (SC1)

Only participants who meet eligibility criteria and have signed/thumb-printed the informed consent will undergo a full screening procedure. At screening visit 1, study staff will go through the PIS for the second time and describe the study in detail. This will be done in a group session where potential participants will be encouraged to ask questions. After the group session each participant will have a private session with study staff where additional clarification will be provided and time for more questions provided.

Participants will be assigned with a study ID on a first come first serve basis. A photo will also be taken to allow preparation of Study ID cards which will be given after the first vaccination. In the interim all participants will receive temporary study ID forms.

The following procedures will then take place:

- Taking the medical history of a participant, including history of adenovirus-vectored vaccination (e.g. COVID-19 vaccines).
- Complete physical examination including vital signs, height, weight and blood pressure.
- Venous blood sample collection for the following baseline and screening analyses:
    - Haematology: Full Blood Count (CBC) which includes red blood cells, platelets, white blood cell count, lymphocyte count, haemoglobin, mean corpuscular volume and mean corpuscular haemoglobin concentration.
    - Biochemistry: serum creatinine and ALT.
    - Serology: HIV, HCV and HBV tests.
    - Thick blood smear
- Collection of urine for the following analyses:
    - Dipstick for blood, glucose and protein.
    - Pregnancy test (for females in adult groups only).
- Checking of inclusion and exclusion criteria

Once all specific visit procedures are completed, participants will be allowed to go home and asked to return for Screening Visit 2.

The screening laboratory results will be reviewed by study clinicians before participants are seen at screening visit 2. These procedures will be documented in the case report forms (CRFs) and relevant clinical notes, which will be kept in the individual study participant's file. After collection of all screening information, the study team will review and assess the eligibility of each participant. All participants will be contacted and informed of the specific date to come to CTF for Screening Visit 2.

7.2.2 Screening Visit 2 (SC2)

During Screening Visit 2, participants will be informed of their screening results and receive HIV and hepatitis post-test counselling. Study clinicians will inform them if they are eligible for enrolment into the study. The principal investigator (PI) will have the final decision on the eligibility of a participant. Only those laboratory results with clinical relevance will be provided to the participant. Participants excluded from this trial because of clinically significant medical conditions may be managed initially by study clinicians and/or referred to the routine health care system for further evaluation and treatment as necessary. In the event that a participant tests positive for HIV, HBV, or HCV, he or she will be referred to the appropriate public health facility for further counselling and treatment.

Recruitment will continue until all the required number of participants per Group has fulfilled all inclusion criteria and none of the exclusion criteria. Should the participant or the parents of participants change their mind and decline to participate prior to immunization, additional participants will be screened until the required number of participants per group is enrolled. A list of eligible participants per group will be generated and used to identify the participants eligible for immunization. Screening tests will be completed within 30 days prior to immunization.

7.3 Baseline Assessments

Before vaccination all participants with undergo baseline assessment. This will include collection of safety and immunology samples. Once all final eligibility criteria are reviewed including safety laboratory results, eligible participants will be vaccinated. The window period as shown in APPENDIX A: SCHEDULE OF PROCEDURES will be used in case a participant cannot be available on a scheduled date. Pre-vaccination (Day 0) laboratory assessments will be done within 24 hours prior to vaccination. However only clinical assessments and vital signs collected on the day of vaccination will be recorded in the pre-vaccination CRF.

7.4 Allocation / randomisation procedure

Allocation to groups receiving different baseline study vaccination

Allocation to groups will proceed as set out in Table 5, and will take place at enrolment i.e., immediately prior to vaccination.

For non-randomised groups the next eligible participants will be assigned to these groups by study staff.

For randomised groups, generation of a master randomization list will be done by an independent statistician from IHI. A block size of four to seven will be used, at the statistician's discretion and as appropriate for the randomisation being performed at each study stage. Randomisation ratios will vary at different study stages as set out in Table 5.

The randomization list will contain treatment allocation (vaccine type and/or dose) which will linked to a study ID, as described below.

Study ID will be assigned to participants in the order in which they are screened for the trial.

Allocation will not be concealed from staff or participants- this is an open-label trial.

Participants will receive Study ID cards with Subject Numbers at the screening visit 1. At first vaccination visit, after verification of eligibility criteria, subjects will be allocated a treatment assignment in the order they present for vaccination using a master randomization list generated by the independent statistician. The correspondence between the Subject ID and the treatment allocation will be noted down in the CRF and subsequently entered in the OpenClinica Electronic Data Capture (EDC) system.

Allocation to different timing of SPEP

Participants who opt in to extended follow up (introduced by protocol v5.0 under amendment SA003) and return a VNA level below 0.5 IU/mL will be randomised, upon availability of that VNA result, 1:1 to receive SPEP at the next available visit or at the latest opportunity before their final study visit (up to 5.5 years after enrolment). The earliest visit at which SPEP may take place will be the day 365 visit. Randomization will be stratified by age group and baseline vaccine regime (adult preferred dose of ChAdOx2, adult non-preferred dose of ChAdOx2, adult single-visit Verorab, paediatric preferred dose of ChAdOx2, paediatric non-preferred dose of ChAdOx2, paediatric single-visit Verorab, paediatric two-visit Verorab). Within each of these groups, further stratification will take place by inclusion in the “immune subset”, as outlined in the current version of the Laboratory Analysis Plan. Randomisation will use a block size of 2 or 4 at the discretion of the local statistician.

7.5 Detailed scheduled study visits

The study visits (excluding retention visits), and an outline of the purposes for which immunological samples will be used, are summarised in APPENDIX A: SCHEDULE OF PROCEDURES but detailed descriptions of each visit are listed below:

<u>Day 0: (Vaccination with ChAdOx2 RabG or inactivated rabies vaccine)</u>

Participants will first be identified using their Study ID cards and vital signs and body weight will be measured.

Blood sample will be collected for the following analyses:

- haematology (full blood count);
- biochemistry (including creatinine and ALT);
- immunology.

Medical history and focused physical examination (informed by complaints) will be conducted by study clinicians who will also check for inclusion/exclusion criteria, contraindications to vaccination and elimination criteria. Safety laboratory results will be reviewed by study clinician before vaccination and checked by the PI (or designee).

Participants will be allocated to group as described in section 7.4.

IM ChAdOx2 RabG or the 2 anatomical site ID PrEP IRV will then be administered by trained study staff according to the site vaccination SOP. Each participant will be assessed for at least 1 hour after vaccination to evaluate and treat any acute adverse events. During this time the following will be recorded:

- Post vaccination vital signs
- Post-vaccination solicited signs/symptoms.
- Post-vaccination unsolicited adverse events.
- Post-vaccination SAEs.
- Any concomitant medication.

At the investigators' discretion, recipients of ChAdOx2 RabG may be provided with paracetamol if they develop systemic reactogenicity.

Participants will be advised not to receive any other vaccine for 28 days from enrolment (see section 5.2.5).

Days 1, 2, 3, 4, 5 and 6

On day 1 participants will be seen at CTF where medical history, temperature monitoring and physical examination will be performed and recorded in the CRF. AEs will be recorded in the CRF. Blood samples may be collected for exploratory immunology, which may include haematological markers of innate immune responses. This visit at the CTF may acceptably be performed on day 2 if day 1 is not possible.

Each participant will be visited at home on the remaining days in this period by a trained study staff member for assessment and recording of any solicited and unsolicited AEs. If necessary, the participant will continue to be seen regularly until the AEs have resolved or stabilised. During the home visit, the following will be assessed and recorded in the CRF;

- Axillary body temperature.
- Local and general solicited adverse events.
- SAEs experienced by the participant since the last visit.
- Unsolicited adverse events experienced by the participant since the last visit.
- Concomitant medication taken.

Day 7 (-1/+3 days):

Participants will be seen at CTF where medical history, temperature monitoring and physical examination will be performed and recorded in the CRF. AEs will be recorded in the CRF.

Blood samples will be collected for the analysis of:

- haematology (full blood count);
- biochemistry (including creatinine and ALT);
- immunology.

Participants allocated to 2-visit baseline IRV vaccination (Group PV2) will receive 2 anatomical site ID vaccination.

Day 14 (-1/+3 days;)

Participants will be seen at CTF where medical history, temperature monitoring and physical examination will be performed and recorded in the CRF. AEs will be recorded in the CRF.

Blood sample will be collected for the analysis of:

- haematology (full blood count);
- biochemistry (including creatinine and ALT);
- immunology.

Day 28 (-7/+2 days)

Participants will be seen at CTF where medical history, temperature monitoring and physical examination will be performed and recorded in the CRF. AEs will be recorded in the CRF.

Blood sample will be collected for the analysis of:

- haematology (full blood count);
- biochemistry (including creatinine and ALT);
- immunology.

Day 56 (± 7days);

Participants will be seen at CTF where medical history, temperature monitoring and physical examination will be performed and recorded in the CRF. Any SAEs reported by the participant or their parents during the visit, and not previously recorded, will be recorded in the CRF.

Blood sample will be collected for the analysis of:

- haematology (full blood count);
- biochemistry (including creatinine and ALT);
- immunology.

Day 182 (± 14 days);

Participants will be seen at CTF where medical history, temperature monitoring and physical examination will be performed and recorded in the CRF. Any SAEs reported by the participant or their parents during the visit, and not previously recorded, will be recorded in the CRF.

Blood sample will be collected for the analysis of:

- haematology (full blood count);
- biochemistry (including creatinine and ALT);
- immunology.

Days 365, 730, 1095, 1460, 1835 (-92/+181 days); which may vary on an individual participant basis according to participant availability and at investigators' discretion.

For participants receiving simulated PEP (SPEP) at this visit, this will become the 'SPEP+0' visit and the procedures for the 'SPEP+0' visit followed instead.

For participants for whom this is NOT the 'SPEP+0' visit, they will be seen at CTF where medical history, temperature monitoring and physical examination will be performed and recorded in the CRF. Any SAEs reported by the participant or their parents during the visit, and not previously recorded, will be recorded in the CRF.

Blood sample will be collected for the analysis of:

- immunology.

Simulated PEP = day 'SPEP+0'

This will take place at the Day 365 visit for participants who do not opt in to the extended follow up. This may take place at any one of the Day 365, 730, 1095 or 1460 visits for participants who have a VNA result less than 0.5 IU/mL at an earlier visit. For all remaining participants, this will take place at study day 1825 (-92/+181 days) which may vary on an individual participant basis according to participant availability and at investigators' discretion

Participants will first be identified using their Study ID cards and vital signs and body weight will be measured. Medical history and focused physical examination (informed by complaints) will be conducted by study clinicians who will also check for contraindications

to vaccination and elimination criteria. Any SAEs reported by the participant or their parents during the visit, and not previously recorded, will be recorded in the CRF.

Collection of urine for the following analyses:

- Pregnancy test (for females in adult groups only). Any participant who is pregnant will not undergo the rest of the SPEP+0 visit, and will instead be invited to have the SPEP+0 visit once their pregnancy and any subsequent breast feeding is completed.

Blood sample will be collected for the analysis of:

- immunology.

4 site ID IRV will then be administered according by trained study staff according to the site vaccination SOP.

Day ‘SPEP+7’ (-1/+1 relative to ‘day SPEP+0’)

Participants will be seen at CTF where medical history, temperature monitoring and physical examination will be performed and recorded in the CRF. Any SAEs reported by the participant or their parents during the visit, and not previously recorded, will be recorded in the CRF.

Blood sample will be collected for the analysis of:

- immunology.

Day ‘SPEP+14’ (± 2-day relative to ‘day SPEP +0’)– Final Visit

Participants will first be identified using their Study ID cards and vital signs and body weight will be measured. Study clinicians will check for contraindications to vaccination and elimination criteria. Any SAEs reported by the participant or their parents during the visit, and not previously recorded, will be recorded in the CRF.

Collection of urine for the following analyses:

- Pregnancy test (for females in adult groups only). Any participant who is pregnant will not undergo the rest of the SPEP+14 visit, and will instead be invited to have the SPEP+14 visit once their pregnancy and any subsequent breast feeding is completed.

Blood sample will be collected for the pre-vaccination assessment of immunology.

2 site ID IRV will then be administered according by trained study staff according to the site vaccination SOP.

Retention visits

Due to the extended period of follow-up of up to 5.5 years and the one-year gap between some visits, participants/parents/guardians may be contacted between the home and clinic visits listed above. This may include phone calls, as well as home and site visits by participants/parents/guardians. No blood sampling will occur at retention visits.

7.6 Unscheduled Visits:

Participants will be advised to seek first line health care at CTF where they were enrolled during working hours and at BDH outside the working hours. The CTF and BDH will be staffed by clinically qualified health workers who are on call 24 hours a day, 7 days a week. If needed participants will be referred, and if urgently needed, transported to Aga Khan Hospital or Muhimbili National Hospital. In the event the unscheduled visit occurs in another health facility then whenever possible, clinical notes related to the management of that event will be retrieved.

Participants will also be informed that community health workers will be readily available to facilitate access to care during the course of the trial. CHW will have access to a study clinician for consultation by mobile phone 24 hours a day and 7 days a week. Reimbursements for transport to CTF or BDH will be provided when participants present for unscheduled visits.

7.7 Sample Handling

Samples collected during the study will be used to address the study objectives and obtain a better understanding of vaccine immune responses following immunization with ChAdOx2 RabG. Prior to their use, the participant may withdraw permission for future use of specimens at any time. If a participant withdraws his or her permission for future use of specimens, the Investigator or designee will destroy all known remaining specimens and report this destruction to the participant and the IRB. This decision will not affect the participant's participation in this protocol, or any protocols supported by Ifakara Health Institute.

All samples stored will be labelled with the participant's study identification (ID) number, which cannot identify the study participant directly but is linkable to other research databases (e.g., questionnaires, clinical assessments, logbooks) generated by the main study. The subject identification log linking the study participant ID number to the name of the participant will be maintained with access limited to authorized research team members. In the event of samples being requested in the future, only the PIs or study coordinator(s) will have access to the log linking the study participant to the samples.

At the completion of the clinical study protocol and the primary and secondary immunology studies described in this protocol, remaining samples will either be destroyed or stored indefinitely (Section 13.9).

In more detail: for samples held at IHI in Tanzania, samples from participants whose parents/guardians or themselves did not provide permission to store them will be discarded after the primary and secondary analyses described in this protocol have been completed. Samples will also be destroyed if the study participants withdraw their permission to store the samples for future studies during the trial. If there is no withdrawal all remaining samples in Tanzania will be stored for future use. Any additional immunological analyses on these samples will be limited to rabies vaccine development unless specific permission for additional studies is obtained from the relevant IRBs. Transfer to another protocol will require approval from the IRBs. In the future, other investigators (both at IHI and outside) may wish to study these samples and/or data. In that case, IRB approval must be sought prior to any sharing of samples. Any clinical information shared about the sample with or without participant identifiers would similarly require prior IRB approval.

The samples exported to UK will be stored at the Jenner Institute, University of Oxford and used for analyses as outlined in the protocol during the study period. However, once the ethical approval on the protocol expires (1 year after the last subject last visit) the samples will be either destroyed or will be transferred to the Oxford Vaccine Centre (OVC) Biobank (only in the cases where consent to store the samples indefinitely and for future use has been provided by the participant). The OVC Biobank will then need to give approval for any samples to be extracted and used (so applications directly to the OVC Biobank will need to be made to access these samples). The future use of the stored samples will be limited to analyses related to rabies immunity and rabies vaccine development as described in the approved ICF. For additional studies beyond those described in the ICF, permission should also be obtained from relevant IRBs as described above.

Blood samples (serum, plasma, whole blood and PBMC) will be stored at IHI BRTC Laboratory in Bagamoyo, Tanzania and some will be shipped to the following collaborative laboratories;

- Jenner Institute laboratories, Centre for Clinical Vaccinology & Tropical Medicine, University of Oxford, Churchill Hospital, Old Road, Oxford, OX3 7LJ, United Kingdom.
- The Wistar Institute of Anatomy and Biology 3601 Spruce Street, Philadelphia, PA 19104 USA.

Samples shipped to these laboratories may then be sent to other specialist laboratories both within and outside Europe for secondary objective and/or exploratory immunology analysis related to rabies vaccine development as approved by the ethics committees. The transfer will only happen if there is no possibility of doing these analyses at IHI or University of Oxford laboratories.

At BRTC laboratory, freezer and refrigerator temperatures are recorded twice a day (in the morning and evening), through in-built system and thermometers. The temperature of the freezers will be recorded on temperature logs; in addition, temperature is also continuously recorded by a data logger.

7.8 Laboratory Evaluations

7.8.1 Safety Blood Assessments

Blood samples will be collected at specific time-points for haematological and biochemistry tests. The time points and the specific tests are shown in Appendix 1. Protocol specific tests will include the following;

- Complete Blood Count:
  Red blood count, haemoglobin, platelets, white blood count with differential (neutrophil, lymphocyte and eosinophil) counts.
- Biochemistry parameters:
- *At screening:* ALT and creatinine.
- *During subsequent scheduled visits:* ALT and creatinine.

During unscheduled visits, laboratory parameters to be tested will depend on the relevance as determined by the study clinician.

7.8.2 Urinalysis:

Urine sample will be collected at screening for urinalysis (blood, protein, glucose).

7.8.3 Malaria Microscopy:

The WHO malaria microscopy method will be adopted and used to identify (thick blood smear) malaria parasites in participants. Microscopy will be done at screening.

7.8.4 Serology

Blood samples for HIV, HBV and HCV serology will be done at the screening visit 1.

7.8.5 Urine Sample Pregnancy Test

For female adult participants (18-45 years), a urine pregnancy test will be performed at first screening and before each vaccination. Pregnant women will be excluded from participation in D0 vaccinations.

For participants who don’t consent to the extended follow-up period of 5.5 years, if pregnancy is diagnosed after vaccination, the participant will be excluded from further vaccination but will be followed up for safety until the end of the study. If possible, the outcome of the pregnancy will also be determined, including the health of the new-born.

For participants who consent to the extended follow-up period of 5.5 years, if pregnancy is diagnosed after vaccination and during the first year of follow up, the participant will be

excluded from further vaccination but will be followed up for safety until the end of the study. If possible, the outcome of the pregnancy will also be determined, including the health of the new-born.

For participants who consent to the extended follow-up period of 5.5 years, if pregnancy is diagnoses after the first year of follow up but before the end of the study, SPEP visits and the receipt of Verorab will be delayed as outlined in section 7.5.

7.9 Rationale for blood volume collected

As part of the process to address the research question, blood samples will be collected to assess both the safety and immunogenicity of ChAdOx2 RabG candidate rabies vaccine. It is however imperative to prioritise the safety and wellbeing of the study participants during the study as stipulated in the Declaration of Helsinki. In order to minimize risk to the study participants, the amount of blood volume that will be collected has been estimated using existing guidelines which recommend age-specific volume limits based on total blood volume, haemoglobin level and general health status of the donors [52]. The blood volume limits as recommended by WHO range from 1-5% of the total volume within 24 hours and up to 10% of total blood volume over 8 weeks [53]. A mean of 4% of TBV has been selected as the maximum blood draw limit over 24 hours in this trial. This is equivalent to around 3.2mL/Kg. A mean weight was estimated using the maximum and minimum normal weight limit at –2SD of WHO growth charts and a standard 70kg for young adults. Table show the estimated maximum allowable blood volume per visit and the planned maximum amount to be taken per visit. Appendix A shows the amount of blood that will be collected in each visit and the cumulative total amount of blood collected during the entire period of study.

Table 8: Maximum allowable blood volumes to be collected per visit.

Age group	Mean weight (Kg)	Maximum allowable blood volume per visit	Maximum blood volume that will be collected per visit (range)
Young children (2-6 years)	9	29mL	25 (3-25) mL
Adults (18-45 years)	70	140mL	101.5 (3-101.5) mL

7.10 Research Assays to Address Secondary endpoints

The blood volumes for the immunological outcomes and time points at which samples will be collected will depend on the group of the participants as shown in APPENDIX A: SCHEDULE OF PROCEDURES. Serum and/or plasma samples collected will be assessed by the rabies virus neutralisation assay at timepoints listing in Section 4.

All analyses will be conducted at the laboratories outlined in Section 7.7.

7.11 Study termination

The study may be terminated at any point, if the investigators and SMC are in agreement that the data obtained does not support the onward development of the product and that continuation would not be of substantial scientific benefit. If such a decision is taken, all volunteers would be invited to attend SPEP+0, SPEP+7 and SPEP+14 visits as soon as is practicable, and so would not be disadvantaged by not receiving acceptable PrEP with a licensed vaccine.

8 INVESTIGATIONAL MEDICINAL PRODUCT (IMP)

Participants will receive either one vaccination with ChAdOx2 RabG or vaccination with a currently available WHO prequalified IRV at two anatomical sites on 1 or 2 visits at the start of the trial.

All participants will also receive a currently available IRV 12 months after receipt of their first vaccination in the trial.

8.1 IMP Description

8.1.1 ChAdOx2 RabG

ChAdOx2 RabG has been developed and produced by the Jenner Institute, University of Oxford. The ChAdOx2 RabG vaccine consists of the replication-deficient simian adenovirus vector ChAdOx2, containing glycoprotein of rabies expressed from the strong CMV IE promoter.

ChAdOx2 RabG is in liquid formulation at a concentration of >1.1 × 10^{11} vp/mL. The drug product is filled into 2mL glass vials with a 13 mm grey bromobutyl rubber freeze-dry stopper (CE Marked, supplied by Adelphi Tubes) and a 13 mm aluminium seal. Vials will be labelled by the University of Oxford Clinical Biomanufacturing Facility in accordance with ICH requirements for labelling of IMPs. An example label is shown below. Note: This is only an example and general format, and the final labels are subject to change.

Figure 2: Example of Vaccine label

**CLINICAL TRIAL: RAB002**<br>**ChAdOx2 RabG Vaccine 0.35 mL (nominal)**<br>Solution for Injection<br>Vial contains XXX vp/mL<br>Vial No:____ For Intramuscular Injection<br>Lot no: 02N19-01 Store at -80°C<br>Volunteer no: ____ Expiry Date: DDMMMYY	**FOR CLINICAL TRIAL USE ONLY**<br>Chief Investigator: Dr Alexander Douglas<br>University of Oxford,<br>CCVTM, Old Road, Oxford. OX3 7LE<br>Tel: 01865 287796<br>Fax: 01865 289694

8.1.2 Inactivated rabies vaccine

VERORAB, a WHO-prequalified inactivated rabies vaccine (IRV) will be used as a comparator. VERORAB is supplied as a liquid of Lyophilized + 0.4% Sodium Chloride in ampoule diluent.

Verorab is the first-choice IRV for the study and is available as of November 2022, but disruption to supply of IRVs has occurred in the recent past. In the event that Verorab becomes unavailable during the study, another WHO-prequalified IRV will be substituted. To be clear, although the word ‘Verorab’ is used for clarity throughout this document, the investigators may use another pre-qualified IRV interchangeably if Verorab is unavailable.

IRVs will be presented in the supplier's packaging, with regulator-approved labelling and information.

8.2 Storage of IMP

Long-term storage of the ChAdOx2 RabG IMP is between –70°C and –90°C in a locked freezer at the Clinical Biomanufacturing Facility, University of Oxford. The vaccines will be shipped from Oxford on dry ice, and then stored in a –70°C freezer at the BRTC laboratory until required. Vaccine accountability, storage, shipment and handling will be in accordance with relevant local standard operating procedures (SOPs) and forms. All movement of vaccines will be documented in vaccine accountability logs according to local site SOPs.

ChAdOx2 RabG will be stored in –70°C freezer at CTF. They will be thawed at CTF and used within 1 hour of thawing if kept room temperature. Thawed vaccine may be stored in a 2 to 8°C fridge or in a wet ice box for up to 12 hours.

Currently available IRVs will be acquired locally by the PI and stored according to the manufacturer's instructions.

The IMPs will be handled according to the relevant SOPs. ChAdOx2 RabG is a genetically modified organism (GMO). In order to minimise dissemination of the recombinant vectored vaccine virus into the environment, the inoculation site will be covered with a dressing after vaccination. This should absorb any virus that may leak out through the needle track and will be removed from the injection site after 30 minutes and disposed of according to local SOPs. Vaccine administrators and destructors will follow precautions for the safe handling of GMOs (including the use of eye protection and gloves).

8.3 Accountability of the Investigational Product

At all times the figures on supplied, used and remaining vaccine doses should match. At the end of the study, it must be possible to reconcile delivery records with those of used and unused stocks. An explanation must be given of any discrepancies. The study pharmacist will ensure accurate records are maintained with regard to the date the vaccine is received, manufacture date, lot numbers, quantity received and disposition of the vaccine according to the relevant SOP. The Sponsor (Oxford University) will also maintain a copy of the records held at the site including Study ID numbers, date and time the IMP has been administered, lot number and signature of the person administering the IMP. All unused vaccines will be returned to Jenner Institute at the end of the trial or destroyed if requested.

8.4 Concomitant Medication

Collection of information regarding concomitant medications will be done at each visit throughout the immunization period until 30 days after the ChAdOx2 RabG or IRV vaccination on day 0.

In the event of adverse events, participants will be assessed by the study clinician and medication provided as required, in accordance the Tanzania Ministry of Health, World Health Organization and/or international guidelines.

8.5 Post-trial treatment

As a preventative vaccine study in healthy volunteers, there will be no provision of the IMPs beyond the trial period.

9 SAFETY REPORTING

9.1 Definitions

Adverse Event (AE)	Any untoward medical occurrence in a participant to whom a medicinal product has been administered, including occurrences which are not necessarily caused by or related to that product.
Adverse Reaction (AR)	An untoward and unintended response in a participant to an investigational medicinal product which is related to any dose administered to that participant.<br>The phrase "response to an investigational medicinal product" means that a causal relationship between a trial medication and an AE is at least a reasonable possibility, i.e. the relationship cannot be ruled out.<br>All cases judged by either the reporting medically qualified professional or the Sponsor as having a reasonable suspected causal relationship to the trial medication qualify as adverse reactions.
Serious Adverse Event (SAE)	A serious adverse event is any untoward medical occurrence that:<br>• results in death<br>• is life-threatening<br>• requires inpatient hospitalisation or prolongation of existing hospitalisation<br>• results in persistent or significant disability/incapacity<br>• consists of a congenital anomaly or birth defect.<br>Other ‘important medical events’ may also be considered serious if they jeopardise the participant or require an intervention to prevent one of the above consequences.<br>NOTE: The term "life-threatening" in the definition of "serious" refers to an event in which the participant was at risk of death at the time of the event; it does not refer to an event which hypothetically might have caused death if it were more severe.
Serious Adverse Reaction (SAR)	An adverse event that is both serious and, in the opinion of the reporting Investigator, believed with reasonable probability to be due to one of the trial treatments, based on the information provided.

Suspected Unexpected Serious Adverse Reaction (SUSAR)	A serious adverse reaction, the nature and severity of which is not consistent with the information about the medicinal product in question set out:<br>• in the case of a product with a marketing authorisation, in the summary of product characteristics (SmPC) for that product<br>• in the case of any other investigational medicinal product, in the investigator's brochure (IB) relating to the trial in question.

NB: to avoid confusion or misunderstanding of the difference between the terms "serious" and "severe", the following note of clarification is provided: "Severe" is often used to describe intensity of a specific event, which may be of relatively minor medical significance. "Seriousness" is the regulatory definition supplied above.

Any pregnancy occurring during the clinical trial and the outcome of the pregnancy should be recorded and followed up for congenital abnormality or birth defect, at which point it would fall within the definition of "serious".

9.2 Causality

The causal relationship between the AE and the product will be evaluated by the PI. This interpretation will be based on the type of event, the relationship of the event to the time of vaccine administration, and the known biology of vaccine therapy. The following are guidelines for assessing the causal relationship:

No relationship:

- No temporal relationship to study product; and
- Alternate aetiology (clinical state, environmental or other interventions); and
- Does not follow known pattern of response to study product

Unlikely relationship

- Unlikely temporal relationship to study product; and
- Alternate aetiology likely (clinical state, environmental or other interventions); and
- Does not follow known typical or plausible pattern of response to study product

Possible relationship:

- Reasonable temporal relationship to study product; or
- Event not readily produced by clinical state, environmental or other interventions; or
- Similar pattern of response to that seen with other vaccines

Probable relationship:

- Reasonable temporal relationship to study product; and
- Event not readily produced by clinical state, environment, or other interventions or
- Known pattern of response seen with other vaccines

Definite relationship:

- Reasonable temporal relationship to study product; and
- Event not readily produced by clinical state, environment, or other interventions; and
- Known pattern of response seen with other vaccines

When a regulatory authority requests distinct classification of AEs into either related or unrelated, without intermediate categories, only “not related” will be regarded as “unrelated” and “unlikely related”, “possibly related,” “probably related,” and “definitely related” will be combined as “related”.

9.3 Procedures for Recording Adverse Events

9.3.1 Recording adverse events

Investigators will evaluate all adverse events observed or reported by the participant or their parents/guardians at each scheduled or unscheduled visit. New adverse events will be recorded in the Adverse Event form within the participant’s CRF. Solicited adverse events will be recorded on separate pages of the CRF. The nature of each event, date of onset, outcome, intensity and relationship to vaccination will be established.

Any corrective treatment will be recorded in the CRF as concomitant medication. For solicited adverse events participants or their parents/guardians will be asked direct questions related to the pre-listed symptoms. For unsolicited adverse events, participants or their parents/guardians will be asked an open-ended question such as: “Have you felt different in any way since receiving the vaccine or since the last visit?” The investigator will record only those adverse events having occurred within the time frames defined above.

Adverse events already documented in the CRF, i.e. at a previous assessment and designated as ‘ongoing’ will be reviewed at subsequent visits, as necessary. If these events have resolved, the documentation in the CRF will be completed, including the date that the adverse event resolved. If an adverse event changes in frequency or intensity during a study period, the record will be updated to reflect the maximum intensity or describe the frequency.

9.3.2 Solicited Systemic AEs

For all participants, a prescribed list of systemic AEs will be solicited on the first day of vaccination (day 0) through to 7 days post vaccination (see Table). During these periods all participants will be examined daily for elevated body temperature and allergic reaction (rash, urticaria, pruritus, and/or oedema). Additional AEs occurring in adults will be

solicited by history from a list of systemic AEs (see Table) while the guardians of young children will be asked about observed history of fever, vomiting, diarrhoea, reduced oral intake, and reduced activities (see Table).

9.3.3 <u>Solicited Local AEs:</u>

A set list of local AEs will be solicited from all participants after each injection see Table). For young children answers to pain, tenderness and pruritus may not be able to be obtained. Local AEs will be solicited on the first day of vaccination (day 0) and 7 days following each vaccination.

9.3.4 <u>Unsolicited AEs</u>

Unsolicited AEs will be conducted up until day 28 after the first vaccination on day 0. A syndromic classification will be used for unsolicited AEs, e.g., cough, nasal congestion, sore throat should be combined into upper respiratory tract infection. Thus, the term for the unifying diagnosis is recorded as the AE, not each individual sign or symptom whenever applicable.

Table 9: Solicited Adverse Events

Local Solicited AEs *(at injection site)*	• Pain		0	No pain
			1	Painful on touch, no restriction on movement of limb (baby cries when limb is touched but moves the limb around without restriction)
			2	Painful when arm is moved (baby cries when limb moved)
			3	Unable to use the limb due to pain
	• Itch		0	No symptom/sign
			1	Awareness of a symptom, but the symptom is easily tolerated and causes no or minimal interference with usual activity.
			2	Discomfort enough to cause greater than minimal interference with usual activity.
			3	Incapacitating; symptoms causing inability to perform usual activities; requires medical intervention
	<u>Children</u>	• Redness<br>• Swelling<br>• Induration	0	0
			1	<20 mm
			2	20-50 mm
			3	>50 mm and/or necrosis or exfoliative dermatitis
	<u>Adults</u>	• Redness<br>• Swelling<br>• Induration	0	0
			1	<50 mm
			2	50 – 100 mm
			3	>100 mm and/or necrosis or exfoliative dermatitis
Systemic Solicited AEs	• Fever (objective)			
			1	37.6°C – 38.4°C
			2	38.5°C – 39°C
			3	>39.0°C
	<u>Adults</u>	• Feverishness (subjective fever / chills)<br>• Joint pains<br>• Muscle pains<br>• Fatigue	0	No symptom/sign

		• Headache<br>• Nausea<br>• Malaise<br>• Allergic reaction *(rash, urticaria, pruritis, oedema)*	1	Awareness of a symptom, but the symptom is easily tolerated and causes no or minimal interference with usual activity.
	Infants and young children	• Allergic reaction *(rash, urticaria, pruritis, oedema)*<br>• Subjective fever<br>• Vomiting<br>• Diarrhoea<br>• Reduced activities<br>• Reduced oral intake		
			2	Discomfort enough to cause greater than minimal interference with usual activity.
			3	Incapacitating; symptoms causing inability to perform usual activities; requires absenteeism or bed rest

9.3.5 Follow-up of Adverse Events

All AEs will be followed until resolution of the signs or symptoms or laboratory changes occurs, or until a non-study related causality is assigned.

At the study end, if participants have moderate or severe on-going adverse events not considered to be related to the study vaccine, they will be advised to consult a government health facility. If considered to be related to the study vaccine, a follow-up visit will be arranged to manage the problem and to determine the severity and duration of the event. If appropriate, specialist review will be arranged by Investigators.

Any serious adverse event possibly related to the vaccine and occurring after termination of the clinical trial should be reported by the Investigator according to the procedure described below.

9.3.6 Grading the severity of adverse events

For adverse events other than local swelling, redness/discoloration and pain/limitation of limb movement, for which the severity scales are detailed above, AEs will be graded according to the relevant grading tables[54, 55]. These internationally recognized grading tables classify adverse events into one of four grades, ranging from mild to potentially life-threatening. The grading tables have indications for each of over sixty clinical parameters and forty laboratory parameters for grading adult and paediatric AEs. The table also includes general guidelines for estimating the grade of parameters not explicitly listed. Each grade is described broadly below:

- Grade 1 (mild): awareness of a symptom, but the symptom is easily tolerated and causes no or minimal interference with usual activity.
- Grade 2 (moderate): discomfort enough to cause greater than minimal interference with usual activity.
- Grade 3 (severe): incapacitating; symptoms causing inability to perform usual activities; requires absenteeism or bed rest.

- Grade 4 (potentially life-threatening): symptoms causing inability to perform basic self-care functions OR medical or operative intervention is indicated to prevent permanent impairment, persistent disability or death.

Laboratory tests will also be graded based on the relevant toxicity grading scales relevant to healthy HIV negative volunteers with modification to suit local or regional normal values. In addition to the referenced toxicity grading scales above, any other relevant toxicity grading scale may be used to supplement and accomplish proper assessment. Toxicity grades and reference scales which will be used to grade severity of laboratory solicited AEs for this trial will be described in a relevant study SOPs. The abnormal values will be categorized as either clinically significant or non-clinically significant based on the study physician's medical judgement. Abnormal laboratory assessments that are judged by the Investigator to be serious will be recorded as SAEs.

9.4 Reporting Procedures for Serious Adverse Events

In order to comply with current regulations on serious adverse event reporting to regulatory authorities, the event will be documented accurately, and notification deadlines respected. SAEs will be reported on the standard SAE forms to members of the study team immediately when the investigators become aware of their occurrence. Copies of all reports will be forwarded by email (sae@well.ox.ac.uk) for review to the Chief Investigator (as the Sponsor's representative) within 24 hours of the Investigator being aware of the suspected SAE. The SMC will be notified immediately by the PI if SAEs are deemed possibly, probably or definitely related to study interventions; in such a situation, the PI will notify the SMC immediately within 24 hours of the PI being aware of their occurrence.

SAEs will not normally be reported immediately to the OxTREC unless there is a clinically important increase in occurrence rate, an unexpected outcome, or a new event that is likely to affect safety of trial volunteers, at the discretion of the Chief Investigator and/or SMC. Sponsor will notify The Oxford Tropical Research Ethics Committee (OxTREC) of all SAE that are deemed possibly, probably or definitely related to study interventions and SUSARs within specified time-frame as required by the regulations.

All SAEs will be summarized and reported to IHI IRB and The National Health Research Ethics Sub-Committee (NatHREC) in the required specified time-frames. The PI, on behalf of the Sponsor, will also report all SAEs to the Tanzanian Medicines and Medical Devices Authority (TMDA) within specified time-frame as required by the regulations.

Additional or follow-up information (outcome, precise description of medical history, results of the investigation, copy of hospitalisation report, etc.) relating to the initial SAE report will also be reported to the Sponsor and SMC within 24 hours of receipt of such information.

9.4.1 <u>Reporting Procedures for SUSARs</u>

All SUSARs will be reported by the CI to the relevant Competent Authority and to the Ethics Committee and other parties as applicable. For fatal and life-threatening SUSARS, this will be done no later than 7 calendar days after the Sponsor or delegate is first aware of the reaction. Any additional relevant information will be reported within 8 calendar days of the initial report. All other SUSARs will be reported within 15 calendar days.

Principal Investigators will be informed of all SUSARs for the relevant IMP for all studies with the same Sponsor, whether or not the event occurred in the current trial.

The Chief Investigator (on behalf of The Sponsor) will report all SUSARs to the ethical committee(s) and Tanzanian regulatory authorities within required timelines. The Chief Investigator will also inform all Investigators concerned of relevant information about SUSARs that could adversely affect the safety of participants.

For all deaths, available autopsy reports and relevant medical reports will be made available for reporting to the relevant authorities.

9.5 Safety Monitoring Committee

For this study, an independent SMC will be appointed by the UOXF (Sponsor) to follow up the safety of participants as described above and in section 5.3. The SMC's composition, responsibilities and procedures will be pre-specified by a safety monitoring charter.

In brief, the SMC will be composed of 3 members one of whom one will be a qualified Tanzanian paediatrician. All of the members will be experienced clinicians qualified to evaluate safety data from clinical trials. The SMC will carry out a sequence of reviews to decide whether the trial will first commence and subsequently to provide clearance on allowing the trial to progress through groups (detailed in section 5.3.2). They will also take part in the assessment of adverse events (if requested) and any recommendation regarding halting further vaccination. The SMC will liaise closely with the PI throughout the course, mutually relaying relevant safety information.

In addition to the scheduled reviews, SMC may be contacted for advice and independent review by the Sponsor, CI or PI, as in the following situations:

- AEs in the sentinel participants trigger criteria for an ad hoc SMC review
- Following any SAE deemed to be possibly, probably, or definitely related to the study vaccine.
- Any other situation where the PI, CI or Sponsor thinks independent advice or review is important.

Only SAEs deemed possibly, probably or definitely related to study interventions will be reported to the SMC by the PI, unless an SMC opinion on an unrelated SAE is felt, by the

PI, to be desirable. If required, the SMC may provide a separate written report to document their assessment of the SAE or other concerning AE.

9.6 Development Safety Update Reports

The CI will submit (in addition to the expedited reporting above) DSURs once a year throughout the clinical trial, or on request, to the Competent Authority, Ethics Committee, and Sponsor.

10 STATISTICAL ANALYSIS CONSIDERATION

All final analyses of clinical safety data will be performed by the PI with the support from the Sponsor and IHI statisticians. Final analysis of data relating to immunogenicity as part of the secondary and exploratory objective of this study will be carried out under the guidance of the CI and PI.

The primary safety analysis will be a descriptive analysis of the safety data, without any hypothesis testing or statistical inference, in all participants who have received ChAdOx2 RabG (safety dataset). The sample size has therefore been chosen on grounds of precedent (see section 3.11) and the opinion of investigators of what constitutes an adequate number of participants to facilitate further clinical evaluation in phase II trials.

Assessment of the immunogenicity of the vaccine is a secondary objective. Analyses of immunogenicity, based upon the induction of virus neutralising antibody (VNA), is pre-specified in a separate statistical analysis plan, signed and dated by the CI prior to the submission of the first sample set for VNA analysis, agreed upon by the PI and Sponsor. The analysis plan will provide information of the populations that will be analysed and how the safety and immunogenicity results will be presented as well as specific statistical methods that will be applied.

11 DATA HANDLING AND RECORD KEEPING

11.1 Data handling and record keeping

The PI will delegate to the data manager the responsibility of receiving, entering, cleaning, querying, analysing and storing all data that accrues from the study. Trained study staff will enter the data into the participants' CRFs, which will be in a paper format. These data will include clinical and laboratory safety data. All source documents and laboratory reports will be reviewed by the clinical team and by data entry staff, who will ensure that they are accurate and complete. Adverse events must be graded, assessed for severity and causality, and reviewed by the PI or designee.

The Investigators will maintain appropriate medical and research records for this trial in compliance with ICH E6 GCP and regulatory and institutional requirements for the protection of confidentiality of participants. The Investigators will permit authorized representatives of the Sponsor, regulatory agencies and the monitors to examine clinical records for the purposes of quality assurance reviews, audits and evaluation of the study safety and progress.

Data and samples collected will be provided to the Sponsor to allow study related documentation and immunological analyses where these analyses cannot be done in Tanzania. Data and sample shipment agreements will be established between the Sponsor and the IHI before samples and data are transferred to the Sponsor.

Study documents will be retained for a minimum of 20 years after the end of the clinical trial. These documents will be retained for a longer period, however, if required by local regulations. No records will be destroyed without the written consent of Sponsor and it is the responsibility of Sponsor to inform the investigator when these documents no longer need to be retained.

11.2 Access to Data

Direct access will be granted to authorised representatives from the Sponsor, host institution and the ethical and regulatory authorities to permit trial-related monitoring, audits and inspections and evaluation of the study safety, progress, and data validity. All documents will be stored safely in a locked filing cabinet where only study Investigators will have access to the keys.

11.3 Source document and Case Report Form

All protocol-required information will be collected in CRFs designed by the investigator. All source documents will be filed in the CRF. Source documents are original documents, data, and records from which the volunteer's CRF data are obtained. For this study these will include, but are not limited to; volunteer consent form, blood results, clinician's medical notes, laboratory records, and relevant correspondences. In the majority of cases, CRF

entries will be considered source data as the CRF is the site of the original recording (i.e. there is no other written or electronic record of data). In this study collected information in the CRF will include, but is not limited to medical history, medication records, vital signs, physical examination records, urine assessments, blood, urine analysis results, adverse event data and details of vaccinations. If participants fell ill and receive medical treatment, medical notes and investigational results will also be considered as source documents. All source data and participant CRFs will be stored in a secure place accessible to only authorized study staff.

Data entry into the USA FDA 21 CFR Part 11-compliant Electronic Data Capture system will be done locally by study staff. The data system will include password protection and internal quality checks, such as automatic range checks, to identify data that appear inconsistent, incomplete, or inaccurate. Clinical data will be entered directly from the source documents. Immunology data will be generated from analyses of blood samples and will be stored in Excel format for sharing and archiving, and analysed using statistical packages (eg GraphPad Prism, and/or STATA). The name and any other identifying detail will NOT be included in any trial data electronic file. On all trial-specific documents, other than the signed consent, the participant will be referred to by the trial Study ID number, not by name.

12 QUALITY ASSURANCE PROCEDURES

The trial will be conducted in accordance with the current approved protocol, GCP, relevant regulations and standard operating procedures.

12.1 Monitoring

Clinical trial monitoring will be conducted jointly by IHI QA team, with support from the Kenya Medical Research Institute (KEMRI), according to applicable local SOPs to ensure that GCP standards and regulatory guidelines are being followed. Pre-trial monitoring visits will be made to the site, including the clinical laboratory. All records will be made available to monitors, including regulatory files, CRFs and other source documents, QA/QC documentation, SOPs, etc.

A detailed monitoring plan, subject to approval by the sponsor, will be developed by the IHI QA team in collaboration with KEMRI-Wellcome Trust monitors. The monitoring plan will include the number of participant charts to be reviewed, which/what proportion of data fields and what will be monitored, who will be responsible for conducting the monitoring visits, and who will be responsible for ensuring that monitoring findings are addressed.

12.2 Investigator procedures

Approved site-specific SOPs will be used at all clinical and laboratory sites.

12.3 Modification to protocol

No amendments to this protocol will be made without consultation with, and agreement of, the Sponsor. Any amendments to the trial that appear necessary during the course of the trial must be discussed by the Investigator and Sponsor concurrently. If agreement is reached concerning the need for an amendment, it will be produced in writing by the PI and will be made a formal part of the protocol following ethical and regulatory approval.

An administrative change to the protocol is one that modifies administrative and logistical aspects of a protocol but does not affect the subjects' safety, the objectives of the trial and its progress. An administrative change does not require ethical or regulatory approval.

The Investigator is responsible for ensuring that changes to an approved trial, during the period for which regulatory and ethical approval has already been given, are not initiated without regulatory and ethical review and approval except to eliminate apparent immediate hazards to the subject.

12.4 Protocol deviation

A protocol deviation is any noncompliance with the clinical trial protocol, GCP, or SOP requirements. The noncompliance may be either on the part of the participant, the

investigator, or the study site staff. As a result of deviations, corrective actions are to be developed by the site and implemented promptly.

It is the responsibility of the site to use continuous vigilance to identify and report deviations within 5 working days of identification of the protocol deviation, or within 5 working days of the planned protocol-required activity. All deviations must be promptly reported to the Sponsor.

Any deviations that impact subject safety, or that alter the risk: benefit analysis or the scientific integrity of the study is regarded as major deviation and will be reported to the Sponsor within 24 hours of the PI or study personnel becoming aware of the deviation. Major deviation will be reported to the Tanzanian regulatory authorities by the PI within 7 days of the Sponsor becoming aware of the deviation.

All deviations from the protocol must be addressed and a completed copy of the Protocol Deviation Form must be maintained in the trial master file, as well as in the participant’s source document if necessary. Protocol deviations will be reported to the IHI-IRB, NatHREC and TMDA per their guidelines.

12.5 Audit & inspection

The QA designated site staff will conduct internal audits to check that the trial is being conducted, data recorded, analysed and accurately reported according to the protocol, Sponsor’s SOPs and in compliance with ICH GCP. The audits will also include laboratory activities according to an agreed audit schedule.

The Sponsor may carry out audit to ensure compliance with the protocol, GCP and appropriate regulations. GCP inspections may also be undertaken by the regulatory authority to ensure compliance with protocol and national regulations. The Sponsor will assist in any inspections.

12.6 Study results feedback

Study outcomes will be shared with study participants after the final safety report is completed in the meeting that will be organized by the study team. The summary of safety results will be provided to the volunteers and their parents. No personal information will be disclosed. In addition, local authorities will be informed of the end of the study. The final safety report will be submitted to IHI IRB, NatHREC and TMDA. In addition, the results will be shared with the scientific community and other stakeholders involved in the fight against Rabies in Tanzania and globally.

13 ETHICAL AND REGULATORY CONSIDERATIONS

13.1 Declaration of Helsinki

The Investigator will ensure that this trial is conducted in accordance with the principles of the Declaration of Helsinki 2008.

13.2 Guidelines for Good Clinical Practice

The Investigator will ensure that this trial is conducted in accordance with Medicine for Human use (clinical trials) Regulations 2004 and its amendments and with the ICH guidelines for GCP (CPMP/ICH/135/95) July 1996. The trial will also comply with the European Communities (Clinical Trials on Medicinal Products for Human Use) Regulations, 2004 [S.I. 190 of 2004].

13.3 Approvals

The protocol, informed consent form, and participant information sheet will be submitted to IHI IRB, National Health Research Ethics Sub-Committee (NatHREC) and Oxford Tropical Research Ethics Committee (OxTREC) for written approval. In addition, approval from Tanzania Medicine & Medical Devices Authority Home (TMDA), a regulatory authority, will be sought before the study starts. No data collection will start without approval from ethics committees and the regulatory authority.

The Investigator will submit and, where necessary, obtain approval from the above parties for all substantial amendments to the original approved documents.

13.4 Reporting

The PI shall submit a progress report every six months throughout the clinical trial, or on request, to the IHI IRB, National Health Research Ethics Sub-Committee (NatHREC), Oxford Tropical Research Ethics Committee (OxTREC), Tanzania Medicines and Medical Devices Authority (TMDA) and Sponsor. In addition, an End of Trial notification and final report will be submitted to the IHI IRB, National Health Research Ethics Sub-Committee (NatHREC), Oxford Tropical Research Ethics Committee (OxTREC), Tanzania Medicines and Medical Devices Authority (TMDA) and Sponsor.

13.5 Participant Confidentiality

The trial staff will ensure that the participants' anonymity is maintained. The participants will be identified only by a participant study ID on all trial documents and any electronic database, with the exception of the CRF, where participant initials may be added. All documents will be stored securely and only accessible by trial staff and authorised personnel, including representatives of the Research Ethics Committees and regulatory

authorities. The trial will comply with the Data Protection Act, 1998, which requires data to be anonymised as soon as it is practical to do so.

Photographs taken of vaccination sites (if required, with the volunteer's written, informed consent) will not include the volunteer's face and will be identified by the date, trial code and subject's unique identifier. Once developed, photographs will be stored as confidential records, as above. This material may be shown to other professional staff, used for educational purposes, or included in a scientific publication.

13.6 Potential Benefits to participants

Participants will benefit from receiving a free consultation and information about their general health status at screening. This information will help participants get medical attention as soon as possible to avoid potential complications. In addition, all participants will receive a currently available IRV which will protect them against rabies in case they are bitten/scratched by a rabid animal. A stock of currently available IRV will also be kept to assist participants who received IMP in case they are bitten/scratched by a rabid animal prior to receipt of the IRV according to the study schedule. During the trial, all participants will receive information about their health status. Medical costs for acute illnesses whether or not related to the IMP or study procedure during the study period will be covered by the study to the limit of the allocated funds. When experiencing illness, enrolled participants will receive care at Bagamoyo District Hospital. If the situation arises in which medical care for a complication unrelated to the study procedure/product exceeds the limits of the budget allocated for clinical care, the participant or guardian will be responsible for funding this care through the normal channels provided by the Ministry of Health and Social Welfare of United Republic of Tanzania.

13.7 Potential Risks and Burden to Participants

13.7.1 Vaccination:

Risks for participants are related to exposure to the ChAdOx2 vector and to the RabG antigen expressed by the vector. Potential expected risks from vaccination, which include local and systemic reactions are specific to each IMP and are described in section 9. It is important to note that ChAdOx2 RabG has not previously been administered to young children but will have been safely administered in UK adults prior to this trial commencing. Other simian adenovirus vectored vaccines have been safety administered in infants and young children [40]. Therefore, although the AE profile can be estimated from previous use of these vectors, the reactogenicity may vary from that seen previously with. For this reason, vaccinees will be enrolled in a staggered format to allow early identification of any concerning reactogenicity before the majority of individuals have been vaccinated. Any vaccine can cause allergic reactions including anaphylaxis. Therefore, vaccination will take place in the presence of Advanced Life Support trained physicians and where equipment and drugs for managing anaphylaxis and any other severe adverse reaction are available.

13.7.2 Phlebotomy:

It is expected that participants will experience pain of a needle during vaccination and blood drawing at different time-points. The maximum volume of blood drawn over the study period will be based on age and weight and is not expected to compromise the health of participants. There may be minor bruising, local tenderness or pre-syncopal symptoms associated with venepuncture. This will be minimized by using experienced and trained clinical nurses.

13.8 Incentives

The study will cover all the costs related to participant's participation in the trial including clinic visits or hospitalization. A reasonable time reimbursement of 20 000 Tanzanian shillings will be provided during scheduled visits, including any visits related to long-term participant retention. In addition, participants will receive a meal during the scheduled visits. In case of expected or unexpected medical complications, medical treatment at CTF and BDH will be available at no cost to the participant. Participants who come for unscheduled visits will be reimbursed for their transport fare. In the event it is required, additional insurance coverage for the costs of medical treatment for acute illnesses or adverse events related to investigational product will be covered by liability insurance held by Oxford University.

13.9 Future use of stored samples

If residual sera and cells are available following the serological and immunogenicity assays described in this protocol, additional immunological and *in vitro* studies may be performed at the Bagamoyo Research and Training Centre laboratory in Tanzania on those samples for which permission was expressly granted for storing the samples for future studies at the time of informed consent. Study participants will have the right to withdraw their permission for further use of their samples at any time during and after the study.

The samples that are sent to UK will be stored at the Jenner Institute and used for analyses as outlined in the protocol, including at other laboratories within and outside Europe. At the end of the study (defined as one year after the last subject last visit), samples which have been shipped to the UK will be either destroyed, or stored in the Oxford Vaccine Centre (OVC) Biobank if the consent to store the sample indefinitely has been granted by the participant. Additional analyses related to vaccine development can be done on these samples after receiving approval from the Biobank.

Maximizing the research use of collected samples from participants to benefit science and society is an important ethical consideration. It is important to make efficient use of participant samples in an ethical manner with respect and transparency rather than collecting new samples where possible [56-58].

14 FINANCE AND INSURANCE

14.1 Funding

The study will be funded primarily by a grant from the Medical Research Council, UK.

14.2 Insurance

Negligent Harm: Indemnity and/or compensation for negligent harm arising specifically from an accidental injury for which Ifakara Health Institute is legally liable will be covered by Ifakara Health Institute.

Non-Negligent Harm: Indemnity and/or compensation for harm arising specifically from an accidental injury and occurring as a consequence of the Research Subjects' participation in the trial for which the University is the Research Sponsor will be covered by the Ifakara Health Institute and University of Oxford (the University has a specialist insurance policy in place with Newline Underwriting Management Ltd, at Lloyd’s of London, UK). In addition, compensation for injury will be guided by the respective insurance policies that adhere to the national guidelines provided by the TMDA.

15 PUBLICATION POLICY

The Final Safety Report will be prepared by the PI. The protocol and data derived from the trial are the shared property of the IHI and the University of Oxford University. Any publication or presentation, abstracts and press releases related to the trial must be approved by all parties' representatives before submission of the manuscript. After publication of the results of the trial, any participating partner may publish or otherwise use its own data provided that any publication of data from the trial gives recognition to the trial group. Either partner must have the opportunity to review the proposed abstract, manuscript or presentation before submission for publication/presentation. A request for delay in publication shall only be allowed to enable the requesting party to secure its proprietary and confidential information contained in the draft publication and/or to prevent the loss of patenting opportunities. Such delay shall not be more than 3 months starting from the day of submission for approval. Any information identified as confidential must be deleted prior to submission. The authorized persons as an author of the publication(s) are those who have contributed to the protocol and/or to the analysis of the data, drafting and reviewing the communication. According to the main topic of the publication, the first author will be the greatest contributing Investigator and the CI the senior author. Data from the study may also be used as part of a thesis for a PhD, MD or Masters. Publications arising from this study will be made open access.

16 REFERENCES

1. Fooks AR, Cliquet F, Finke S, Freuling C, Hemachudha T, Mani RS, et al. Rabies. Nature Reviews Disease Primers. 2017;3:17091. doi: 10.1038/nrdp.2017.91

https://www.nature.com/articles/nrdp201791#supplementary-information.

2. World Health Organization. WHO Expert Consultation on Rabies. Third report. World Health Organization technical report series. 2018;(1012):1-184, back cover.

3. Changalucha J, Steenson R, Grieve E, Cleaveland S, Lembo T, Lushasi K, et al. The need to improve access to rabies post-exposure vaccines: Lessons from Tanzania. Vaccine. 2018. Epub 2018/10/13. doi: 10.1016/j.vaccine.2018.08.086. PubMed PMID: 30309746.

4. Hampson K, Coudeville L, Lembo T, Sambo M, Kieffer A, Attlan M, et al. Estimating the global burden of endemic canine rabies. PLoS neglected tropical diseases. 2015;9(4):e0003709. Epub 2015/04/17. doi: 10.1371/journal.pntd.0003709. PubMed PMID: 25881058; PubMed Central PMCID: PMCPMC4400070.

5. Cleaveland S, Fèvre EM, Kaare M, Coleman PG. Estimating human rabies mortality in the United Republic of Tanzania from dog bite injuries. Bull World Health Organ. 2002;80(4):304-10. PubMed PMID: 12075367.

6. Hampson K, Dobson A, Kaare M, Dushoff J, Magoto M, Sindoya E, et al. Rabies Exposures, Post-Exposure Prophylaxis and Deaths in a Region of Endemic Canine Rabies. PLOS Neglected Tropical Diseases. 2008;2(11):e339. doi: 10.1371/journal.pntd.0000339.

7. O'Brien KL, Nolan T. The WHO position on rabies immunization - 2018 updates. Vaccine. 2018;Epub ahead of print. Epub 2018/10/22. doi: 10.1016/j.vaccine.2018.10.014. PubMed PMID: 30342901.

8. World Health Organization. Rabies vaccines: WHO position paper, April 2018 - Recommendations. Vaccine. 2018;36(37):5500-3. Epub 2018/08/16. doi: 10.1016/j.vaccine.2018.06.061. PubMed PMID: 30107991.

9. Jonker EFF, Visser LG. Single visit rabies pre-exposure priming induces a robust anamnestic antibody response after simulated post-exposure vaccination: results of a dose-finding study. Journal of travel medicine. 2017;24(5). Epub 2017/09/21. doi: 10.1093/jtm/tax033. PubMed PMID: 28931127.

10. Khawplod P, Jaijaroensup W, Sawangvaree A, Prakongsri S, Wilde H. One clinic visit for pre-exposure rabies vaccination (a preliminary one year study). Vaccine. 2012;30(19):2918-20. Epub 2011/12/20. doi: 10.1016/j.vaccine.2011.12.028. PubMed PMID: 22178519.

11. Soentjens P, De Koninck K, Tsoumanis A, Herssens N, Van Den Bossche D, Terryn S, et al. A Comparative Immunogenicity and Safety Trial of Two Different Schedules of Single-visit Intradermal Rabies Post-exposure Vaccination Following a Single-visit Pre-exposure Vaccination. Clin Infect Dis. 2018;Epub ahead of print. Epub 2018/12/20. doi: 10.1093/cid/ciy983. PubMed PMID: 30566636.

12. Shantavasinkul P, Tantawichien T, Jaijaroensup W, Lertjarutorn S, Banjongkasaena A, Wilde H, et al. A 4-site, single-visit intradermal postexposure prophylaxis regimen for previously vaccinated patients: experiences with >5000 patients. Clin Infect Dis. 2010;51(9):1070-2. Epub 2010/10/05. doi: 10.1086/656585. PubMed PMID: 20887204.

13. Tantawichien T, Benjavongkulchai M, Limsuwan K, Khawplod P, Kaewchompoo W, Chomchey P, et al. Antibody response after a four-site intradermal booster vaccination with cell-culture rabies vaccine. Clin Infect Dis. 1999;28(5):1100-3. Epub 1999/08/19. doi: 10.1086/514737. PubMed PMID: 10452642.

14. Lembo T, Hampson K, Kaare MT, Ernest E, Knobel D, Kazwala RR, et al. The feasibility of canine rabies elimination in Africa: dispelling doubts with data. PLoS neglected tropical diseases. 2010;4(2):e626. Epub 2010/02/27. doi: 10.1371/journal.pntd.0000626. PubMed PMID: 20186330; PubMed Central PMCID: PMCPMC2826407.

15. Control GAfR. Policy and Advocacy: Global Alliance for Rabies Control; 2015 [cited 2019 17 January 2019]. Available from: https://rabiesalliance.org/policy.

16. World Health Organization. Rabies: rationale for investing in the global elimination of dog-mediated human rabies. 2015.

17. Chulasugandha P, Khawplod P, Havanond P, Wilde H. Cost comparison of rabies pre-exposure vaccination with post-exposure treatment in Thai children. Vaccine. 2006;24(9):1478-82. Epub 2005/10/14. doi: 10.1016/j.vaccine.2005.03.059. PubMed PMID: 16221511.
18. Wang C, Dulal P, Zhou X, Xiang Z, Goharriz H, Banyard A, et al. A simian-adenovirus-vectored rabies vaccine suitable for thermostabilisation and clinical development for low-cost single-dose pre-exposure prophylaxis. PLoS neglected tropical diseases. 2018;12(10):e0006870. doi: 10.1371/journal.pntd.0006870.
19. Xiang ZQ, Greenberg L, Ertl HC, Rupprecht CE. Protection of non-human primates against rabies with an adenovirus recombinant vaccine. Virology. 2014;450-451(1096-0341 (Electronic)):243-9.
20. Bett AJ, Haddara W, Prevec L, Graham FL. An efficient and flexible system for construction of adenovirus vectors with insertions or deletions in early regions 1 and 3. Proc Natl Acad Sci U S A. 1994;91(19):8802-6. Epub 1994/09/13. PubMed PMID: 8090727; PubMed Central PMCID: PMCPMC44694.
21. Top FH, Jr., Buescher EL, Bancroft WH, Russell PK. Immunization with live types 7 and 4 adenovirus vaccines. II. Antibody response and protective effect against acute respiratory disease due to adenovirus type 7. J Infect Dis. 1971;124(2):155-60. Epub 1971/08/01. doi: 10.1093/infdis/124.2.155. PubMed PMID: 4330998.
22. Dudareva M, Andrews L, Gilbert SC, Bejon P, Marsh K, Mwacharo J, et al. Prevalence of serum neutralizing antibodies against chimpanzee adenovirus 63 and human adenovirus 5 in Kenyan children, in the context of vaccine vector efficacy. Vaccine. 2009;27(27):3501-4. Epub 2009/05/26. doi: 10.1016/j.vaccine.2009.03.080. PubMed PMID: 19464527.
23. Nwanegbo E, Vardas E, Gao W, Whittle H, Sun H, Rowe D, et al. Prevalence of neutralizing antibodies to adenoviral serotypes 5 and 35 in the adult populations of The Gambia, South Africa, and the United States. Clinical and diagnostic laboratory immunology. 2004;11(2):351-7. Epub 2004/03/12. PubMed PMID: 15013987; PubMed Central PMCID: PMCPMC371218.
24. Casimiro DR, Chen L, Fu TM, Evans RK, Caulfield MJ, Davies ME, et al. Comparative immunogenicity in rhesus monkeys of DNA plasmid, recombinant vaccinia virus, and replication-defective adenovirus vectors expressing a human immunodeficiency virus type 1 gag gene. J Virol. 2003;77(11):6305-13. Epub 2003/05/14. PubMed PMID: 12743287; PubMed Central PMCID: PMCPMC154996.
25. Kobinger GP, Feldmann H, Zhi Y, Schumer G, Gao G, Feldmann F, et al. Chimpanzee adenovirus vaccine protects against Zaire Ebola virus. Virology. 2006;346(2):394-401. Epub 2005/12/17. doi: 10.1016/j.virol.2005.10.042. PubMed PMID: 16356525.
26. Xiang Z, Gao G, Reyes-Sandoval A, Cohen CJ, Li Y, Bergelson JM, et al. Novel, chimpanzee serotype 68-based adenoviral vaccine carrier for induction of antibodies to a transgene product. J Virol. 2002;76(6):2667-75. Epub 2002/02/28. PubMed PMID: 11861833; PubMed Central PMCID: PMC135983.
27. Morris SJ, Sebastian S, Spencer AJ, Gilbert SC. Simian adenoviruses as vaccine vectors. Future virology. 2016;11(9):649-59. Epub 2016/09/01. doi: 10.2217/fvl-2016-0070. PubMed PMID: 29527232; PubMed Central PMCID: PMCPMC5842362.
28. Xiang Z, Li Y, Cun A, Yang W, Ellenberg S, Switzer WM, et al. Chimpanzee adenovirus antibodies in humans, sub-Saharan Africa. Emerging infectious diseases. 2006;12(10):1596-9. Epub 2006/12/21. doi: 10.3201/eid1210.060078. PubMed PMID: 17176582; PubMed Central PMCID: PMCPMC3290939.
29. Colloca S, Barnes E, Folgori A, Ammendola V, Capone S, Cirillo A, et al. Vaccine vectors derived from a large collection of simian adenoviruses induce potent cellular immunity across multiple species. Sci Transl Med. 2012;4(115):115ra2. Epub 2012/01/06. doi: 10.1126/scitranslmed.3002925. PubMed PMID: 22218691; PubMed Central PMCID: PMCPMC3627206.
30. Afolabi MO, Tiono AB, Adetifa UJ, Yaro JB, Drammeh A, Nebie I, et al. Safety and Immunogenicity of ChAd63 and MVA ME-TRAP in West African Children and Infants. Molecular therapy : the journal of the American Society of Gene Therapy. 2016;24(8):1470-7. Epub 2016/04/26. doi: 10.1038/mt.2016.83. PubMed PMID: 27109630; PubMed Central PMCID: PMCPMC5010143.
31. Kimani D, Jagne YJ, Cox M, Kimani E, Bliss CM, Gitau E, et al. Translating the immunogenicity of prime-boost immunization with ChAd63 and MVA ME-TRAP from malaria naive to malaria-endemic populations. Molecular therapy : the journal of the American Society of Gene Therapy. 2014;22(11):1992-2003. Epub 2014/06/17. doi: 10.1038/mt.2014.109. PubMed PMID: 24930599; PubMed Central PMCID: PMCPMC4188556.

32. Tapia MD, Sow SO, Lyke KE, Haidara FC, Diallo F, Doumbia M, et al. Use of ChAd3-EBO-Z Ebola virus vaccine in Malian and US adults, and boosting of Malian adults with MVA-BN-Filo: a phase 1, single-blind, randomised trial, a phase 1b, open-label and double-blind, dose-escalation trial, and a nested, randomised, double-blind, placebo-controlled trial. The Lancet Infectious diseases. 2016;16(1):31-42. Epub 2015/11/08. doi: 10.1016/s1473-3099(15)00362-x. PubMed PMID: 26546548; PubMed Central PMCID: PMCPMC4700389.
33. Vellinga J, Smith JP, Lipiec A, Majhen D, Lemckert A, van Ooij M, et al. Challenges in manufacturing adenoviral vectors for global vaccine product deployment. Human gene therapy. 2014;25(4):318-27. Epub 2014/03/07. doi: 10.1089/hum.2014.007. PubMed PMID: 24593243.
34. Dicks MD, Spencer AJ, Edwards NJ, Wadell G, Bojang K, Gilbert SC, et al. A novel chimpanzee adenovirus vector with low human seroprevalence: improved systems for vector derivation and comparative immunogenicity. PLoS One. 2012;7(7):e40385. Epub 2012/07/19. doi: 10.1371/journal.pone.0040385. PubMed PMID: 22808149; PubMed Central PMCID: PMCPMC3396660.
35. Chartier C, Degryse E, Gantzer M, Dieterle A, Pavirani A, Mehtali M. Efficient generation of recombinant adenovirus vectors by homologous recombination in Escherichia coli. J Virol. 1996;70(7):4805-10. Epub 1996/07/01. PubMed PMID: 8676512; PubMed Central PMCID: PMCPMC190422.
36. Havenga M, Vogels R, Zuijdgeest D, Radosevic K, Mueller S, Sieuwerts M, et al. Novel replication-incompetent adenoviral B-group vectors: high vector stability and yield in PER.C6 cells. The Journal of general virology. 2006;87(Pt 8):2135-43. Epub 2006/07/19. doi: 10.1099/vir.0.81956-0. PubMed PMID: 16847108.
37. Folegatti PM, Bellamy D, Roberts R, Powlson J, Edwards NJ, Mair CF, et al. Safety and Immunogenicity of a Novel Recombinant Simian Adenovirus ChAdOx2 as a Vectored Vaccine. Vaccines (Basel). 2019;7(2):40. doi: 10.3390/vaccines7020040. PubMed PMID: 31096710.
38. Vitelli A, Folgori A, Scarselli E, Colloca S, Capone S, Nicosia A. Chimpanzee adenoviral vectors as vaccines – challenges to move the technology into the fast lane. Expert Review of Vaccines. 2017;16(12):1241-52. doi: 10.1080/14760584.2017.1394842.
39. Folegatti PM, Bellamy D, Roberts R, Powlson J, Edwards NJ, Mair CF, et al. Safety and Immunogenicity of a Novel Recombinant Simian Adenovirus ChAdOx2 as a Vectored Vaccine. Vaccines. 2019;7(2). doi: 10.3390/vaccines7020040.
40. Afolabi MO, Tiono AB, Adetifa UJ, Yaro JB, Drammeh A, Nebie I, et al. Safety and Immunogenicity of ChAd63 and MVA ME-TRAP in West African children and infants. Molecular therapy : the journal of the American Society of Gene Therapy. 2016. doi: 10.1038/mt.2016.83. PubMed PMID: 27109630.
41. Bejon P, Mwacharo J, Kai O, Mwangi T, Milligan P, Todryk S, et al. A Phase 2b Randomised Trial of the Candidate Malaria Vaccines FP9 ME-TRAP and MVA ME-TRAP among Children in Kenya. PLoS Clin Trials. 2006;1(6):e29. PubMed PMID: 17053830.
42. Mensah VA, Gueye A, Ndiaye M, Edwards NJ, Wright D, Anagnostou NA, et al. Safety, Immunogenicity and Efficacy of Prime-Boost Vaccination with ChAd63 and MVA Encoding ME-TRAP against Plasmodium falciparum Infection in Adults in Senegal. PloS one. 2016;11(12):e0167951. doi: 10.1371/journal.pone.0167951. PubMed PMID: 27978537; PubMed Central PMCID: PMCPMC5158312 applications and patents on malaria vectored vaccines and immunisation regimes including the following (WO2008/122769, Adenoviral vector encoding malaria antigen; and WO 2008/122811 Novel adenovirus vectors). Egeruan Imoukhuede and Ines Petersen were employees of EVI at the time of the study, which supports the development and testing of malaria vaccines. Nicola Viebig is an employee of EVI and Odile Leroy is executive director of EVI. Authors from ReiThera (formerly Okairos) are employees of and/or shareholders in ReiThera, which is developing vectored vaccines for malaria and other diseases. Alfredo Nicosia was employed by ReiThera (formerly Okairos) at the time of the study. There are no further patents, products in development or marketed products to declare. This does not alter our adherence to all the PLOS ONE policies on sharing data and materials, as detailed online in the guide for authors.
43. Ogwang C, Afolabi M, Kimani D, Jagne YJ, Sheehy SH, Bliss CM, et al. Safety and immunogenicity of heterologous prime-boost immunisation with Plasmodium falciparum malaria candidate vaccines, ChAd63 ME-TRAP and MVA ME-TRAP, in healthy Gambian and Kenyan adults. PloS one. 2013;8(3):e57726.

doi: 10.1371/journal.pone.0057726. PubMed PMID: 23526949; PubMed Central PMCID: PMCPMC3602521.
44. Folegatti PM, Ewer KJ, Aley PK, Angus B, Becker S, Belij-Rammerstorfer S, et al. Safety and immunogenicity of the ChAdOx1 nCoV-19 vaccine against SARS-CoV-2: a preliminary report of a phase 1/2, single-blind, randomised controlled trial. Lancet. 2020;396(10249):467-78. Epub 2020/07/24. doi: 10.1016/s0140-6736(20)31604-4. PubMed PMID: 32702298; PubMed Central PMCID: PMCPMC7445431.
45. Soentjens P, De Koninck K, Tsoumanis A, Herssens N, Van Den Bossche D, Terryn S, et al. Comparative Immunogenicity and Safety Trial of 2 Different Schedules of Single-visit Intradermal Rabies Postexposure Vaccination. Clinical infectious diseases : an official publication of the Infectious Diseases Society of America. 2019;69(5):797-804. Epub 2018/12/20. doi: 10.1093/cid/ciy983. PubMed PMID: 30566636.
46. Kessels JA, Recuenco S, Navarro-Vela AM, Deray R, Vigilato M, Ertl H, et al. Pre-exposure rabies prophylaxis: a systematic review. Bulletin of the World Health Organization. 2017;95(3):210-9c. Epub 2017/03/03. doi: 10.2471/blt.16.173039. PubMed PMID: 28250534; PubMed Central PMCID: PMCPMC5328107.
47. Langedijk AC, De Pijper CA, Spijker R, Holman R, Grobusch MP, Stijnis C. Rabies Antibody Response After Booster Immunization: A Systematic Review and Meta-analysis. Clin Infect Dis. 2018;67(12):1932-47. Epub 2018/05/23. doi: 10.1093/cid/ciy420. PubMed PMID: 29788204.
48. Wieten RW, Leenstra T, van Thiel PP, van Vugt M, Stijnis C, Goorhuis A, et al. Rabies vaccinations: are abbreviated intradermal schedules the future? Clin Infect Dis. 2013;56(3):414-9. Epub 2012/10/09. doi: 10.1093/cid/cis853. PubMed PMID: 23042968.
49. World Health O. Rabies vaccines: WHO position paper, April 2018 – Recommendations. Vaccine. 2018;36(37):5500-3. doi: https://doi.org/10.1016/j.vaccine.2018.06.061.
50. Aubert MF. Practical significance of rabies antibodies in cats and dogs. Rev Sci Tech. 1992;11(3):735-60. Epub 1992/09/01. doi: 10.20506/rst.11.3.622. PubMed PMID: 1472723.
51. Tanzania Commission for AIDS (TACAIDS) ZACZ, National Bureau of, Statistics (NBS) OotCGSO, and ICF International,. Tanzania HIV/AIDS and Malaria Indicator Survey 2011-12. Dar es Salaam, Tanzania: TACAIDS, ZAC, NBS, OCGS, and ICF International., 2013.
52. Gibson BE, Todd A, Roberts I, Pamphilon D, Rodeck C, Bolton-Maggs P, et al. Transfusion guidelines for neonates and older children. Br J Haematol. 2004;124(4):433-53. PubMed PMID: 14984493.
53. Howie SR. Blood sample volumes in child health research: review of safe limits. Bulletin of the World Health Organization. 2011;89(1):46-53. doi: 10.2471/BLT.10.080010. PubMed PMID: 21346890; PubMed Central PMCID: PMCPMC3040020.
54. Services. DoANIoAaIDNIoHUDoHaH. Division of AIDS (DAIDS) Table for Grading the Severity of Adult and Pediatric Adverse Events. Division of AIDS National Institute of Allergy and Infectious Diseases National Institutes of Health US Department of Health and Human Services., 2017 July 2017 Report No.
55. US-FDA. Guidance for Industry: Toxicity Grading Scale for Healthy Adult and Adolescent Volunteers Enrolled in Preventive Vaccine Clinical Trials 2014 [updated 15 September 2014 23 October 2017]. Available from: https://www.fda.gov/biologicsbloodvaccines/guidancecomplianceregulatoryinformation/guidances/vaccines/ucm074775.htm.
56. WHO. PROPOSED INTERNATIONAL GUIDELINES ON ETHICAL ISSUES IN MEDICAL GENETICS AND GENETIC SERVICES: Report of a WHO meeting on Ethical Issues in Medical Genetics. 1998 15-16 December 1997. Report No.: Contract No.: WHO/HGN/GL/ETH/98.1.
57. Human Tissue Act 2004 2004 [cited 2017 23 October 2017]. Available from: http://www.legislation.gov.uk/ukpga/2004/30/contents.
58. Petrini C. "Broad" consent, exceptions to consent and the question of using biological samples for research purposes different from the initial collection purpose. Soc Sci Med. 2010;70(2):217-20. doi: 10.1016/j.socscimed.2009.10.004. PubMed PMID: 19853341.

17 APPENDIX A: SCHEDULE OF PROCEDURES

Groups AC1, AC2, AC3, AV1 (Adults, 18-45 years)		Screening		Vaccination and follow up																	Simulated PEP		
Study weeks relative to first vaccination		0	0	1							2	3	5	8	26	52	104	156	208	260	Varied*	Varied	Varied
Study days relative to first vaccination		-30 to 0	-30 to 0	0	1	2	3	4	5	6	7	14	28	56	182	365	730	1095	1460	1825	Varied*	Varied	Varied
Study Visit Code		SC1	SC2	V	V+1	V+2	V+3	V+4	V+5	V+6	V+7	V+14	V+28	V+56	V+182	V+365	V+730	V+1095	V+1460	V+1825	SPEP+0	SPEP+7	SPEP+14
Visit window (days)					+1	-1					-1/+3	-1/+3	-7/+2	+/-7	+/-14	-92/+181	-92/+181	-92/+181	-92/+181	-92/+181	-92/+181	+/-1**	+/-1**
Home Visits (community healthcare worker)						x	x	x	x	x													
Clinic Visits		x	x	x	x						x	x	x	x	x	x	x	x	x	x	x	x	x
Vaccination				x																	x		x
Enrolment				x																			
Group Allocation				x																			
**Clinical Assessment**																							
Informed Consent and eligibility confirmation		x		x																			
Medical history with demographic data		x																					
Photo taken and temporary ID card		x																					
Permanent ID Card				x																			
Physical Examination	Detailed	x																					
	Focused			x	x						x	x	x	x	x	x	x	x	x	x	x	x	x
Physical Assessments	Vital Signs	x		x	x						x	x	x	x	x	x	x	x	x	x	x	x	x
	Height	x																					
	Weight	x		x																	x		x
Adverse Events Data Collection	Solicited			x	x	x	x	x	x	x	x												
	Unsolicited			x	x	x	x	x	x	x	x	x	x	x	x	x	x	x	x	x	x	x	x
	SAE & New Medical Conditions			x	x	x	x	x	x	x	x	x	x	x	x	x	x	x	x	x	x	x	x
**Laboratory Assessment**																							
Haematology	RBC, HGB, Platelets, WBC with differential	0.5		0.5							0.5	0.5	0.5	0.5	0.5								
Biochemistry	ALT, Creatinine	1		1							1	1	1	1	1								
Parasitology	Thick blood smear (malaria)	0.5																					
Serology [HIV, HBV, HCV]		1																					
Immunology maximum blood volume to draw***				100	(50)						(50)	(100)	100	(50)	(50)	50	50	50	50	50	60	60	60
Urine Pregnancy [Females only]		x		x																			
Urine Dipstick [Blood, protein and glucose]		x																					
Total Blood Volume per visit (ml)		3	0	101.5	50	0	0	0	0	0	51.5	101.5	101.5	51.5	51.5	50	50	50	50	50	60	60	60
Cumulative Maximum Blood Volume (ml)		3	3	104.5	154.5	154.5	154.5	154.5	154.5	154.5	206	307.5	409	460.5	512	562	612	662	712	762	772	832	892

*=the SPEP+0 visit will replace the V+365. V+730. V+1095, V+1460 or V+1825 visit for each participant. **=window is relative to SPEP+0 visit. ***=where blood volumes are in paretheses, collection may not occur for all participants. Exploratory immunology may include assays of innate, and adaptive humoral and cellular responses using a variety of techniques applied to a subset of samples.

Groups PC1a, PC1b, PC2, PC3, PV1, PV2 (children 2 - 6 years)		Screening		Vaccination and follow up																	Simulated PEP		
Study weeks relative to first vaccination		0	0	1							2	3	5	8	26	52	104	156	208	260	Varied*	Varied	Varied
Study days relative to first vaccination		-30 to 0	-30 to 0	0	1	2	3	4	5	6	7	14	28	56	182	365	730	1095	1460	1825	Varied*	Varied	Varied
Study Visit Code		SC1	SC2	V	V+1	V+2	V+3	V+4	V+5	V+6	V+7	V+14	V+28	V+56	V+182	V+365	V+730	V+1095	V+1460	V+1825	SPEP+0	SPEP+7	SPEP+14
Visit window (days)					+1	-1					-1/+3	-1/+3	-7/+2	+/-7	+/-14	-92/+181	-92/+181	-92/+181	-92/+181	-92/+181	-92/+181	+/-1**	+/-1**
Home Visits (community healthcare worker)						x	x	x	x	x													
Clinic Visits		x	x	x	x						x	x	x	x	x	x	x	x	x	x	x	x	x
Vaccination				x							x~										x		x
Enrolment				x																			
Group Allocation				x																			
**Clinical Assessment**																							
Informed Consent and eligibility confirmation		x		x																			
Medical history with demographic data		x																					
Photo taken and temporary ID card		x																					
Permanent ID Card				x																			
Physical Examination	Detailed	x																					
	Focused			x	x						x	x	x	x	x	x	x	x	x	x	x	x	x
Physical Assessments	Vital Signs	x		x	x						x	x	x	x	x	x	x	x	x	x	x	x	x
	Height	x																					
	Weight	x		x																	x		x
	MUAC	x		x																			
Adverse Events Data Collection	Solicited			x	x	x	x	x	x	x	x												
	Unsolicited			x	x	x	x	x	x	x	x	x	x	x	x	x	x	x	x	x	x	x	x
	SAE & New Medical Conditions			x	x	x	x	x	x	x	x	x	x	x	x	x	x	x	x	x	x	x	x
**Laboratory Assessment**																							
Haematology	RBC, HGB, Platelets, WBC with differential	0.5		0.5							0.5	0.5	0.5	0.5	0.5								
Biochemistry	ALT, Creatinine	1		1							1	1	1	1	1								
Parasitology	Thick blood smear (malaria)	0.5																					
Serology [HIV, HBV, HCV]		1																					
Immunology maximum blood volume to draw***				20	(2)						(2)	(15)	10	(10)	(20)	25	25	25	25	25	25	20	25
Urine Dipstick [Blood, protein and glucose]		x																					
Total Blood Volume per visit (ml)		3	0	21.5	2	0	0	0	0	0	3.5	16.5	11.5	11.5	21.5	25	25	25	25	25	25	20	25
Cumulative Maximum Blood Volume (ml)		3	3	24.5	26.5	26.5	26.5	26.5	26.5	26.5	30	46.5	58	69.5	91	116	141	166	191	216	216	236	261

*=the SPEP+0 visit will replace the V+365. V+730. V+1095, V+1460 or V+1825 visit for each participant. **=window is relative to SPEP+0 visit. ***=where blood volumes are in paretheses, collection may not occur for all participants. Exploratory immunology may include assays of innate, and adaptive humoral and cellular responses using a variety of techniques applied to a subset of samples. ~=On Day7, PV2 group members only will receive a 2-site ID vaccination with IRV.

18 APPENDIX B: SAE REPORTING FLOW CHART

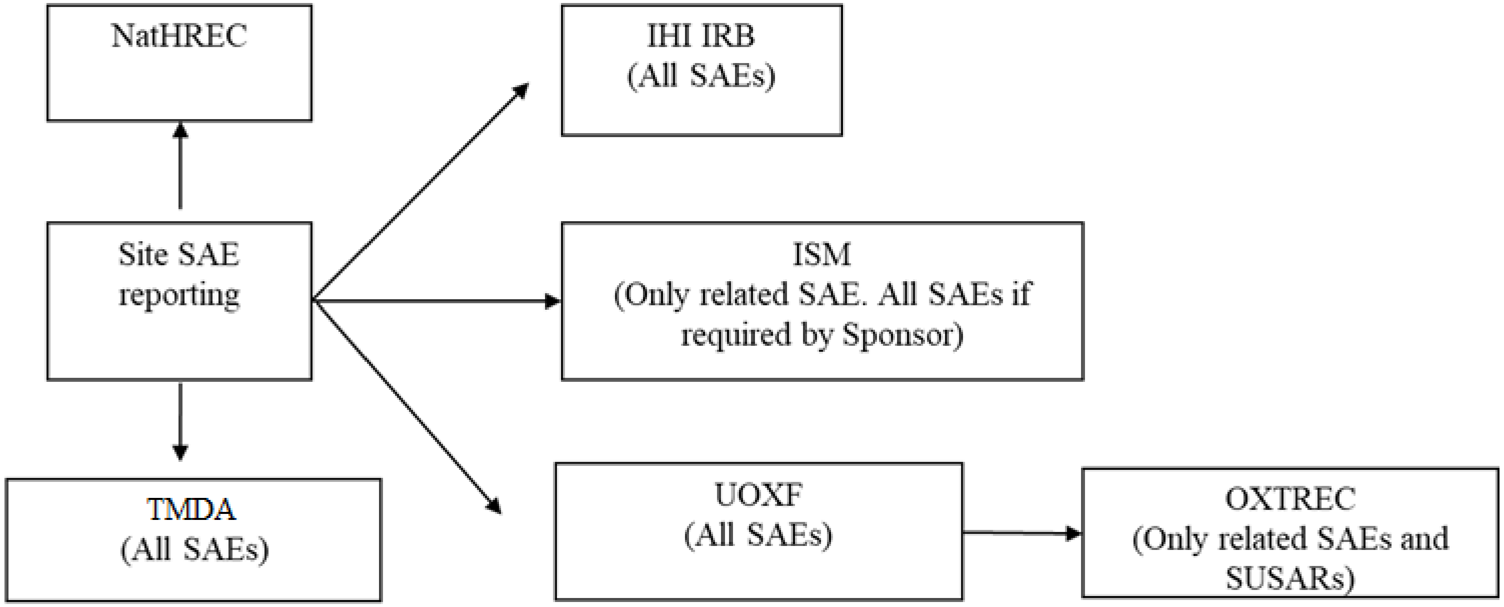

19 APPENDIX C: AMENDMENT HISTORY

Amendment No.	Protocol Version No.	Date issued	Author(s) of changes	Details of Changes made
	2.0	9th July 2020	Ally Olotu, Daniel Jenkins, Adam Ritchie	• Minor type-os fixed and minor stylistic changes.<br>• IRV to be used confirmed as VERORAB<br>• Actions to follow participant withdrawal updated.<br>• Protocol updated with procedures related to COVID-19.<br>• Sample storage and future use updated.<br>• Rationale for blood volumes collected updated.<br>• Statistical analysis considerations updated.<br>• Schedules of procedures tables updated.
	2.1	16th October 2020	Ally Olotu, Adam Ritchie	• Age of children changed to 2-6 years<br>• Include inclusion criteria for Groups 2a, 2b and 2c if completing EPI 6 months prior to enrolment.<br>• Schedules of procedures tables updated.
SA001	3.0	4th October 2021	Sandy Douglas, Ally Olotu, Adam Ritchie	• New, expanded trial design. Major changes include:<br>o Dose escalation in adult groups<br>o Increased number of adults receiving ChAdOx2 RabG<br>o Extra paediatric dose escalation group<br>o Increased number of children receiving ChAdOx2 RabG<br>o Increased number of children receiving single visit Verorab<br>o New paediatric comparator group receiving two-visit Verorab<br>• Rationale for new trial design

				• Confirmation the all participants in RAB001 have received their vaccine and completed safety follow-up, and the applicable safety report is completed and reviewed.<br>• Information on ChAdOx1 nCOV-19 and relevant experience added<br>• Updated visit schedule, including larger range of days for receipt of simulated PEP<br>• Updated safety reviewing information, including when reviews by the DSMB and CI/PI will be carried out<br>• Holding rules updated and represented in a table<br>• Updated inclusion and exclusion criteria<br>• Information related to management of COVID-19 added<br>• Allocation/randomisation procedure updated<br>• Optional provision of paracetamol updated<br>• Clarification that Verorab is the preferred IRV for the study, but that alternatives can be used if Verorab is unavailable<br>• Updated information on thawing and storage of IMP<br>• Statistical analysis consideration updated
SA002	4.0	4th May 2022	Adam Ritchie, Ally Olotu, Saumu Ahmed, Sandy Douglas	• New version and date.<br>• Section 4; Typo removed from table<br>• Section 5.1; changed to match section 7.5 and Appendix A, so that it is clear participants are seen in person on day 1 (not day 2).<br>• Section 5.1; clarified that the reporting of unsolicited symptoms is for 28 days following the day 0 vaccination, not each vaccination.<br>• Section 6.4; Typo in Adult inclusion criteria for BMI fixed, so is no 18-30 Kg/m^2 (was 18-35).<br>• Section 6.5: Some text coloured red in error changed to black

				• Section 7.5; changed to match Appendix A, so that the day 14 visit applies to all participants, not just those receiving ChAdOx2 RabG.<br>• Section 7.5; changed to match Appendix A, so that visits on days 56 and 182 include samples being taken for haematology and biochemistry.<br>• Appendix A; updated to match Section 7.5, so that participant body weight measurements will be carried out at the SPEP+0 and SPEP+14 visits.
SA003	5.0	6th February 2023	Adam Ritchie	• New version and date<br>• New trial design with extended follow up period. Major changes include:<br>o Participants able to opt in for an extended period of up to 5.5 years follow up<br>o Annual visits added during extended follow up<br>o Retention visits added<br>o Simulated PEP visit will occur instead of one of the annual visits during the extended follow up period<br>o Participants will be randomisated to received SPEP at the next available visit or at the end of the study<br>o Participants will attend annual visits after receiving SPEP until the study ends<br>o Study investigators and SMC may end the follow up period earlier than 5.5 years at their discretion<br>o Details of re-consent process<br>o Adult female participants who consent to extended follow-up required to use contraception during first year of study only.<br>• Rationale for new study design added<br>• Previous clinical experience with ChAdOx2 RabG updated<br>• All scheduled SMC and CI/PI data reviews have taken place

				• Update to incentives section<br>• New Schedules of Procedures added<br>• Clarification that only unreported SAEs are collected at visit after day 28 in section 7.5.

20 APPENDIX D: KEY ROLES AND GENERAL INFORMATION

**Trial Centre**	Ifakara Health Institute Clinical Trial Facility,<br>Bagamoyo Research and Training Centre<br>P.O. Box 74<br>Bagamoyo, Tanzania
**Principal Investigator (IHI):**	Ally Olotu MD DPhil<br>Ifakara Health Institute<br>P.O. Box 74<br>Bagamoyo, Tanzania<br>Tel: +255 718 927 104<br>e-mail: aolotu@ihi.or.tz
**Chief Investigator (UOXF)**	Alexander Douglas<br>The Jenner Institute<br>University of Oxford<br>Old Road Campus Research Building<br>Roosevelt Drive<br>Oxford<br>OX3 7DQ
**Sponsor:**	University of Oxford<br>Research Services<br>University Offices<br>Wellington Square<br>Oxford, OX1 2JD<br>United Kingdom<br>Phone number: +44 (0)1865 282585<br>e-mail: oxtrec@admin.ox.ac.uk
**Funder:**	Medical Research Council<br>14th floor

	One Kemble Street<br>London<br>WC2B 4AN<br>United Kingdom
**Ethics Committees**	Ifakara Health Institute IRB<br>P.O. Box 78373<br>Dar es Salaam, Tanzania<br>Tel: +255 (0) 22 2774714,<br>Fax: + 255 (0) 22 2771714<br>e-mail: irb@ihi.or.tz<br><br>National Health Research Ethics Sub-Committee (NatHREC)<br>National Institute for Medical Research<br>P.O. Box 9653<br>Dar es Salaam, Tanzania<br>Tel: +255 22 2121400<br>Fax: 255 22 2121360<br>e-mail: ethics@nimr.or.tz<br><br>Oxford Tropical Research Ethics Committee (OxTREC)<br>University of Oxford<br>Research Services<br>University Offices<br>Wellington Square<br>Oxford, OX1 2JD<br>United Kingdom<br>Tel: +44 (0)1865 282585<br>e-mail: oxtrec@admin.ox.ac.uk

**Regulatory Authority**	Tanzanian Medicines and Medical Devices Authority<br>Postal Address P.O. Box 1253, Mwanza Avenue, Block T, Plot No. 6, Dodoma,Tanzania<br>Phone: +255 22 2450512 / 2450751 / 2452108<br>Fax: No: +255 22 2450793<br>Email Address: info@tfda.go.tz
**Laboratories**	1. Bagamoyo Research and Training Centre Laboratory, Ifakara Health Institute, Bagamoyo Tanzania.<br>2. Jenner Institute laboratories, Centre for Clinical Vaccinology & Tropical Medicine, University of Oxford, Churchill Hospital, Old Road, Oxford, OX3 7LJ, United Kingdom.<br>3. The Wistar Institute of Anatomy and Biology 3601 Spruce Street, Philadelphia, PA 19104 USA

RAB002 Statistical Analysis Plan

Clinicaltrials.gov number: NCT04270838

Version 2.0

9 August 2022

To be filed in a trial master file

1 Related documents

Protocol version 5.0

Laboratory analysis plan version 5

BRTC Data Management Standard Operational Manual Version 5.0

2 SAP version history

Version number	Author	Date	Relationship to data locks/analyses	Changes
1.0	Alexander Douglas	9 August 2022	Preceding submission of first samples for VNA analysis	New document
2.0	Alexander Douglas	09 September 2023	Associated with SA003 (extending follow up). Preceding reporting of any paediatric VNA data or any 6 month follow-up adult data	Addition of information regarding randomisation to timing of SPEP. No other changes required.

3 Roles, responsibilities and signatures

23 August 2023

Dr Alexander Douglas, University of Oxford, Chief Investigator & author of SAP

09 September 2023

Dr Ally Olotu, Ifikara Health Institute, Principal Investigator, reviewer of SAP & responsible for data management & execution of analysis plan.

4 Introduction & objectives

This document is designed to be read in conjunction with the study protocol and to avoid duplication of information already contained in the protocol. It has been written with reference to the relevant sections of recognised guidance[1].

Rationale and background information relating to the RAB002 study is provided in the trial protocol.

4.1 Study objectives & outcome measures

The protocol-stated objectives and outcome measures are as follows

	Objectives	Outcome Measures
**Primary**	To determine the safety and tolerability of ChAdOx2 RabG in healthy adults (18-45 years) and children (2-6 years) residing in a rabies endemic country.	• Local and systemic solicited AEs<br>• Unsolicited AEs<br>• Laboratory AEs<br>• Post-vaccination serious adverse events during the study period.
**Secondary**	To assess the immunogenicity of ChAdOx2 RabG and single-visit 2-site intradermal vaccination with licensed rabies vaccine in adults and children, in a rabies endemic country	• Rabies virus neutralising antibody (VNA) as assessed by rapid fluorescent focus inhibition test (RFFIT)
**Exploratory**	To assess other anti-rabies antibody titres measured by ELISA, T cell responses were measured by ELISpot and other assays.	• Cellular and humoral immunity to rabies antigen

Analysis relating to the primary objective (safety) will be purely descriptive and is briefly covered in this SAP.

The secondary objective encompasses the characterisation of VNA at several timepoints with varying clinical significance. Within this characterisation, several precisely defined endpoints can be defined (i.e., VNA at a given time point). Based upon these endpoints, primary, secondary and exploratory immunological analyses will be performed. This SAP primarily relates to these immunological analyses.

The protocol states that immune responses other than VNA are exploratory study endpoints. Analyses of these non-VNA responses are not discussed in this statistical analysis plan.

4.2 Immunological endpoints, hypotheses and analyses

With respect to VNA, we hypothesize that

1. [Early immunogenicity] **Early (day 28) VNA induction by ChAdOx2 RabG will be superior to that of a single dose of licensed rabies vaccine.** This will be tested as a secondary immunological analysis.

2. [Unboosted VNA maintenance] **Maintenance of VNA over a period of 1-5 years after ChAdOx2 RabG will be superior to that after a single dose of licensed rabies vaccine**. Unboosted maintenance will be the co-primary immunological endpoint and the superiority of maintenance over a period of one year will be tested by the primary immunological analysis.

3. [Overall protection] **ChAdOx2 RabG would achieve ≥90% protection for ≥1 year among vaccinees able to access single-dose PEP after any exposure**: This will be reported (alongside unboosted VNA maintenance) as the co-primary immunological endpoint but will not be subject to hypothesis testing. The definition of overall protection is given in section 11.2.3.

4. Either single visit or two-visit regimes using licensed rabies vaccine would also achieve ≥90% protection for ≥1 year among vaccinees able to access single-dose PEP after any exposure.

Paediatric and adult data will be analysed and reported similarly, but separately.

An additional paediatric comparator group receiving two doses of licensed rabies vaccine is included in the design. Data from this group will be reported in connection with hypothesis #4 above. Any comparisons of this group with ChAdOx2 recipients will be regarded as exploratory.

Version 4.0 of the protocol allows for follow-up of participants for up to 1 year following primary vaccination. Future protocol amendments may allow for longer follow-up, and the SAP is written to allow for any such amendments.

5 Trial design

See protocol for details.

6 Trial population

Inclusion/exclusion criteria are described in the protocol

The CONSORT diagram will present withdrawals and losses to follow-up between vaccination and each data cut-off.

Baseline characteristics will be tabulated to include at a minimum the following for each study group: -numbers of males and females

-age (median and range)

-ethnicity (African, Asian, Caucasian, mixed or other)

-receipt of previous COVID-19 vaccination (none, adenovirus-vectored, other), and for any recipients of adenovirus-vectored vaccination, time since receipt (median and range)

7 Treatment allocation

7.1 Allocation to groups

Allocation to groups, including randomisation where applicable, is outlined in the protocol and SOPs for randomisation. Block sizes of 4 (adults) and 7 (children) will be used for most stages of the study. The only exception is in stages 4 and 9, where the block size will be determined by the local site statistician following the selection of the adult and paediatric preferred doses of ChAdOx2 RabG.

No stratification or minimisation was used. Allocation was not concealed from trial staff or participants but is concealed from laboratory staff performing VNA assays.

7.2 Allocation to different timing of SPEP administration

Allocation of participants returning a VNA result of <0.5 IU/mL to SPEP at the next clinic visit after availability of the result or at the end of the study is outlined in the protocol and IHI Rabies Study Operational Manual (IHIBSG_RABIES). Randomization will be stratified by age group and baseline vaccine regime (adult preferred dose of ChAdOx2, adult non-preferred dose of ChAdOx2, adult single-visit Verorab, paediatric preferred dose of ChAdOx2, paediatric non-preferred dose of ChAdOx2, paediatric single-visit Verorab, paediatric two-visit Verorab). Within each of these groups, further stratification will take place by inclusion in the “immune subset”, as outlined in the current version of the Laboratory Analysis Plan. Randomisation will use a block size of 2 or 4 at the discretion of the local statistician. Allocation will not be concealed from trial or laboratory staff.

8 Sample size

The primary safety analysis will be a descriptive analysis of the safety data, without any hypothesis testing or statistical inference, in all participants who have received ChAdOx2 RabG (safety dataset). The sample size has therefore been chosen based upon the investigators’ opinion of what constitutes an adequate number of participants to facilitate further clinical evaluation in phase II trials.

9 Timing of outcome assessments

Adult and paediatric data sets will be analysed separately. For each, the primary analysis will include safety and VNA data collected up to the nominal 1-year follow-up and any SPEP data available at a cut-off date to be set at the investigators’ discretion, 1-2 years after the final day 0 visit (for an adult or child, as applicable).

The study may be terminated at any point after the primary analysis on grounds of futility, if this is permissible within the then-current protocol, and if the investigators and DSMB are in agreement that the data obtained does not support the onward development of the product.

Subject to protocol amendment additional VNA analyses will be performed (again with separate analyses and cut-offs for adult and paediatric data sets):

-If immunological follow-up continues to year 2 follow-up, with a cut-off date 2-3 years after the final day 0 visit,

-If immunological follow-up continues to year 5 follow-up, with a cut-off date 5-6 years after the final day 0 visit.

-These possible later analyses are referred to henceforth as ‘2-year’ and ‘5-year’ analyses.

Investigators will not be blinded to data accumulating before specified analyses, and preliminary analyses may be performed at the investigators’ discretion (for example a near-complete day 28 early VNA data set will be available sometime before the primary analysis timepoint).

10 Statistical principles

10.1 Uncertainty

Partly because the sample size was not chosen based on the power of hypothesis-testing relating to immunogenicity, emphasis in reporting will be placed on estimation (confidence intervals) rather than hypothesis testing. Where confidence intervals are reported, 95% intervals will be provided.

Hypothesis tests will use $\alpha=0.05$ and will be two-tailed tests of superiority unless otherwise specified.

No adjustment for multiple testing is planned, as discussed in the section describing analyses.

10.2 Adherence and deviations

Adherence to the intervention will be defined as receipt of the primary vaccination course (one or two doses) as allocated.

The following protocol deviations will be reported with the analysis:

-serious breaches of GCP

-loss to follow-up, or other reasons for lack of a VNA result or withdrawal of a participant from VNA analysis, including receipt of a non-study rabies vaccine.

10.3 Analysis populations

Methods for dose escalation decisions are stated in the protocol.

The protocol states that volunteers lost to follow-up between vaccination and study day 7 (i.e., before completion of the core safety data collection period) will be replaced with the next available volunteer.

All participants receiving the preferred dose of ChAdOx2 RabG will be combined (e.g., groups AC2 and AC3) and treated/reported as a single group for all analyses.

10.3.1 Safety population

Safety data will be presented for all volunteers receiving the day 0 vaccination per protocol, including any available data from volunteers replaced by the above criterion.

Data from participants receiving non-preferred doses will be presented separately from the main safety analysis. No data will be presented for any volunteers who did not receive any study vaccination.

10.3.2 Immunogenicity populations

Pre-specified VNA analyses will include all volunteers vaccinated per protocol, other than those receiving non-preferred doses of ChAdOx2 RabG. Participants receiving non-preferred doses will be excluded from these analyses but may be included in exploratory analyses.

Any individuals in group PV2 who do not receive the second dose of Verorab will be considered to be part of group PV1 for purposes of immunological analysis.

Any volunteers who report receiving a non-study rabies vaccine will be excluded from all analyses following the receipt of the non-study vaccine but included in all analyses preceding receipt.

10.3.2.1 Early VNA population

Individuals fulfilling the above criteria for whom a day 28 VNA result is available.

10.3.2.2 VNA maintenance population

Individuals fulfilling the above criteria for whom a day 365 VNA result is available at the data cut-off (for the primary VNA analysis).

Subject to protocol amendment: further analyses will include individuals fulfilling the above criteria for whom a day 730 VNA result is available at the data cut-off (for the year 2 VNA analysis), or a day 1825 VNA result is available at the data cut-off (for the year 5 VNA analysis).

10.3.2.3 Boosted VNA population

All individuals fulfilling the above criteria for whom a SPEP+7 VNA result is available at the applicable data cut-off.

11 Analysis

Timepoints and analysis populations are stated in sections 9 and 10.3 respectively, including that adult and paediatric populations will be analysed separately.

11.1 Safety

A purely descriptive safety analysis will be reported. This will include

-breakdown of solicited AEs up to day 7 by event type, group, day and severity

-tabulation of unsolicited AEs up to day 28 by group, severity, assessed relatedness, and MedDRA SOC/PT.

-tabulation of laboratory AEs up to day 28 by group and event type.

-line listing of SAEs and AESIs

Non-serious solicited AEs after the 2nd dose of Verorab in group PV2 will not be reported.

11.2 Immunogenicity

Unless otherwise stated the estimand will be VNA, expressed using international units per mL (IU/mL)

VNA is a discrete quantitative variable derived from the greatest dilution in a 3-fold series, achieving at least 50% neutralization of rabies virus. Negative values are possible.

The distribution of all VNA results (combining all groups and all timepoints other than day zero) in the RAB001 study is shown:

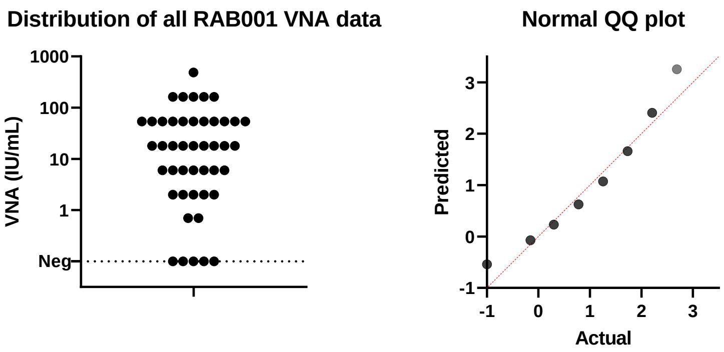

This distribution does not deviate significantly from lognormal according to a d'Agostino-Pearson normality test, although it does deviate from lognormality according to other tests and the possibility of negative results suggests a fairly high likelihood of non-normal distribution in future data sets. The standard deviation of $\log_{10}$(VNA) in this data set is 0.98.

It would conceptually be possible for virus neutralization to be expressed instead as a continuous variable (IC50 or areas under the curve[2]), which may increase the statistical power of comparisons, but this would require a change in the method of data collection/recording in the assay, is non-standard, and is not planned for this study.

11.2.1 Early VNA (day 28)

11.2.1.1 Descriptive

For each group, show all VNA values graphically, and report as summary measures the proportion with VNA>0.5%, geomean, 95% CI of geomean, and median / IQR / range of VNA.

Both median and geomean will be reported as the median will be robust to the fairly likely non-normal and non-continuous distribution, but the geomean is commonly used in the field so may be valuable for readers making a comparison to other studies.

11.2.1.2 Comparative

We will perform a two-tailed test of the superiority of VNA in recipients of the preferred dose of ChAdOx2 RabG *versus* recipients of a single dose of Verorab.

For each comparison:

-Examine the distribution of VNAs, with and without ladder-of-powers transformations (including log transformation).

-Perform the d'Agostino-Pearson test for normality after chosen transformation and based upon this choose a t-test or Mann-Whitney test.

As stated in section 8, the sample size for the study was selected based on suitability for the primary objective (safety) rather than the statistical power of immunological analyses.

The following exploratory analyses may be performed:

-Comparison of paediatric ChAd *vs* paediatric 2-dose Verorab.
-The above comparisons using data including participants at all ChAd dose levels (not just the preferred dose)
-Comparison between vaccines of peak early VNA (including VNA at days 14 and 56), given the theoretical possibility of different antibody kinetics between the two vaccines invalidating a comparison based on any single timepoint.
-Paediatric data may be analysed considering age as a covariate

11.2.2 Unboosted maintenance analysis

This is a co-primary immunological outcome measure and will be subject to the primary immunological hypothesis test.

The primary analysis will use data from the day 365 visit.

Subsequent analyses will be performed with day 730 data (for the year 2 VNA analysis), or day 1825 data (for the year 5 VNA analysis).

11.2.2.1 Descriptive

Present VNA data from the day 365 / 730 / 1825 visit as described above for the day 28 VNA. In addition, including VNA data from all pre-SPEP timepoints available:

-Report the proportion of participants in each group with VNA>0.5 IU/mL as a survival curve.

-Report kinetic of VNA responses in each group graphically

11.2.2.2 Comparative

At each data cut-off timepoint:
The main analysis will be a two-tailed test of the superiority of VNA in recipients of the preferred dose of ChAdOx2 RabG *versus* recipients of a single dose of Verorab. This will be performed as described for the early VNA analysis, including possible additional exploratory analyses.

As stated in section 8, the sample size for the study was selected based on suitability for the primary objective (safety) rather than the statistical power of immunological analyses.

Additional analyses which may be performed as exploratory, using alternative estimands:

-Analysis of the area under the curve of VNA *vs* time was considered for the primary analysis but will be performed as exploratory. This would have the advantage of a continuous distribution and possibly reduced noise but would be more vulnerable to missing VNA data.

-Log-rank survival analysis of the proportion of volunteers with VNA >0.5 IU/mL over time.

11.2.3 Analysis of response to SPEP / boosted protection & overall protection after ChAdOx2 RabG

This is a co-primary immunological outcome measure but will not be subject to hypothesis testing.

11.2.3.1 Descriptive only

At each data cut-off point for each group, report

a) proportion of those whose VNA had dropped to <0.5 IU/mL who attain VNA >0.5 IU/mL at SPEP+7.
b) Total proportion expected to be protected by a regime of 'single-visit PrEP for all, single-visit PEP when needed'

In calculating the proportion expected to be protected:
The proportion protected will be defined as the sum of
-those with unboosted VNA ≥0.5 IU/mL over the period in question, plus
-those whose VNA was <0.5 IU/mL (either due to failure of initial seroconversion or waning of responses) after ChAdOx2 RabG but for whom VNA is ≥0.5 IU/mL at the SPEP +7 (-1/+3) day visit (i.e. within around 7 days of administration of a simulated post-exposure prophylaxis [SPEP] booster).

The proportion unprotected will be defined as those whose VNA was <0.5IU/mL after ChAdOx2 RabG and remains <0.5 IU/mL at the SPEP+7 (-1/+3) day visit.

95% confidence intervals for these proportions will be reported using the Wilson/Brown hybrid method as implemented in Prism 9 (GraphPad software)[3,4].

11.2.4 Analysis of overall protection in groups AV1, PV1 and PV2 (i.e. receiving licensed rabies vaccine at baseline)

Descriptive analyses will be performed and reported for each group, as described in section 11.2.3.1 for groups receiving ChAdOx2 RabG at baseline.

11.3 Missing data

Missing data will not be imputed with the following exception(s)

-linear interpolation of a single missing VNA data point for a given volunteer, >6 months after primary vaccination, for purposes of any exploratory area under the curve analyses only

-for estimation of the total proportion expected to be protected at a given data cut-off, the proportion of recipients of SPEP with SPEP+7 VNA ≥0.5 IU/mL will be assumed to apply to any individuals eligible for SPEP (i.e., VNA<0.5 IU/mL at the last cut-off) but for whom SPEP+7 data is not available. Excluding these individuals from the analysis would bias the estimate of the proportion protected.

11.4 Software, code and report template

Analyses will be performed using Excel, GraphPad Prism, Stata or R.

Subsequent versions of this SAP may include code and report templates.

12 Reference

1. Homer V, Yap C, Bond S, et al. Early phase clinical trials extension to guidelines for the content of statistical analysis plans. *BMJ* 2022; **376**: e068177.
2. Yu X, Gilbert PB, Hioe CE, Zolla-Pazner S, Self SG. Statistical approaches to analyzing HIV-1 neutralizing antibody assay data. *Stat Biopharm Res* 2012; **4**(1): 1-13.
3. GraphPad Software. Three methods for computing the CI of a proportion. https://www.graphpad.com/guides/prism/latest/statistics/stat_three_methods_for_computing_th.htm (accessed 10/8/22.
4. Brown LD, Cai TT, DasGupta A. Interval Estimation for a Binomial Proportion. *Statistical Science* 2001; **16**(2): 101-33, 33.
