# Supplementary material for: Safety and immunogenicity of a single-dose adenovirus-vectored rabies vaccine over 1 year in adults and children in Tanzania: interim data from an ongoing, partly randomised, controlled, phase 1b/2 trial
Source: Lancet Infect Dis. 2026 Aug;26(8):843–57. doi: 10.1016/S1473-3099(26)00071-X (PMC13391811; doi:10.1016/S1473-3099(26)00071-X)
Supplement: Equitable Partnership Declaration [file mmc3.pdf]

THE LANCET
Infectious Diseases

Supplementary appendix 3

This Equitable Partnership Declaration (EPD) was submitted by the authors, and we reproduce it as supplied. It has not been peer reviewed. *The Lancet*'s editorial processes have not been applied to the EPD.

Supplement to: Ritchie AJ, Hassan O, Urasa N, et al. Safety and immunogenicity of a single-dose adenovirus-vectored rabies vaccine over 1 year in adults and children in Tanzania: interim data from an ongoing, partly randomised, controlled, phase 1b/2 trial. *Lancet Infect Dis* 2026; published online April 28. https://doi.org/10.1016/S1473-3099(26)00071-X.

Equitable Partnership Declaration

If any questions do not apply to your study, please indicate "N/A" for "not applicable.
For more information on how to complete this form see the Information for Authors document.

Researcher considerations

1. Please detail the involvement that researchers who are based in the country or countries of study had during a) study design; b) clinical study processes, such as processing blood samples, prescribing medication, or patient recruitment; c) data interpretation; and d) manuscript preparation, commenting on all aspects. If they were not involved in any of these aspects, please explain why.

   This should include a thorough description of their leadership roles in the study. Are local researchers named in the author list or the acknowledgements, or are they not mentioned at all (and, if not, why)? Please also describe the involvement of early career researchers based in the location of the study. Some of this information might be repeated from the Contributors section in the manuscript. Note: we adhere to ICMJE authorship criteria for naming authors on a paper.

**a) Study design:**<br>The principal investigator was involved in the conceptualization, designing of the study and protocol preparation and is among the protocol authors. The study design and protocol have since undergone amendments to extend the follow-up duration, the principal investigator and trial clinical lead were also involved in the trail redesign and protocol amendment.
**b) Clinical study processes:**<br>The clinical and laboratory operational aspects of the study were executed entirely by the local research team. This includes the trial approval applications and engagement with regulatory and ethical bodies, community engagement activities, participant recruitment, clinical management of trial participants, preparation and review of trial safety reports, handling and processing of trial samples before shipping and the running of laboratory tests and assays planned in the trial. The lead study clinician (an early career researcher, lead and supervised the clinical operations of the trial, laboratory operations were also overseen by early career researchers)
**c) Data interpretation:**<br>The interpretation of data in trial safety reports and the manuscript involved the principal investigator and the lead study clinician from the local research team. Interpretation of data and deliberation on strategy and future development plans of the investigational product was also done in conjunction with the principal investigator and the lead study clinician.
**d) Manuscript preparation:**<br>Manuscript review before submission involved local researchers and was coordinated by the lead study clinician for the local research team, local researchers have also been listed as authors on the manuscript.

2. How was funding used to remunerate and enhance the skills of researchers in the countries of study? And how was funding used to improve research infrastructure at the study sites?

   Potentially effective investments into long-term skills and opportunities within local institutions could include training or mentorship in analytical techniques and manuscript writing, opportunities to lead all or specific aspects of the study, financial remuneration rather than requiring volunteers, and other professional development and educational opportunities.

   Improvements to research infrastructure could include funding extended trial designs (eg, platform trials), establishment of long-term contracts for research staff, building research facilities, and setting up local control of funding allocation.

**Skills:** The project has made efforts to invest in the local research personnel at the Ifakara Health Institute (IHI) through a variety of channels. The project has been fully costed to provide salaries for IHI researchers for the 6.5 years it will run. It has provided funding and other in-kind contributions to support early career researches to attend various training opportunities including training at the sponsoring institution, fellowship programs and to attend and present trial related results at national and international conferences.<br>The project has also invested in leadership development of early career researchers by platforming them through opportunities to lead in various aspects such as clinical operations and grant application efforts for further development of the product /research in the disease area.<br>The project includes regular Teams calls between colleagues in Oxford and the clinical and laboratory teams at IHI, and two visits by the Oxford team to IHI to support development of the project and the team. For example, work in ELISAs by the IHI laboratory team includes significant mentorship from the Oxford team, including on experimental design, quality assurance, quality control, and data analysis.<br>Building on this project, the Oxford and IHI teams are setting up a new trial to look at single-visit rabies PEP, and have co-submitted an application to a major European funder that includes significant funding and support for career development of the IHI team.
**Research infrastructure:**<br>The project has supported expansion of the IHI central procurement processes for laboratory consumables. The length of follow-up (5.5 years) has supported long-term contracts and career planning. As part of the co-submitted funding application mentioned above, there are plans to expand and strengthen trials capabilities at IHI through a supported first-in-human trial of an African manufactured rabies vaccine, and the development of two platform trial programmes for rabid development of novel rabies vaccine candidates and co-administration of rabies vaccines with other vaccines.

3. How did you safeguard the researchers who implemented the study?

   Please describe how you guaranteed safe working conditions for study staff, including provision of appropriate personal protective equipment, protection from violence, and prevention of overworking.

The study was executed by the clinical research unit at an established and reputable research institution that adheres to international standards that define working conditions for the institution employees. In addition, provisions were made within the study budget to ensure the availability of all trail specific protective equipment and standard operating

procedures were developed together with the local research team to guide the handling of the sensitive investigational materials.

Benefits to the communities and regions of study

4. How does the study address the research and policy priorities of its location?

   How were the local priorities determined and then used to inform the research question? Who decided which priorities to take forward? Which elements of the study address those priorities?

This is an early phase clinical trial of a novel rabies vaccine. As discussed by several peer reviewers of the current manuscript, the outcome measures and linked research questions are very much determined by the field and WHO expert panel; safety, and immunogenicity as measured by the virus neutralisation assay, especially following simulated PEP. Meeting these goals has always been the priority of the Oxford team developing the novel ChAdOx2 RabG vaccine.

When the opportunity arose for Oxford to take their novel vaccine candidate forward, they engaged with multiple potential trial sites across Africa. The IHI team were enthusiastic about expanding the scope of their rabies work into clinical trials for new vaccines. Previously, rabies vaccines had been provided as a control arm in other clinical trials, and were perceived locally as a benefit of trial involvement. IHI also has a strong interest in rabies, especially in relation to education, dog vaccination, and access to vaccines.

During the set up of the trial, the IHI and Oxford teams worked together to identify what really would be most valuable to the local communities and comparable locations. This led to two shifts in the trial based on this co-assessment of priorities;
1. the study follow-up period was extended from 1.5 to 5.5 years, as it was felt that immunogenicity following sPEP years after primary vaccination would support future policy changes related to the provision of PrEP.
2. engagement with GAVI by the IHI team emphasised the importance of improving access to PEP, which led extra, early samples from the ChAdOx2 RabG recipient groups being run in the VNA assay to reveal more on the speed of seroconversion. There results (presented in the paper) have led to the establishment of the RAB003 trial at IHI to explore the possibility of a new, single-visit PEP regimen.

5. How will research products be shared in the community of study?

   For instance, will you be providing written or oral layperson summaries for non-academic information sharing? Will study data be made available to institutions in the region(s) of study? The Lancet Global Health *encourages authors to translate the summary (abstract) into relevant languages after paper editing; do you intend to translate your summary?*

As part of the study close out activities, plans are in place for results from the study to be summarised and presented in a lay manner to the study participants and/or their guardians.

6. How were individuals, communities, and environments protected from harm?

a) *How did you ensure that sensitive patient data were handled safely and respectfully? Was there any potential for stigma or discrimination against participants arising from any of the procedures or outcomes of the study?*

All trial data was handled in accordance with ICH-GCP guidelines. The local research team is responsible for handling the trial data. Local standard operating procedures and national regulations have been observed in ensuring that data are accurately collected and securely stored on accesses restricted servers and that data will be pseudonymized before being sharing with sponsor or any sanctioned external parties. Owing to the disease and field of research, there was limited potential for stigma or discrimination arising from study procedures, nonetheless efforts were still made by investigators to ensure safeguarding of all participant data.

b) *Might any of the tests be experienced as invasive or culturally insensitive?*

There were no tests within the trial that were determined to be invasive or culturally sensitive, this determination was made at different stages prior to the trial initiation by the local research team, the national and the institutional ethical review boards and together with community members as part of community engagement activities prior to recruitment.

c) *How did you determine that work was sensitive to traditions, restrictions, and considerations of all cultural and religious groups in the study population?*

The study design was conceptualised and prepared together with members of the local research team to ensure sensitivity to local traditions, religions and culture. The planned study activities were then reviewed by ethical bodies to further ensure observation of cultural and religious norms. Community-based sensitization meetings, were held which were open to the broader public, including community leaders and elders where community members engaged directly with the investigators and were able to voice their concerns and questions to the local research team.

d) *Were biowaste and radioactive waste disposed of in accordance with local laws?*

All biowaste from the trial activities were disposed as per the national ethical and regulatory guidelines.

e) *Were any structures built that would have impacted members of the community or the environment (such as handwashing facilities in a public space)? If so, how did you ensure that you had appropriate community buy-in?*

There were no structures built as part of the trial.

f) *How might the study have impacted existing health-care resources (such as staff workloads, use of equipment that is typically employed elsewhere, or reallocation of public funds)?*

Healthcare resources in the trial area were not impacted. The local researchers are dedicated staff at the clinical research unit, all equipment used as part of the clinical and laboratory trial activities are part of the clinical research unit infrastructure which is independent from the public health-care services. The trial was funded by the UK medical research council.
During the trial, resources from the trial were used to save the life of a local boy who had been bitten by a dog suspected of having rabies. At the time, no rabies vaccines could be procured locally in the time-frame needed, and doses of Verorab that were intended for the clinical trial were released for emergency use.

7. Confirm that local ethics review was sought, and please provide the approval number. If not sought, please explain why.

Local ethics reviews were sought at both the institutional and national levels prior to the start of any trial activities. In addition, permission was also sought from the president's office, regional administration and local government which manages the regional and district health services before the local research team approached the community to initiate recruitment activities.
Approval references from the
Institutional review board (IHI IRB) reference:12-2021
National Institute of Medical Research (NIMR) MIMR/HQ/R.8a/Vol.IX/3552

Secondary analyses

8. Have the data analysed in your study been extracted from another source, such as a national survey, rather than being directly collected by the authors of this paper?

No

If the authors of this paper were not involved in data collection, how were the findings interpreted with sufficient contextual knowledge?
The Lancet Global Health *believe contextual understanding is crucial for informed data analysis and interpretation.*

N/A

---

9. Please provide the title (eg, Dr/Prof, Mr/Mrs/Ms/Mx), name, and email address of an author who can be contacted about this statement.

Name: Dr Omary Hassan
Email: ohassan@ihi.or.tz

10. Finally, please provide the title and name of an author from one country of study who has seen and approved this form.

**Name:** Dr Omary Hassan
